# Insight of UV-vis spectra and atmospheric implication for the reaction of ˙OH radical towards glyphosate herbicide and its hydrates†

**DOI:** 10.1039/d1ra01591e

**Published:** 2021-05-04

**Authors:** Olivier Holtomo, Manain Derrick Mbigah, Mama Nsangou, Ousmanou Motapon

**Affiliations:** Department of Physics, Faculty of Science, University of Bamenda Bambili P. O. Box 39 Cameroon holtomoo@gmail.com; Department of Physics, Faculty of Science, University of Maroua Maroua P. O. Box 814 Cameroon; Department of Physics, Higher Teacher's Training College, University of Maroua Maroua P. O. Box 46 Cameroon; Department of Physics, Faculty of Science, University of Ngaoundéré Ngaoundéré P. O. Box 454 Cameroon; Laboratory of Fundamental Physics, Faculty of Science, University of Douala Douala P. O. Box 24157 Cameroon

## Abstract

The rate constant of the reactions of ˙OH radicals with glyphosate (GPS) and its hydrates (GPS(H_2_O)_*n*=1–3_) were evaluated using the dual method M06-2X/6-311++G(df,p)//6-31+G(df,p) over the temperature range of 200–400 K. The results served to estimate the atmospheric lifetime along with the photochemical ozone creation potential (POCP). The calculations yielded an atmospheric lifetime of 2.34 hours and a POCP of 24.7 for GPS. Upon addition of water molecules, there is an increase of lifetime and decrease of POCP for water monomer and water dimer. The POCP for water trimer is slightly above the gaseous GPS. However, the POCPs of GPS and its hydrates are comparable to that of alkanes. The GPS and its hydrates were found to be a potential reservoir of CO_2_. The acidification potential (AP) of GPS was found to be 0.189 and decreases upon addition of water molecules. This shows negligible contribution to rain acidification as the AP is less than that of SO_2_. The UV-vis spectra were attained using the M06-L/6-311++G(3df,3pd) method and cover the range 160–260 nm which fits well with experiment.

## Abstract 2

The rate constant of the reactions of ˙OH radical with glyphosate (GPS) and its hydrates (GPS(H_2_O)*_n_*_=1–3_) were evaluated using the dual method M06-2X/6-311++G(df,p)//6-31+G(df,p) over the temperature range of 200–400 K.

## Graphical abstract

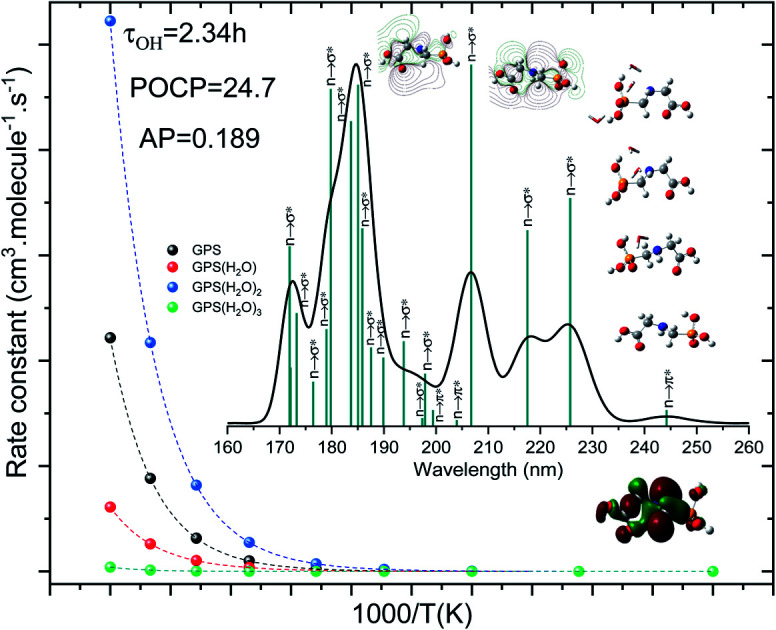

## 1 Introduction

The troposphere is an essential part of the environment as it plays a vital role in the circulation of important molecules (water, carbon, nitrogen, *etc.*) and in the detoxification of air.^1,2^ Agricultural industrialization within the last two centuries has been marked by increasing use of chemical products. Some of these compounds released into the troposphere have relatively long atmospheric lifetimes and hold some potential to cause adverse environmental effects like global warming, distortion of natural cycles and air pollution.^2–6^ The degree of harm they can cause depends on their atmospheric lifetimes and the troposphere's ability to get rid of them. There is a plethora of organic compounds in the troposphere, and glyphosate is one of them.^7,8^ Glyphosate of chemical name *N*-(phosphonomethyl) glycine with chemical formula (OH)_2_–PO–CH_2_–NH–CH_2_–COOH is shorten as GPS. It is an organophosphate, non-selective, broad-spectrum, agrochemical herbicide that is used to kill or suppress growth of grasses, forbs, vines, shrubs, and trees by inhibiting the synthesis of aromatic amino acids necessary for protein formation in susceptible plants.^7–9^ GPS is a colourless, odourless crystalline powder, and the most widely and intensively used herbicide across the world.^7,8^ The vapour pressure for GPS is very low, hence it is non-existent in atmosphere through volatilization^10^ as we would expect for other volatile organic compounds. Despite this, recent works conducted in agricultural areas show that GPS is observed in over 60% of air and rain samples with concentrations ranging from 0.01 to 9.1 ng m^−3^ in air samples and from 0.1 to 2.5 μg L^−1^ in rainwater samples.^8^ This is as a result of its intensive agricultural use. GPS in the atmosphere can be as a result of its movement into the air during spraying of roundup and/or wind erosion of contaminated soil particles which is transported in association with particulate matter (dust) and not as vapour. Detections of GPS in precipitation are more likely as it combines with dust particles being washed down with rain than to GPS dissolved in rain water.^11,12^

In the troposphere, O_3_, NO_=1–3_, and ˙OH radicals and Cl-atom are responsible for the oxidative degradation of many organic pollutants with ˙OH radical being the most oxidative free radical.^13^ Because of its oxidizing potential, degradation by ˙OH radical can act as an important possible sink in natural environments.^13^ Cl-atom is known to be an important oxidant in the tropospheric degradation of H-atom containing compounds,^14^ capable of perturbing tropospheric oxidative cycles normally controlled by the hydroxyl radical,^15^ and are predominantly found in marine region.^16^ So far, little or no attention has been given to the experimental and the theoretical studies of the rate constant and the atmospheric lifetime of the reactions involving GPS herbicide with the ˙OH radical or Cl-atom as compared to those of volatile organic compounds.^17,18^ Contrary to other volatile organic compounds, little information exists concerning the gas phase reactions of organophosphates in the troposphere.^19–23^ Haag *et al.*^24^ have evaluated rate constants for reaction of ˙OH radical with several drinking water contaminants of which hydrated GPS had a reported rate constant value of (3.1 ± 0.08) × 10^−13^ cm^3^ molecule^−1^ s^−1^ but, he reported that this rate constant may have been affected by complexation with iron.

Paying attention to UV-vis spectroscopy which involves electronic transitions in molecules, Scott *et al.*^25^ found by the mean of spectrophotometric technique that, the GPS in solution with varying pH from 2.83 to 10.01, absorbs in the window range 190–250 nm. The peak of the absorption band is located in the ultraviolet region,^25^ implying that the GPS remains colourless in different pH solution and at a wavelength of about 200 nm in the neutral region. Kumar *et al.*^26^ found that, the maximum peak stands around 204 and 208 nm from experiment and theory, respectively. For the experiment, they used Shimadzu-1800s UV-vis spectrometer (cubed 1 cm length) in aqueous medium at different pH (4, 7, and 10) maintained by using HCl and NaOH dilute solution. For theoretical method, the HF/6-311++G(d,p) method was used.

The concerns of the present work are: (i) to study the UV-vis spectra of GPS herbicide and its hydrates, GPS(H_2_O)_*n*=1–3_, (ii) to calculate the rate constant of the reaction of ˙OH radical towards GPS and its hydrates, and (iii) to estimate the atmospheric lifetimes and photochemical ozone creation potentials (POCP) of GPS and its hydrates from their sink by ˙OH radical from atmosphere, and the acidification potentials (AP).

The hydrates GPS(H_2_O)_*n*=1–3_ were made in order to mimic GPS suspended in the atmosphere in the presence of explicit water molecule, which results in wetted GPS. The atmospheric lifetime of a molecule is an essential parameter used to compute the global warming potential of a chemical compound.^27–29^ Oxidative degradation of organic compounds by free radicals can proceed through different reaction pathways. The rate constant of the oxidative degradation of GPS were determined through H-abstraction mechanism in the presence of ˙OH radical. This mechanism is suspected to happen by five plausible channels as presented in reactions (R1a)–(R1f).

R1a (OH)_2_–PO–CH_2_–NH–CH_2_–COOH + ˙OH → (OH)_2_–PO–CH_2_–NH–CH_2_–COO˙ + H_2_O

R1b (OH)_2_–PO–CH_2_–NH–CH_2_–COOH + ˙OH → (OH)_2_–PO–CH_2_–NH–CH˙–COOH + H_2_O

R1c (OH)_2_–PO–CH_2_–NH–CH_2_–COOH + ˙OH → (OH)_2_–PO–CH_2_–N˙–CH_2_–COOH + H_2_O

R1d (OH)_2_–PO–CH_2_–NH–CH_2_–COOH + ˙OH → (OH)_2_–PO–CH˙–NH–CH_2_–COOH + H_2_O

R1e (OH)_2_–PO–CH_2_–NH–CH_2_–COOH + ˙OH → (HO)O˙–PO–CH_2_–NH–CH_2_–COOH + H_2_O

R1f (OH)_2_–PO–CH_2_–NH–CH_2_–COOH + ˙OH → ˙O(HO)–PO–CH_2_–NH–CH_2_–COOH + H_2_O

The thermochemistry of these five pathways was assessed; the enthalpy and free energy of these reactions were calculated in order to predict the feasibility and spontaneity of the reaction channels. The bond dissociation enthalpy (BDE) of X–H (X = C, N, or O atom) was used to compare the heat required to break the X–H bonds in the GPS and its hydrates. The BDE was calculated at five sites as presented in the reactions (R2a)–(R2f).

R2a (OH)_2_–PO–CH_2_–NH–CH_2_–COOH → (OH)_2_–PO–CH_2_–NH–CH_2_–COO˙ + H˙

R2b (OH)_2_–PO–CH_2_–NH–CH_2_–COOH → (OH)_2_–PO–CH_2_–NH–CH˙–COOH + H˙

R2c (OH)_2_–PO–CH_2_–NH–CH_2_–COOH → (OH)_2_–PO–CH_2_–N˙–CH_2_–COOH + H˙

R2d (OH)_2_–PO–CH_2_–NH–CH_2_–COOH → (OH)_2_–PO–CH˙–NH–CH_2_–COOH + H˙

R2e (OH)_2_–PO–CH_2_–NH–CH_2_–COOH → (HO)O˙–PO–CH_2_–NH–CH_2_–COOH + H˙

R2f (OH)_2_–PO–CH_2_–NH–CH_2_–COOH → ˙O(HO)–PO–CH_2_–NH–CH_2_–COOH + H˙

Based on the scope of the present work, the BDE is the thermodynamic parameter that is used to determine the selectivity of the preferred channel.

The transition state theory (TST) in its thermodynamic formulation is the popular tool used for analysing rate constants of chemical reactions. Over the past two decades, significant progress has been made in developing methods for quantitative predictions of reaction rate constants based upon the dynamical formulation of TST.^30,31^ This theory was employed in this investigation to assess the rate constant of the reactions (R1a)–(R1e), since the reaction channel (R1f) is similar to that of (R1e); the low-energy structures of the products are similar in the pathways (R1e) and (R1f).

The present work was completed by the means of the density functional theory (DFT) and the time dependent variant (TD-DFT). The exchange–correlation functionals M06-2X and M06-L combined with the appropriate split valence basis sets were employed, respectively. This is in order to yield accurate results of structures, electronic energies, UV-vis spectra, vibrational frequencies, enthalpies and free energies of the species in the reaction processes.

## 2 Methodology

### 2.1 Computational details

Computations were carried out using the density functional theory (DFT) implemented in the Gaussian 09 suite package.^33^ The time dependent DFT (TD-DFT) and DFT schemes through the meta-hybrid exchange correlation functional M06-2X were employed to reach the specific objectives of the present work. This exchange–correlation functional was required based on the theoretical studies which have proven the excellent performance of this functional towards thermochemistry and kinetic studies.^14,32,34,35^ This functional was combined with the split valence basis sets of Pople *et al.*^36,37^ The potential energy surfaces (PESs) of GPS were attained through the rotation of dihedral angles using the M06-2X/6-31G(df) method. The equilibrium geometries and frequencies of GPS and its hydrates were carried out using the dual method M06-2X/6-311++G(df,p)//6-31+G(df). However, for the kinetics of the reactions of ˙OH radical towards GPS and its hydrates, the M06-2X/6-311++G(df,p)//6-31+G(df,p) method was assigned. This composite basis set produced excellent results regarding the rate constant of ˙OH radical towards greenhouse gases.^38–41^ The single point energy calculations were done without orbital symmetry constraints. The direct inversion in the iterative subspace (DIIS) procedure^42^ was used for geometry optimizations. The equilibrium geometries of the different species of this study were validated based on the real frequencies obtained, except for the transition state equilibrium where only one imaginary frequency should be observed. The TD-DFT was performed based on the Runge–Gross scheme^43^ at the M06-L/6-311++G(3df,3pd) level of theory, in order to have the UV-vis spectra of GPS and its hydrates.

### 2.2 UV-vis spectral generation

The UV-vis spectrum of chemical species is turned out by the list of wavelengths and oscillator strengths employing the Gaussian broadening function. This function is the molar extinction coefficient *ε*(*ω*) that quantifies the ability of chemical species to absorb light at a given wavelength *λ*.^44,45^ This is expressed as follows:1
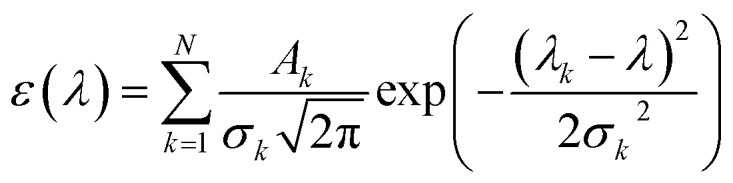
where *N* indicates the number of excited states. The subscript *k* refers to the *k*^th^ absorption peak in the spectrum, *λ*_*k*_ is the wavelength at the origin, *λ* is any given wavelength, and *σ*_*k*_ is the dispersion that is related to the full width at half-maximum (*Γ*) by 
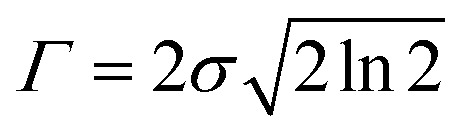
. *A*_*k*_ indicates the absorption intensity of each band including the oscillator strength *f*_*k*_ = 2*mω*_*k*_|*μ*_*k*_|^2^/3ℏ by the relation:2
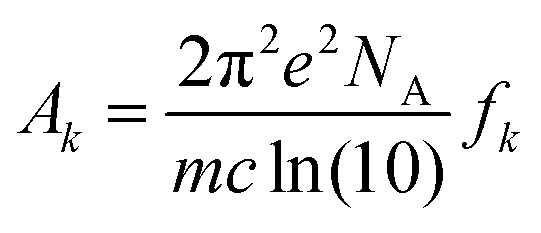
where *m* and *e* are the mass and electric charge of electron, respectively. *N*_A_ is the Avogadro's number, *c* is the speed of light, and *μ*_*k*_ is the transition dipole moment from ground state to *k*^th^ excited state.

### 2.3 Thermodynamics and kinetics of reactions

The H-abstraction mechanism presented in reactions (R2a)–(R2e) is characterized by the bond dissociation enthalpy (BDE)^46–52^ of all the X–H bonds of glyphosate molecule (C–H, O–H, and N–H). This is assessed using eqn (3).

3 BDE_298_ = *Δ*_f_*H*°(GPS–X˙) + *Δ*_f_*H*°(H˙) – *Δ*_f_*H*°(GPS–XH)

However, the H-atom transfer (HAT) from gaseous GPS to ˙OH radical is governed by the enthalpies and Gibbs free energies of reactions (R1a)–(R1e).^46–52^ These thermal parameters were calculated using the expression of eqn (4) and (5).

4 Δ*H*_r,298_ = *Δ*_f_*H*°(GPS–X˙) + *Δ*_f_*H*°(H_2_O) − *Δ*_f_*H*°(GPS–XH) − *Δ*_f_*H*°(˙OH)

5 Δ*G*_r,298_ = *Δ*_f_*G*°(GPS–X˙) + *Δ*_f_*G*°(H_2_O) − *Δ*_f_*G*°(GPS–X–H) − *Δ*_f_*G*°(˙OH)

where *Δ*_f_*H*°(Y) and *Δ*_f_*G*°(Y) are the enthalpy and Gibbs free energy of formation of species Y. The physical quantity *Δ*_f_*H*°(Y) is the sum of electronic energy, zero-point vibrational energy (ZPVE), and the thermal correction to enthalpy, while *Δ*_f_*G*°(Y) = *Δ*_f_*H*°(Y) + *RT*, where *R* represents the gas constant.

The rate constants of the reactions (R1a)–(R1e) were calculated based on the transition state theory (TST). This theory is the most popular issue for assessing rate constants of chemical processes. Over the past two decades, significant progress has been made in developing methods for quantitative predictions of rate constants based upon the dynamical formulation of TST. The conventional TST rate constant at temperature *T*^30,31,53,54^ for a bimolecular reaction reads as:6
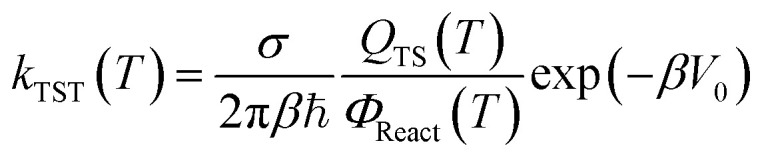
*β* = 1/*k*_B_*T*, *k*_B_ is the Boltzmann constant, ℏ is the reduced Planck constant, and *σ* denotes the number of indistinguishable ways the reactants may approach the activated complex regions. *Q*_TS_(*T*) is the partition function at transition state, and *Φ*_React_(*T*) = *Q*_A_*Q*_B_ is the total partition function of reactants A and B per unit volume at temperature *T*. *V*_0_ is the barrier height of the reaction. Another variant of TST is the vibrational transition state theory (VTST)^31,54–57^ in which, *Q*_TS_(*T*) and *V*_0_ varies with the reaction coordinate as *Q*_TS_(*T*,*s*) and *V*_0_(*s*). Readers are directed to ref. 30, 31 and 57 for more details. In VTST variant, the rate constant is expressed as the minimum of *k*_VTST_(*T*,*s*), that is 
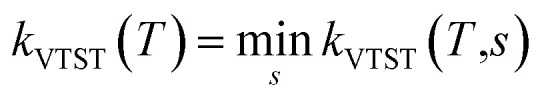
. This method was performed in the present work for the reaction of ˙OH radical towards GPS and its hydrates. In the total partition function of the reactants (*Φ*_React_(*T*) = *Q*_GPS_*Q*_OH_), the contribution of electronic partition function of ˙OH radical takes into account the splitting of the ground state Π^2^ into Π_1/2_^2^ and Π_3/2_^2^. The separation 139.7 cm^−1^ of these sub-states was used in the calculations.^31,34,38^ The electronic partition of ˙OH radical is then written as follows *Q*_elec_(OH) = 2 + 2 exp((139.7 cm^−1^)ℏ*c*/*k*_B_*T*), where ℏ and *c* are the Planck's constant and speed of light, respectively. It is worth mentioning that, the internal rotation modes of GPS and its hydrates were treated using the hindered rotor approximation,^58–60^ whereas the other vibrational frequencies were treated harmonically.

Considering the quantum tunnelling effects,^30,53,61^ the rate constant is multiplied by the quantum tunnelling factor *η*(*T*) as per eqn (7).

7 *k*(*T*) = *η*(*T*)*k*_VTST_(*T*)

The *η*(*T*) factor is defined as the ground state transmission coefficient at temperature *T*.^30,31^ This factor is quantitatively the ratio of the thermally averaged multidimensional semi-classical transmission probability to the thermally averaged classical transmission probability for scattering by the effective potential. In the present work, Skodje–Truhlar^55,57,62^ was used to approximate this factor rather than Wigner^63,64^ popular approximation. This is due to the fact that, *α* = 1/ℏ|*ω*^≠^| is not much greater than *β* = 1/*k*_B_*T*. This variational TST with the quantum tunneling effects was carried out using in-built FORTRAN 95 code.

## 3 Results and discussion

### 3.1 Structural study of molecular systems

To come out with the atmospheric implication of this study, the geometrical structures of glyphosate (GPS) and its hydrates was paramount challenges to deal with. The following sub-sections show the computational protocol used to yield the low-energy conformers of GPS and its hydrates.

#### 3.1.1 Structure of glyphosate molecule

**Fig. 1:**
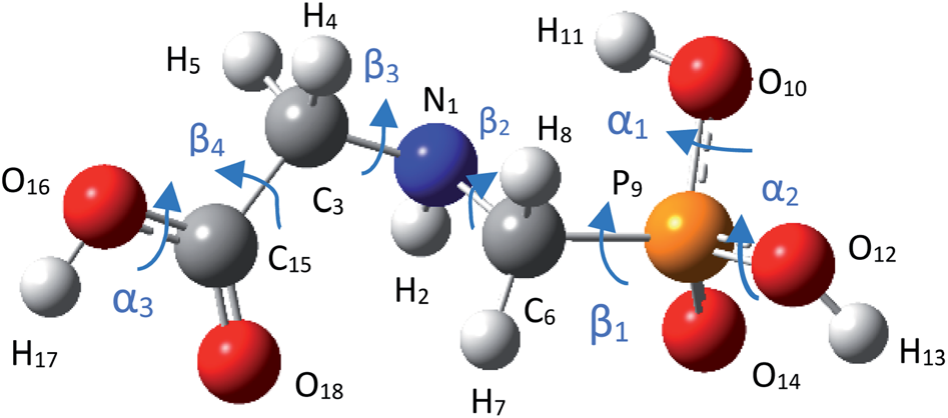
Equilibrium geometry of low energy GPS molecule.

**Fig. 2:**
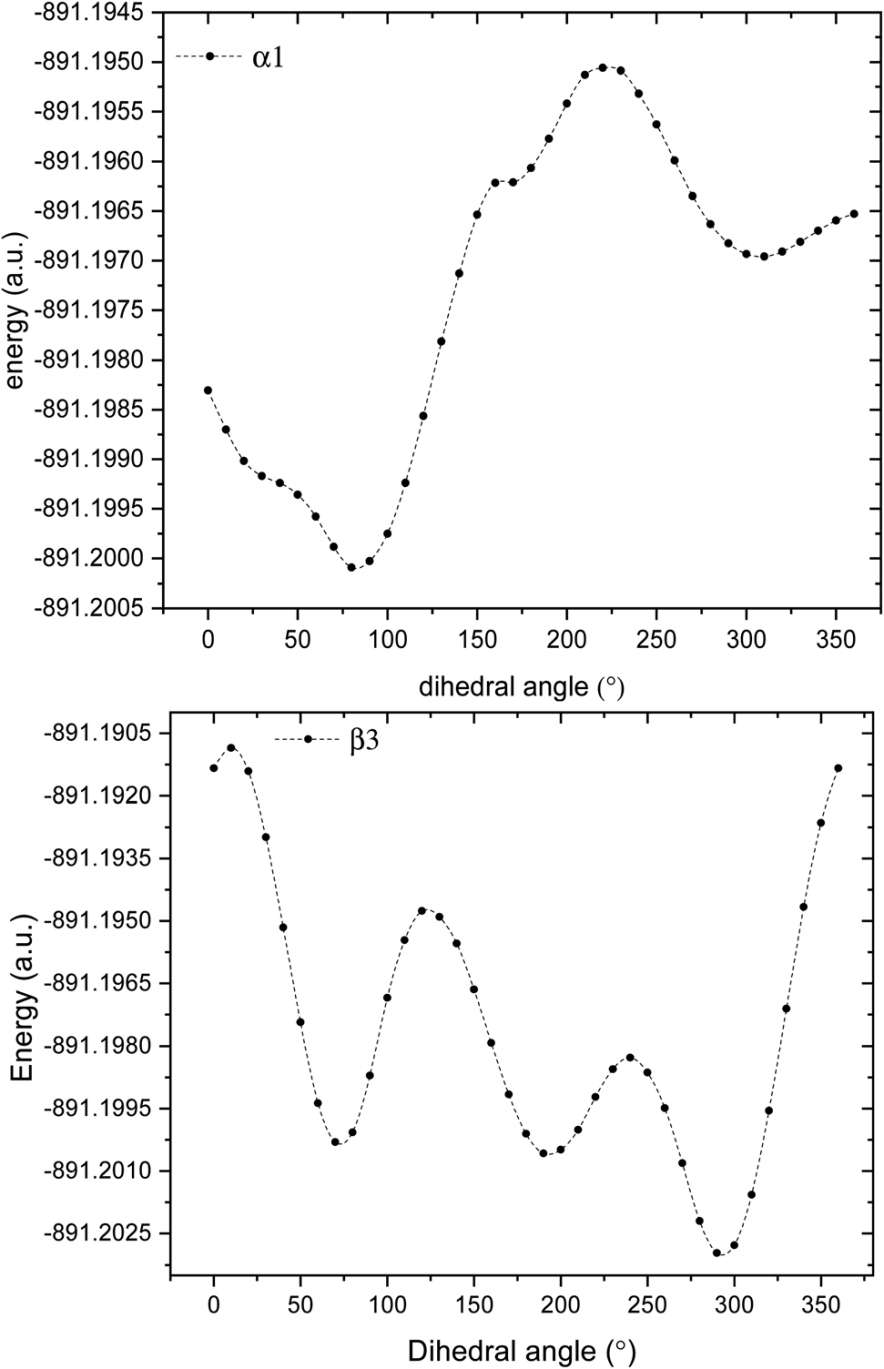
Potential energy surfaces of GPS molecule obtained by rotating the dihedral angles *α*_1_, and *β*_3_.

GPS molecule consists of two molecular fragments; methylphosphonic acid (–CH_2_–POOH–OH) and amino group of glycine (–NH–CH_2_–COOH) joined together through a bond angle centred at the N-atom given by C_3_–N_1_–C_6_ = 113.9°. In the search of the low-energy structure of glyphosate, the rotations of angle 360° in steps of 10° of the functional groups were completed. These angles were labelled *α*_1_, *α*_2_, *β*_1_, *β*_2_, *β*_3_, *β*_4_, and *α*_3_ as shown in Fig. 1. The rotations of respective angles *α*_1_, *α*_2_, and *α*_3_ are those which carry the –OH group. They correspond to the dihedrals C_6_–P_9_–O_10_–H_11_, C_6_–P_9_–O_12_–H_13_, and C_3_–C_15_–O_16_–H_17_, respectively. The rotations of respective angles *β*_1_, *β*_2_, *β*_3_, and *β*_4_ are those which carry the other functional groups. These correspond to the dihedrals N_1_–C_6_–P_9_–O_12_, C_3_–N_1_–C_6_–P_9_, C_15_–C_3_–N_1_–C_6_, and O_16_–C_15_–C_3_–N_1_. The potential energy surfaces (PESs) from the rotations of respective angles *α*_1_ and *β*_3_ were plotted and presented in Fig. 2. The other PESs are compiled in Fig. 1s as supplementary data (SD).† The conformer of the lowest point of each PES was used to achieve the other PES as presented in the order of rotation of angles *α*_1_, *α*_2_, *β*_1_, *β*_2_, *β*_3_, *β*_4_, and *α*_3_, respectively. Therefore, the minimum from each rotation led to the low-energy of GPS as per Fig. 1.

The geometric parameters such as bond lengths and bond angles of the low-energy GPS structure are reported in Table 1 and compared with the computed and experimental results of Kumar *et al.*^26^ It is worth recalling that, the computed results of Kumar *et al.* have been succeeded using the HF/6-311++G(d,p) method. The root means square error (RMSE) of our results and the computed ones from Kumar *et al.*^26^ yielded 0.109 and 0.120 Å for bond length, respectively, and 5.68 and 7.07° for bond angles, respectively. It follows that, our findings are closer to the experimental results than those of computed results from Kumar *et al.*^26^ Moreover, the bond length N_1_–C_6_ and bond angle C_3_–N_1_–C_6_ that join the glycine and the methylphosphonic acid groups at the N-atom is given by 1.468 Å and 114.5°, respectively. They are deviated from experimental results by 0.011 Å and 0.1°, respectively.

**Table 1:** Bond lengths and bond angles of the low-energy GPS structure. The experimental results are reported with the computed results of Kumar *et al.*^26^ completed at HF/6-311++G(d,p) level of theory

Bond length (Å)				Bond angle (°)			
Parameter	This work	Computed^26^	Exp.^26^	Parameter	This work	Computed^26^	Exp.^26^
C_15_–O_18_	1.202	1.197	1.201	H_17_–O_16_–C_15_	108.5	112.7	114.8
C_15_–O_16_	1.343	1.357	1.308	O_16_–C_15_–O_18_	123.5	122.8	125.5
C_15_–C_3_	1.521	1.506	1.507	O_16_–C_15_–C_3_	125.1	110.3	122.3
C_3_–N_1_	1.447	1.456	1.491	O_18_–C_15_–C_3_	111.5	127.0	124.2
N_1_–C_6_	1.468	1.458	1.479	H_4_–C_3_–H_5_	106.7	108.4	116.2
C_6_–P_9_	1.817	1.815	1.816	H_4_–C_3_–C_15_	108.6	108.1	—
P_9_–O_10_	1.600	1.640	1.568	H_4_–C_3_–N_1_	100.0	113.4	—
P_9_–O_12_	1.601	1.630	1.568	C_3_–N_1_–C_6_	114.5	115.8	114.4
P_9_–O_14_	1.474	1.537	1.501	N_1_–C_6_–P_9_	105.1	106.0	112.2
O_16_–H_17_	0.970	0.968	1.038	H_11_–O_10_–P_9_	111.1	118.6	105.9
C_3_–H_4_	1.095	1.091	1.057	H_13_–O_12_–P_9_	112.3	121.1	105.9
C_3_–H_5_	1.096	1.079	0.957	C_6_–P_9_–O_14_	114.3	117.4	109.9
N_1_–H_2_	1.018	1.001	0.698	C_6_–P_9_–O_10_	105.2	106.4	106
C_6_–H_7_	1.096	1.087	0.936	C_6_–P_9_–O_12_	102.8	100.0	104.2
C_6_–H_8_	1.095	1.081	0.903	O_14_–P_9_–O_10_	115.3	112.6	111.4
O_10_–H_11_	0.973	0.966	1.021	O_14_–P_9_–O_12_	116.7	116.9	118.2
O_12_–H_13_	0.966	0.965	1.018	O_10_–P_9_–O_12_	100.9	101.7	105.7

#### 3.1.2 Structure of wetted glyphosate

**Fig. 3:**
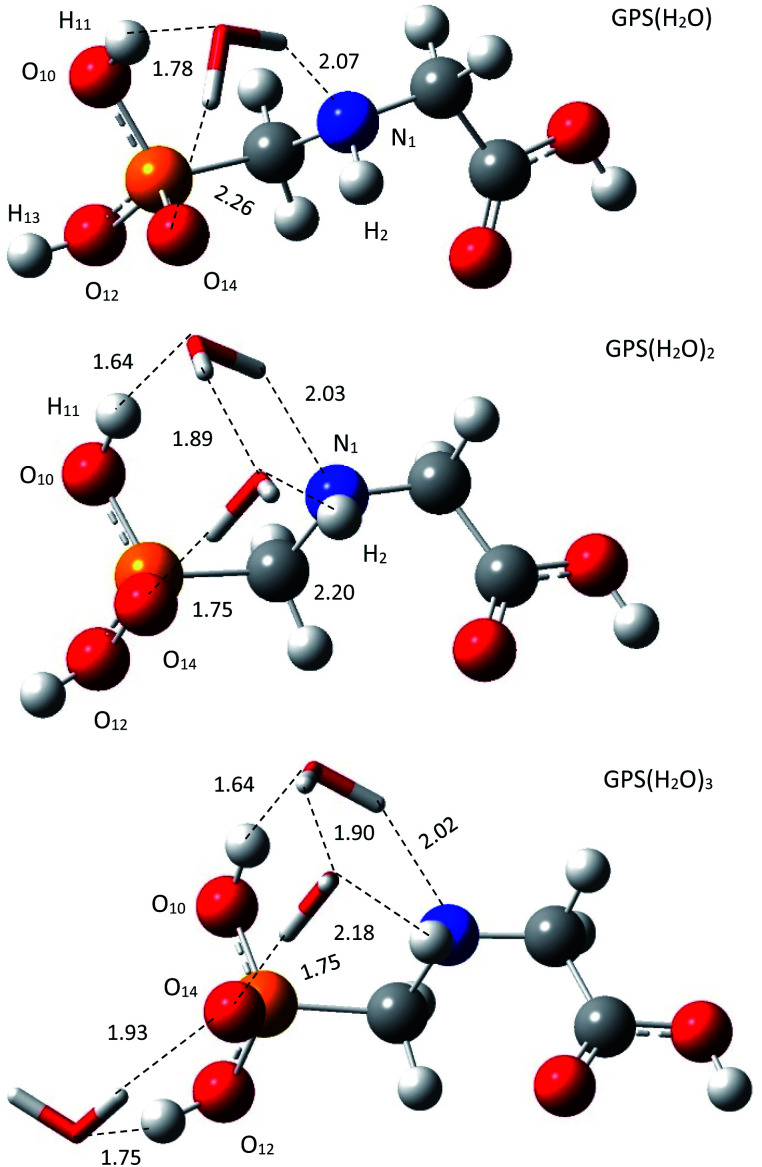
Low-energy equilibrium structures of GPS(H_2_O), GPS(H_2_O)_2_, and GPS(H_2_O)_3_ obtained using M06-2X/6-31+G(3df).

**Fig. 4:**
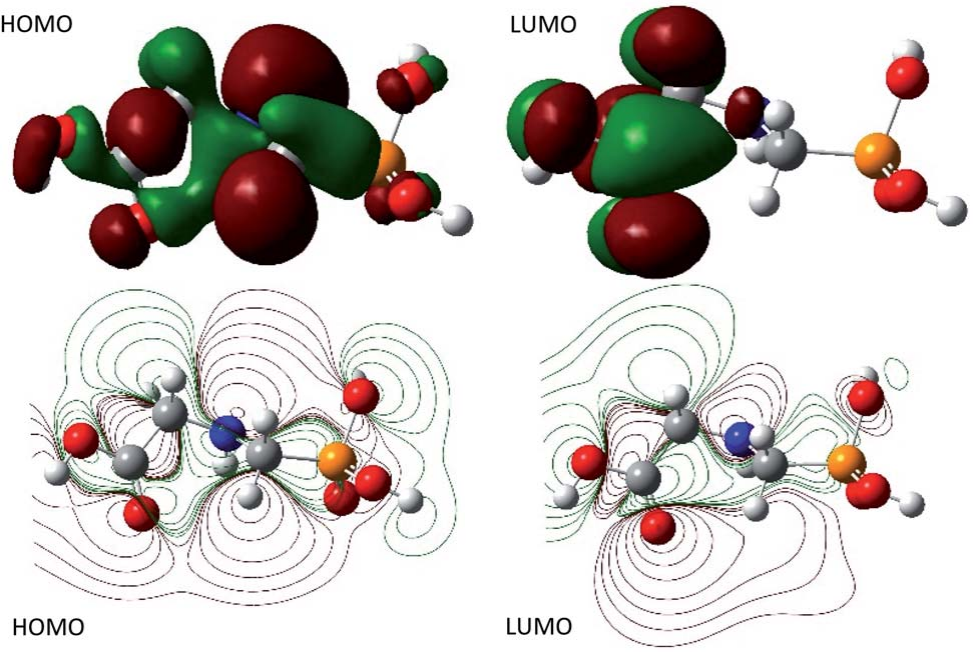
HOMO and LUMO surfaces of gaseous GPS.

The escaped GPS in the lower atmosphere can form a network with water vapour through hydrogen bond (HB). In our framework, we made the one-, two-, and three-hydrated GPS complexes (GPS(H_2_O)_*n*=1–3_) in gas phase. The low-energy structure of GPS molecule was used to form these complexes. All the conformers of each complex of cluster size *n* = 1–3 were optimized and compiled in Fig. 2s–4s as SD.† The low-energy complex GPS(H_2_O) served to build the conformers of GPS(H_2_O)_2_. The latter served to make those of GPS(H_2_O)_3_. The best candidates for the present work were selected based on the lowest-energy amongst the conformers of different size. The selected complexes are depicted in Fig. 3 along with the lengths of HBs. The single water molecule of the complex GPS(H_2_O) prefers to be connected to O_10_–H bond, O_14_ and N_1_ atoms and models an irregular tetrahedron. The second added water molecule for the complex GPS(H_2_O)_2_ forms a network with the first one and the prism scheme of three faces raise. The third added water molecule for the complex GPS(H_2_O)_3_ prefers to connect with the O_12_–H bond and O_14_ atom and designs an irregular triangle.

### 3.2 Global reactivity, degradation indexes, and heat capacities

The global reactivity indexes of GPS and its hydrates such as the magnitude of the energies of the highest occupied molecular orbital (*E*_HOMO_) and the lowest unoccupied molecular orbital (*E*_LUMO_), and the hardness defined as the energy gap between HOMO and LUMO levels, are reported in Table 2. They were carried out at the M06-L/6-311++G(3df,3pd) level of theory. The ionization potential (IP) and electron affinity (EA) are given by the *E*_HOMO_ and *E*_LUMO_ values, respectively. It comes out that, the IP value (6.02 eV) of GPS is slightly impacted by the presence of water molecules. The EAs of GPS and its hydrates are around unity (1 eV). The effects of water molecules are almost negligible. The EAs are low (approaching to 0 eV) and the LUMOs are near the limit of ionization of the molecular systems. Therefore, GPS and its wetted forms are nucleophile. Based on the hardness values, the order of reactivity is as follows: GPS (5.02 eV) > GPS(H_2_O)_2_ (5.20 eV) ≈ GPS(H_2_O)_3_ (5.21 eV) > GPS(H_2_O) (5.26 eV). Thus, the wetted GPS are less reactive than gaseous one. Globally, the GPS and its hydrates are less reactive since their hardness is less than 1.30 eV.^65,66^

**Table 2:** Magnitude of HOMO and LUMO energies (eV), hardness HOMO–LUMO (eV), dipole moment (Debye), polarizability (au), heat capacity (kJ mol^−1^ K^−1^), and entropy (kJ mol^−1^ K^−1^)

	HOMO	LUMO	Hardness	Dipole	Polarizability	Heat capacity	Entropy
GPS	6.02	0.99	5.02	2.88	85.62	0.177	0.447
GPS(H_2_O)	6.47	1.22	5.26	3.31	94.76	0.210	0.490
GPS(H_2_O)_2_	6.30	1.11	5.20	2.74	104.64	0.247	0.542
GPS(H_2_O)_3_	6.30	1.09	5.21	2.63	114.64	0.282	0.589

Moreover, the natural bond orbital (NBO) analysis was assessed and the NBO charges are reported in Table 1s as SD.† It turns out that, HOMO and LUMO are n and π* characters, respectively (Fig. 4). The nitrogen and all the oxygen atoms in the structure of GPS and its hydrates are carriers of negative charges, while the phosphorus atom is a hole charge. Thus, the P-atom transferred its charges to N- and O-atoms which kept their electron lone pair. Therefore, we can understand why the HOMO is n character. On the other side, the dipole moment and the average polarizability are reported in Table 2. We can see that, the order of degradation of GPS and its hydrates from the dipole moment analysis is stated as follows: GPS(H_2_O) > GPS > GPS(H_2_O)_2_ > GPS(H_2_O)_3_. Thus, in humid air, GPS reduces its dipole moment and resist to degradation. This is in discordance with the average polarizability analysis where, the order of degradation follows as GPS(H_2_O)_3_ > GPS(H_2_O)_2_ > GPS(H_2_O) > GPS. In this case the humid air favours the degradation of GPS.

Furthermore, we have heat capacities and entropies of GPS and its hydrates reported in Table 2. They were assessed at M06-2X/6-31+G(df) level of theory. It is noticeable that, both thermal quantities increase in the present of water molecules. Thus, humid air has an ability to make GPS as a reservoir of heat. This prove that, GPS in the troposphere can result incontestably in climate change.

### 3.3 UV-vis spectroscopies of glyphosate and its hydrates

In order to identify GPS in the atmosphere, the TD-DFT calculations were first performed at M06-2X/6-311++G(3df,3pd) level of theory. The spectroscopic parameters such as transition states along with their respective wavelengths, and oscillator strengths were compiled in Table 2s as SD.† The results failed while comparing with experiment. However, Kumar *et al.*^26^ performed the calculation using HF/6-311++G(d,p) and found that, the wavelength at the maximum absorption yielded 208.0 nm. This is approximately closer to the experimental results (204.0 nm). Our calculations yielded 162.15 nm far from the observed one. To improve this result, we did a diagnostic based on the fact that HF/6-311++G(d,p) yielded good results, thus GPS molecule is a single reference system. Therefore, a pure DFT can be a good deal for the spectroscopy of GPS and its hydrates. Then we selected the pure functional M06-L of the same family of Menesota University to perform TD-DFT calculations. The new results are compiled in Table 3s of SD.† The obtained results gave satisfaction (206.74 nm).

**Fig. 5:**
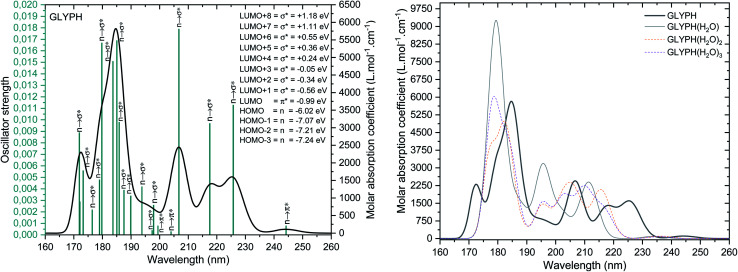
UV-vis spectra of GPS its hydrates. The full width at half maximum was taken at 0.1 eV.

The UV-vis spectra of the GPS and its hydrates are presented in Fig. 5. The main peak at 206.74 nm has a weak absorption coefficient value. This is due to the nearby peaks in which the overall Gaussian function accumulates the individual ones. The effects of water molecules impact seriously on the spectrum of GPS. The blue shifts of peaks are noticeable along with the enhancement of the absorption peaks (hyperchromic effects). However, it is seen that, the spectra are localized in the UV window range 160–260 nm. This advises that, GPS and its hydrates are colourless.

A careful attention was done on the different transitions reported in Table 3s (SD).† As indicated in the above Section (3.2), the occupancies of orbitals from NBO analysis and the HOMO–LUMO contours and surfaces have allowed to state that, all the HOMO – *x* (*x* is an integer) involved in the UV-vis spectra are of n character. LUMO is of π* character and the other LUMO + *x* (*x* > 1) are of σ* characters. The contours and surfaces of Fig. 4 and 5s† give evidence on these different characters. The energy of each molecular orbital (MO) is presented along with the type of the bond in Fig. 5 on the left panel. We remark that, all the HOMOs are naturally bound states, but only LUMO + *x* (*x* = 0–3) are bound states. The others with *x* > 3 are free states. Thus all the transitions with low oscillator strengths involve the LUMO + *x* (*x* > 3) indicating that, such events appear to ionize GPS and precipitate it in the process of degradation.

### 3.4 Thermodynamics of reactions

In the presence of ˙OH radical, the reaction process of GPS can advent from six different issues as presented in the reactions (R1a)–(R1e). The bond dissociation mechanisms from the five channels are stated at equations (R2a)–(R2e). The enthalpies of these reactions (BDE) at 298.15 K are reported in Table 3. This thermal parameter advises on the amount of heat required to dissociate the C–H, N–H, and O–H bonds of GPS. The results are compared to the computational BDEs of other molecules as quercetin (282.8 kJ mol^−1^ at B3LYP/6-31++G*),^57^ caffeic acid phenethyl ester (312.0 kJ mol^−1^ at B3LYP/6-31+G**),^46,47^ 3,4-dihydroxyphenylpyruvic acid (304 kJ mol^−1^ at B3LYP/6-311++G**),^49^*p*-phenylenediamine (340 kJ mol^−1^ at B3LYP/6-31++G**)^52^ and tetracyano-*p*-phenylenediamine (381 kJ mol^−1^ at B3LYP/6-31++G**),^52^ and the experimental BDE of 3,4-dihydroxycinnamic acid (285 kJ mol^−1^ is in excellent agreement with theory at B3LYP/6-31++G** fit).^50^ In these molecules, the BDE were obtained from the O–H bonds except for *p*-phenylenediamine and tetracyano-*p*-phenylenediamine where the BDE was calculated from the N–H bonds. The computational results were assessed using the B3LYP functional combined with the Pople's basis sets 6-31++G*, 6-31+G**, 6-31++G**. It is seen that, the computational BDEs from the N–H bond are greater than those obtained from O–H bonds. This is consistent with what observed in GPS and its hydrates. However, the BDEs of GPS increase in the following order: BDE (site C_6_) < BDE (site O_16_) < BDE (site C_3_) < BDE (site N_1_) < BDE (site O_10_) = BDE (site O_12_). For wetted GPS, it follows the order: BDE (site C_3_) < BDE (site O_16_) < BDE (site C_6_) < BDE (site N_1_) < BDE (site O_12_) = BDE (site O_10_). Therefore, the most favourable site of radical attacks is C_6_ for gaseous GPS and C_3_ for wetted GPS. In addition, the H-atom at site O_10_ and O_12_ are strongly attached, they require a great amount of heat for dissociation. The BDEs from the exothermic paths ((R1a)–(R1d)) are reliable with the molecules used for comparison, while the BDE from the endothermic path (R1e) is out of order. The GPS radical obtained after the dissociation of O_16_–H bond involved in the carboxylic group, is thermodynamically unstable. It dissociates in turns into carbon dioxide CO_2_ and 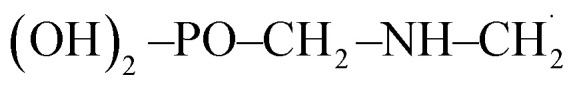 radical. This mechanism is written as per equation (R3).R3
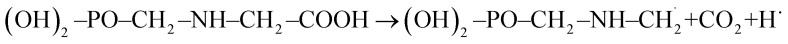


**Table 3:** BDE (kJ mol^−1^) of C–H, N–H and O–H in the GPS and its hydrates, and the enthalpy Δ*H*° (kJ mol^−1^) and Gibbs free energy Δ*G*°(kJ mol^−1^) of the processes of H-atom transfer (HAT) to ˙OH at 298.15 K

Reaction	(R1a)	(R1b)	(R1c)	(R1d)	(R1e)
**BDE**_**298**_					
GPS	311.5	322.1	375.3	292.0	461.4
GPS(H_2_O)	314.7	296.6	370.1	327.6	461.8
GPS(H_2_O)_2_	313.0	293.5	376.6	337.6	471.2
GPS(H_2_O)_3_	312.6	275.3	375.7	339.1	491.3

**Δ*H***_**r,298**_ **/HAT**					
GPS	−140.3	−129.7	−76.5	−159.8	+9.6
GPS(H_2_O)	−137.1	−155.2	−81.7	−124.2	+10.0
GPS(H_2_O)_2_	−138.8	−158.3	−75.2	−114.2	+19.4
GPS(H_2_O)_3_	−139.2	−176.5	−76.1	−112.7	+39.6

**Δ*G***_**r,298**_ **/HAT**					
GPS	−158.2	−135.8	−82.0	−159.5	+6.9
GPS(H_2_O)	−155.4	−164.2	−87.8	−134.6	+4.9
GPS(H_2_O)_2_	−157.4	−154.8	−79.6	−119.5	+16.8
GPS(H_2_O)_3_	−158.4	−176.2	−86.5	−120.4	+35.8

In the calculation of the BDE of O_16_–H, the enthalpy of the overall system 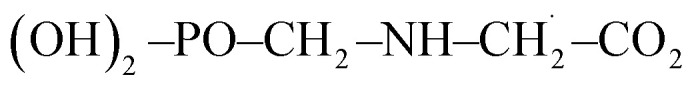 was considered as it takes into account the interaction between individual subsystems.

On the other side, the enthalpies Δ*H*_r,298_ and Gibbs free energies Δ*G*_r,298_ of reactions involving GPS (and its hydrates) and ˙OH radical were estimated at 298.15 K (Table 3) in order to predict the feasibility and spontaneity of the reaction channels. It turns out that, the reaction channels (R1a)–(R1d) are exothermic, while the channel (R1e) is endothermic. This is due to the nucleophilic character of the molecular systems at the level of P-atom presented in Section 3.2. In fact, in the GPS molecule, the charge hole is made on P-atom and the electron lone pairs take place on the O-atoms and reinforce the enthalpy of the O–H bonds. It is noticeable that, this fact is correlated with the BDE and explains why channel (R2e) has the greatest value of BDE. Dealing with Δ*G*_r,298_, it comes out that, the reaction channels (R1a)–(R1d) are spontaneous, while the channel (R1e) is non-spontaneous. Thus, we can conclude that, the reaction channel (R1e) is unfeasible; therefore, an attention was paid only on the reaction channels (R1a)–(R1d) in the section devoted to kinetics of chemical reactions.

Moreover, in the presence of ˙OH radical, the reaction process (R3) yields three different products such as H_2_O, CO_2_ and 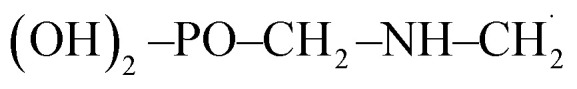. As in the case of BDE calculation, the enthalpy and Gibbs free energy of formation of the overall system 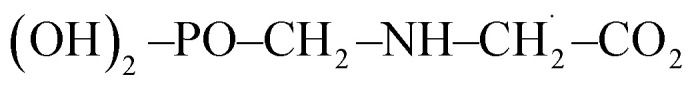 was considered in the calculations of Δ*H*_r,298_ and Δ*G*_r,298_. In view of the production of CO_2_ by the channel (R1a) and the feasibility of the reaction, one can assert that GPS is a reservoir of CO_2_ in the lower atmosphere.

### 3.5 Kinetics of reactions

**Fig. 6:**
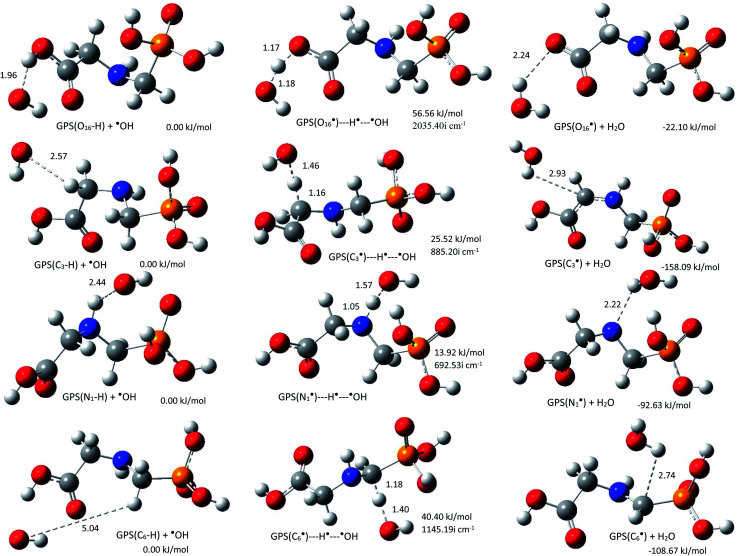
Reaction complex (RC), transition state (TS), and product complex (PC) for each reaction pathway of ˙OH radical towards GPS. The relative electronic energy of each step of the process are presented in kJ mol^−1^. The internuclear lengths along the reaction coordinates and the imaginary frequency at TS are given in Å and cm^−1^, respectively.

**Fig. 7:**
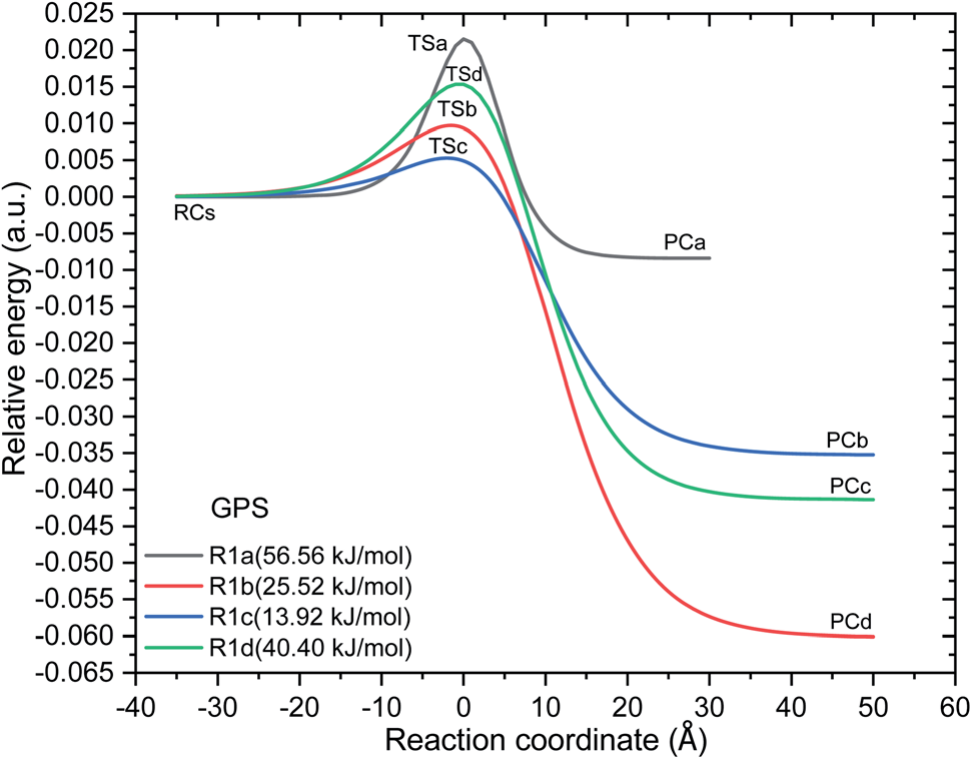
Minimum energy potentials of the reaction of ˙OH towards gas phase GPS. With the values of electronic energies at reaction complex (RC), transition state (TS), and product complex (PC), these potentials were interpolated by the Eckart unsymmetrical function.^30,31^ The barrier height from the reaction complex of each reaction path are presented in parenthesis (at the bottom of well).

**Fig. 8:**
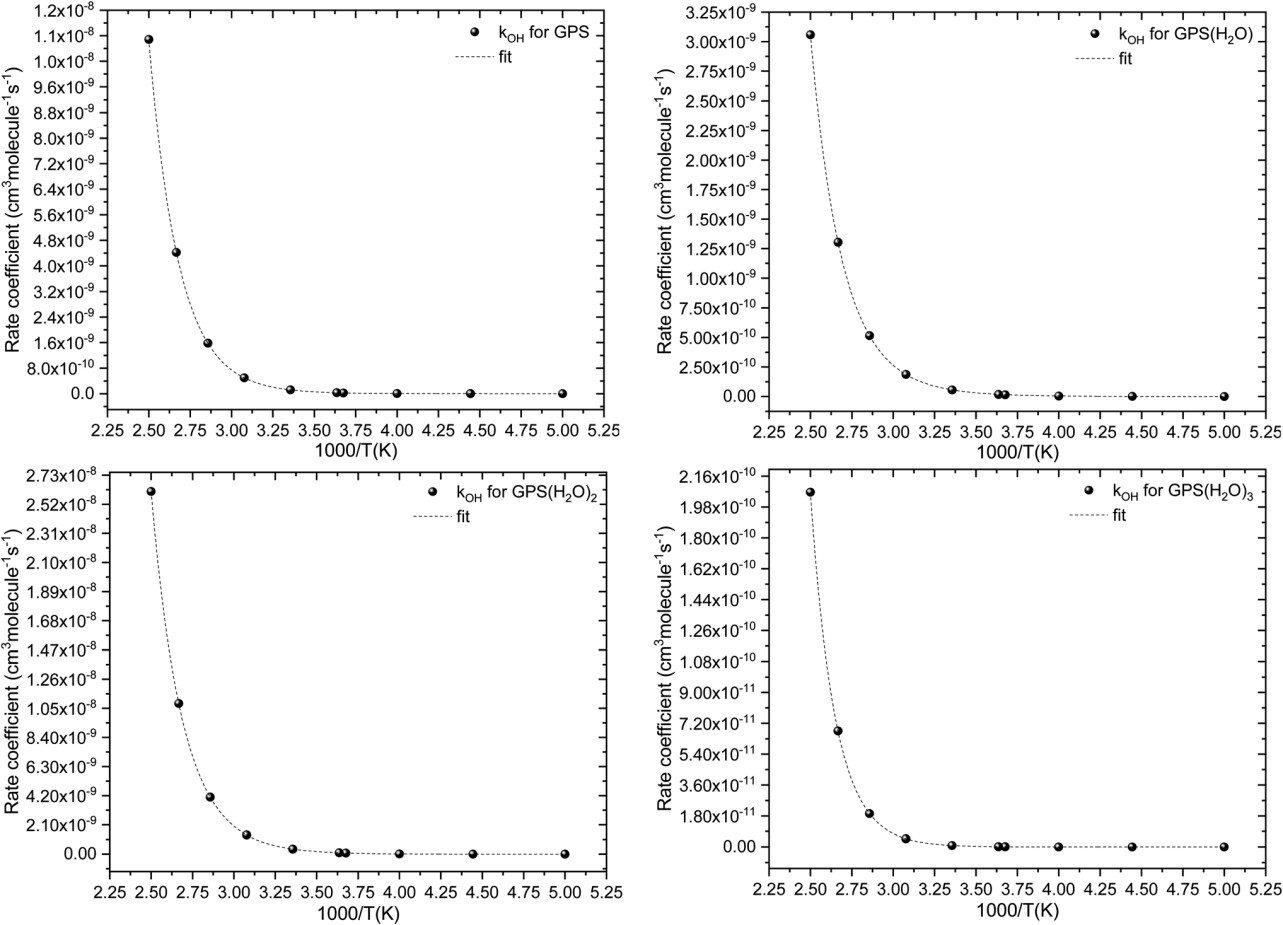
Temperature-dependent rate constant of the reaction between GPS (and its hydrates) with the ˙OH radical over the temperature range 200–400 K.

The rate constants *k*_OH_ of the reactions of ˙OH radical towards GPS and its hydrates were evaluated at all the thermodynamically feasible hydrogen sites. This includes the first four channels ((R1a)–(R1d)). The equilibrium geometries at reaction complex (RC), transition state (TS), and product complex (PC) for each pathway in gas phase are presented in Fig. 6 along with their relative energy (relative to RC). The other equilibrium geometries involving GPS hydrates are presented in Fig. 6s–8s as SD.† The stabilization of RCs and TSs for GPS and GPS(H_2_O) were examined by the basis set superposition error (BSSE)^67^ corrected binding energies using the M06-2X/6-311++G(df,p) method. They were calculated from two fragments; ˙GPS/˙GPS(H_2_O) and ˙OH radicals for RCs and three fragments; ˙GPS/˙GPS(H_2_O, H˙, and ˙OH radicals for TSs. The results are compiled in Table 5s (SD†). As shown, the difference between binding energies of RCs and TSs are within 10^−3^ au, which can be ignored in the rate constant calculation.

As shown along the reaction coordinate, RC is first formed and as the reaction progresses, the intermediate (TS) is appeared and finally PC takes place. The three states along the minimum energy path (MEP) are stabilized by the interactions between GPS and ˙OH. The results of single point energies (SPEs), Gibbs free energies (GFEs), and energy barrier heights (EBHs) are reported in Table 4 for GPS and its hydrates GPS(H_2_O)_*n*=1–3_ in each reaction path. The distances between the participants of the reactions are provided in Fig. 6 for GPS and for GPS hydrates in Fig. 6s–8s (SD†). All the species involved in the reaction paths (R1a)–(R1d), are verified by real vibrational positive frequencies, while TSs are confirmed by one imaginary frequency (IF) in the list of frequencies. The imaginary frequencies are depicted in Fig. 6 and 6s–8s† for each reaction path. The magnitude of these imaginary frequencies follows the order IF(R1a) > IF(R1d) > IF(R1b) > IF(R1c) for GPS. The larger the IF, the shorter is the width of the MEP. This is noticeable on the MEP presented in Fig. 7, where the narrowest and the largest potential barriers are along the (R1a) and (R1d), respectively. This behaviour is also observed for GPS hydrates (Fig. 9s as SD†). The relative energies of TSs are the energy barrier height (EBH) of the reaction path, which follows the order (R1a) > (R1d) > (R1b) > (R1c) for GPS. For GPS hydrates, one can read (R1a) > (R1b) > (R1d) > (R1c) for GPS(H_2_O), (R1a) > (R1d) > (R1b) > (R1c) for GPS(H_2_O)_2_ and GPS(H_2_O)_3_. Thus, this order for GPS with water dimer and water trimer agrees with the one established for GPS in gas phase. Upon addition of water molecule, the EBH decreases in the reaction path (R1a), while the other paths show a disharmony. The EBHs of all the paths are further lowered or broken by water continuum. The continuum was assessed using the solvation model based on the quantum mechanical charge density of a solute molecule interacting with a continuum (SMD).^68^ In fact, the calculations show that the mechanism of H-abstractions from GPS in water continuum are spontaneous since the reaction complexes (RCs) are unstable. Once a RC is formed, the reaction moves to the product complex (PC). The structures of PCs in water continuum are shown in Fig. 10s (SD†).

**Table 4:** Single point energies (SPEs) (au), Gibbs free energies (GFEs) (au), and energy barrier heights (EBHs) (kJ mol^−1^) of RC, TS, and PC along the reaction path of GPS and its hydrates at 298 K from the processes of H-atom abstraction by ˙OH radicals. The zero-point vibrational energies (ZPVEs) are included in the GFEs. RC, TS, and PC are the reaction complex, transition state, and product complex, respectively

Molecule	Reaction	(R1a)			(R1b)			(R1c)			(R1d)		
		SPE	GFE	EBH	SPE	GFE	EBH	SPE	GFE	EBH	SPE	GFE	EBH
GPS	RC	−967.17466	−966.92263	56.56	−967.16952	−966.92189	25.52	−967.17838	−966.92835	13.92	−967.17688	−966.92742	40.40
	TS	−967.15312	−966.91616		−967.15980	−966.91937		−967.17307	−966.92291		−967.16150	−966.91992	
	PC	−967.18307	−966.93491		−967.22973	−966.98097		−967.21365	−966.96231		−967.21827	−966.97014	
GPS(H_2_O)	RC	−1043.6225	−1043.3225	53.01	−1043.6194	−1043.3196	27.43	−1043.6253	−1043.3265	8.83	−1043.6162	−1043.3179	23.88
	TS	−1043.6023	−1043.3144		−1043.6090	−1043.3160		−1043.6220	−1043.3240		−1043.6071	−1043.3155	
	PC	−1043.6401	−1043.3454		−1043.6899	−1043.3856		−1043.6627	−1043.3621		−1043.6672	−1043.3662	
GPS(H_2_O)_2_	RC	−1120.0703	−1119.7220	52.22	−1120.0689	−1119.7213	23.00	−1120.0737	−1119.7227	31.39	−1120.0640	−1119.7160	23.11
	TS	−1120.0504	−1119.7158		−1120.0602	−1119.7160		−1120.0617	−1119.7155		−1120.0552	−1119.7163	
	PC	−1120.0823	−1119.7380		−1120.1348	−1119.7848		−1120.1066	−1119.7574		−1120.1152	−1119.7675	
GPS(H_2_O)_3_	RC	−1196.5153	−1196.1191	52.14	−1196.5119	−1196.1167	34.71	−1196.5180	−1196.1211	14.89	−1196.5153	−1196.1191	39.73
	TS	−1196.4954	−1196.1121		−1196.4987	−1196.1116		−1196.5123	−1196.1143		−1196.5001	−1196.1136	
	PC	−1196.5487	−1196.1460		−1196.5793	−1196.1789		−1196.5506	−1196.1544		−1196.5590	−1196.1651	

However, as mentioned in Section 3.4, the channel (R1a) produces the carbon dioxide immediately after hydrogen abstraction. We then imposed the constraint to freeze the bond length C_3_–C_15_ of GPS and its hydrates. This was done in this particular case in order to apply the vibrational transition state theory (VTST). Elsewhere, the relative energies of product complexes are negative for all the different paths. This reveals that, all the reactions are exergonic. It is seen that, the barrier height of the channel (R1a) is the highest one for GPS and its hydrates. However, the width of the potential barrier depends of the value of the range parameter (*L*), which in turn depends on the magnitude of the imaginary frequencies. Thus, the lower value of *L* produces very large width (Fig. 9s†) as in (R1c) for GPS(H_2_O)_3_. This brings significant impact on the tunnelling effect.

The calculated values of rate constant (298 K) are reported in Table 5 along with the branching ratio of each pathway. As mentioned in Section 2.3, the internal rotation modes of GPS and its hydrates were used in place of vibrational modes using the hindered rotor approximation.^58–60^ The details are provided in Table 6s as SD.† The results show that, the dominant reaction is (R1a) with the highest value of branching ratio. The exception is seen for the hydrate GPS(H_2_O), where the highest branching ratio appeared at channel (R1c). The discrepancy with the barrier height comes from the narrow and large widths. (R1a) is kinetically more favourable for its narrow barrier (small width). The available experimental result is *k*_OH_ = (3.1 ± 0.08) × 10^−13^ cm^3^ per molecules per s^−1^ obtained in water continuum.^24^ This result is affected by the complexation with iron. Nevertheless, our findings with the maximum size of water cluster (*n* = 3) fit the experimental results at 78.71%. The remaining percentage can be corrected by supplementing explicit water molecules to the hydrate GPS(H_2_O)_3_.

**Table 5:** Rate constant (cm^3^ per molecule per s) along with branching ratio (%) for each reaction path of GPS and its hydrates at 298 K from the processes of H-atom abstraction by ˙OH radicals. The total rate constant is given by *k*_OH_ = *k*^a^_OH_ + 2*k*^b^_OH_ + *k*^c^_OH_ + 2*k*^d^_OH_

Reaction	(R1a)	(R1b)	(R1c)	(R1d)	Total
Rate constant	*k* ^a^_OH_	*k* ^b^_OH_	*k* ^c^_OH_	*k* ^d^_OH_	*k*_OH_
GPS	1.14 × 10^−10^	1.74 × 10^−12^	9.07 × 10^−13^	8.83 × 10^−14^	1.19 × 10^−10^
GPS(H_2_O)	7.56 × 10^−12^	1.85 × 10^−14^	2.96 × 10^−11^	9.22 × 10^−12^	5.56 × 10^−11^
GPS(H_2_O)_2_	2.27 × 10^−10^	4.09 × 10^−15^	7.79 × 10^−14^	6.76 × 10^−11^	3.62 × 10^−10^
GPS(H_2_O)_3_	5.20 × 10^−13^	8.91 × 10^−15^	5.10 × 10^−14^	1.33 × 10^−13^	8.56 × 10^−13^
GPS(H_2_O)_∞_					
Branching ratio	*k* ^a^_OH_/*k*_OH_	*k* ^b^_OH_/*k*_OH_	*k* ^c^_OH_/*k*_OH_	*k* ^d^_OH_/*k*_OH_	
GPS	96.15	1.47	0.76	0.08	—
GPS(H_2_O)	13.60	0.033	53.20	16.60	—
GPS(H_2_O)_2_	62.60	0.001	0.022	18.70	—
GPS(H_2_O)_3_	60.80	1.04	5.96	15.60	—

The fit of the total rate constant over the temperature range 200–400 K was completed for GPS and its hydrates (Fig. 8). The data are compiled in Table 4s (SD†). The fit yielded the non-Arrhenius equation of the form *k*^env^_OH_(*T*) = exp(*c* + *bT*^−1^ + *aT*^−2^), where *a*, *b* and *c* are real constants of the fit, and *T* the temperature in K. The superscript ‘env’ indicates the environment of GPS which can be gaseous state or explicit water. The equations of the rate constant as a function of temperature are given as per eqn (8)–(11).8
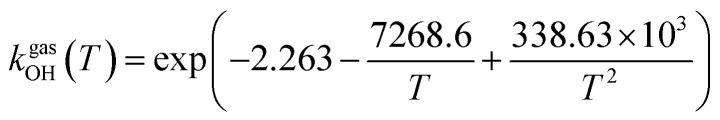
9
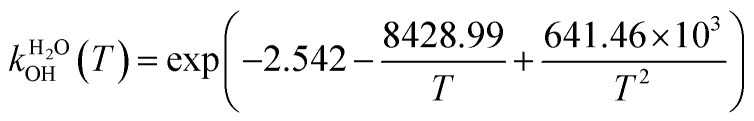
10
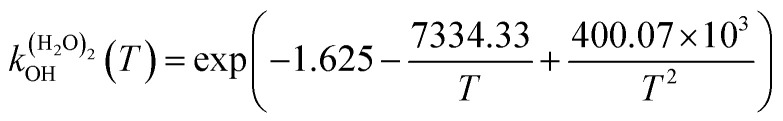
11
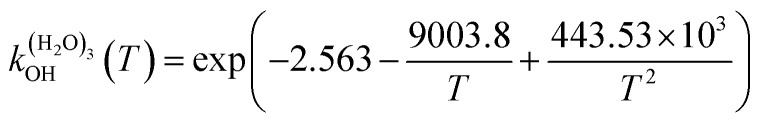


These equations were obtained with a coefficient of determination equal to unity.

## 4 Atmospheric implication

### 4.1 Atmospheric lifetime

With the rate constants of the reactions of ˙OH radical towards GPS and its hydrates, the atmospheric lifetime *τ*_OH_ of GPS and its hydrates were estimated as the inverse of the product of rate constant and the concentration of ˙OH radical in the troposphere.^69^ This is expressed as per eqn (12).12
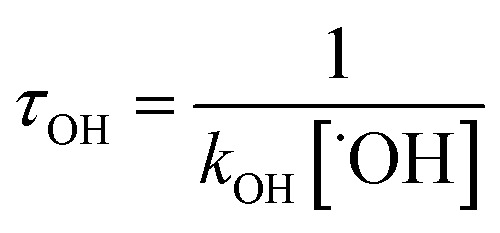
where *k*_OH_ represents the rate constant of the reaction of ˙OH radical with GPS and its hydrates. The quantity [˙OH] = 1.0 × 10^6^ molecule cm^−3^ is the global average atmospheric concentration of the ˙OH radicals.^70–72^ In fact, other oxidants as O_3_, ˙NO_*x*=1–3_ radicals, and Cl atom also contribute to the degradation of the gas in the troposphere.^70–72,75,76^ However, they are less reactive compared to ˙OH radical, which is known as the ‘atmospheric detergent’. This advises the use of ˙OH radical in the determination of atmospheric lifetime.

The results of atmospheric lifetime are reported in Table 6. They are of the order of hours and follow the order GPS(H_2_O)_2_ < GPS < GPS(H_2_O) < GPS(H_2_O)_3_. Thus, GPS and its hydrates are washed out from the troposphere once its release. However, the growth of the size of these hydrates may form aerosols and increase the atmospheric lifetime. However, GPS and its hydrates are not atmospherically well-mixed as they are short-live compounds. Therefore, they cannot be assigned to a unique atmospheric lifetime.^77^ The reported results represent the global atmospheric lifetime average. The local lifetime could significantly vary with the seasons, the physical and chemical conditions of atmosphere, and the location of emission.

**Table 6:** Atmospheric lifetime (hours), photochemical ozone creation potential (POCP), and acidification potential

	Atmospheric lifetime (hours)	POCP	Acidification potential
GPS	2.34	24.7	0.189
GPS(H_2_O)	4.99	23.0	0.171
GPS(H_2_O)_2_	0.77	20.0	0.156
GPS(H_2_O)_3_	324.49(13.52 days)	32.7	0.144

### 4.2 Photochemical ozone creation potential

Photochemical ozone creation potential (POCP) has been suggested to quantify the ability of a gas to create ozone in the troposphere. In fact, the emitted gas reacts with the oxidants (˙OH, ˙NO_3_, Cl, *etc.*) in the troposphere, which yields the corresponding peroxy/alkoxy radicals. Further, these peroxy/alkoxy radicals react with ˙NO to form ˙NO_2_, which in turn regenerates ˙NO along with the ozone molecule in the presence of light and oxygen. The procedure to estimate the POCP proposed by Jenkin (1998)^74,75^ was recently improved^78^ and the new estimation procedure is written as per eqn (13).

13 POCP_X_ = (*A* × *γ*_s_ × *R* × *S* × *F*) + *P* + *R*_O_3__ − *Q*)

where the subscript X stands for the molecular system GPS(H_2_O)_*n*=0–3_. The quantities *A*, *γ*_s_, *R*, and *S* are core parameters used for any compound, while *F*, *P*, *R*_O_3__ and *Q* are the parameters used for specific compound. These can take the default values of one (1) for *F* and zero (0) for *P*, *R*_O_3__ and *Q*. In North West European conditions, the parameter *A* = 100 is a multiplier. *γ*_s_ is the ozone formation index obtained from the structure of molecule X. it is expressed as per eqn (14).14
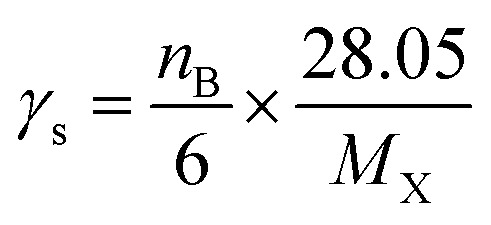
where *n*_B_ indicates the total number of reactive bonds (C–C, N–C, P–C, C–H, N–H, and O–H) in the molecule, *M*_X_ denotes the molecular weight of molecule X. *R* is a reactivity element that is related to the ˙OH reactivity of X. It takes the following from *R* = 1 − (*Bγ*_R_ + 1)^−1^. The parameters *B* = 4.0 under North West European conditions and *γ*_R_ is the ozone formation index obtained from the reactivity of molecule X. It is expressed as follows.15
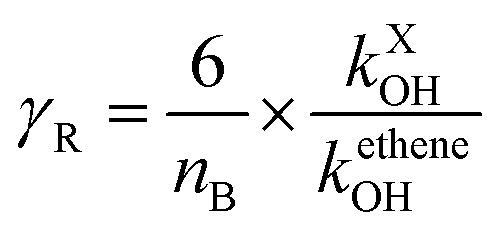
*k*^X^_OH_ is the rate constant for the reaction of molecule X with ˙OH radical at 298 K and 1 atm of air, and *k*^ethene^_OH_ is the rate constant for the reaction of ethene with ˙OH radical at 298 K and 1 atm of air. *k*^ethene^_OH_ was found by Jenkin^74–76^ to be 8.64 × 10^−12^ cm^3^ per molecule per s. The parameter *S* is related to the size of the molecule X and takes the form *S* = (1 − *α*) × exp(−*C*·*n*_c_^*β*^) + *α*, where *n*_*c*_ is the number of carbon of the gas molecule X. under North West European conditions, the values of *α*, *C*, and *β* are 0.56, 0.0038, and 2.7, respectively. The default values were assigned to the other parameters. As such, for GPS and its hydrates, the difference between their POCP is seen from their structure and reactivity indexes.

The results of the estimated POCPs are reported in Table 6. It comes out that, the gaseous GPS and its hydrates are not efficient ozone producer, as their POCPs values are low. It is 24.7 for GPS and decreases with the water monomer (23.0) and water dimer (20.0). The POCP of water trimer is 32.7 larger than the other ones including gaseous GPS. It lies on the range of the ozone formation potential of alkane.^73,79,80^

### 4.3 Acidification potential

Acid rain is one of the environmental concerns today, it impacts negatively on plants and animals on the earth's surface. When the substances containing the atoms N, F, Cl and S, get emitted into the atmosphere, they form the acid species such as HNO_3_, HF, HCl, and H_2_SO_4_ which contributes naturally to the acid rain. In accordance with the above, the acidification potential (AP) is the number of acid equivalent potentials (H^+^) per unit mass of a given compound X with respect to the number of H^+^ per unit mass of the reference compound SO_2_. It is thus a parameter to measure the ability of an emitted compound to contribute towards acid rain with respect to SO_2_ in the local environment. It is expressed as per eqn (16).^76,80^16
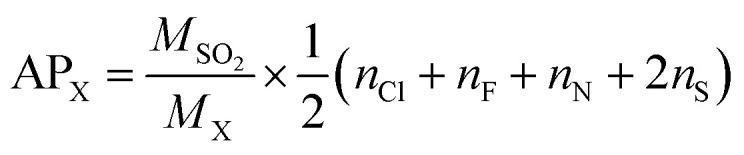
where *M*_SO_2__ and *M*_X_ represent the molecular weights of SO_2_ and molecule X = (GPS(H_2_O)_*n*=0–3_), respectively. The quantities *n*_Cl_, *n*_F_, *n*_N_, and *n*_S_ are the number of Cl, F, N, and S atoms, respectively, in the molecular system X. The estimated results are compiled in Table 6. It turns out that, the AP of GPS is 0.189 and decreases upon addition of water molecules. These values are much lesser than the AP of sulfur dioxide which is equal to unity. Therefore, the capacity of acidifying the precipitation by GPS and its hydrates are negligible as well as their atmospheric decomposition products.

## 5 Conclusion

The present paper deals with the UV-vis spectra and atmospheric implication of glyphosate (GPS) and its hydrates (GPS(H_2_O)_*n*=1–3_). Accordingly, the equilibrium structures of GPS and its hydrates were carried out using the M06-2X/6-31+G(df) method. The results of bond lengths and bond angles agree strongly with the experiment. The heat capacities and entropies of each GPS and its hydrates were evaluated. These thermal parameters indicate the degree of molecules to produce heat in the environment. As the values of these parameters increase for hydrates, then we come to assert that, humid air has an ability to make GPS as a reservoir of heat. The atmospheric lifetimes of GPS and its hydrates were then evaluated through their removal from troposphere by ˙OH radical. The rate constant of the reactions of ˙OH towards GPS and its hydrates were calculated by the means of vibrational transition state theory (VTST). First of all, the thermochemistry of these reactions were evaluated using the dual level M06-2X/6-311++G(df,p)//6-31+G(df). Five plausible reaction paths were identified and labelled (R1a)–(R1e). They are hydrogen abstraction from carboxylic –COOH, –NH, and –CH groups. The results of BDEs of the C–H, N–H, and O–H bonds reveal that, O–H bonds connected to P-atom require more heat for dissociation. The most thermodynamically feasible reaction is (R1d). Carbon dioxide (CO_2_) has appeared in the path (R1a) for GPS and its hydrates. This leads to assert that GPS is a potential carbon dioxide creator. All the reaction channels in the case of GPS proceed by the formation of pre-reaction complexes. The barrier height of the H-abstraction from the –COOH (path (R1a)) is the highest with the narrowest width. The branching ratio of this path is more consistent than the other pathways, indicating that path (R1a) is kinetically more labile. This situation is also observed in the case of hydrates except for GPS(H_2_O), where path (R1c) is kinetically more labile. The rate constant was fitted and expressed in non-Arrhenius model equation *k*^env^_OH_(*T*) = exp(*c* + *bT*^−1^ + *aT*^−2^) over the temperature range 200–400 K, where *a*, *b*, and *c* are real constants. The atmospheric lifetime was estimated from the rate constant along with the photochemical ozone creation potential (POCP). It turns out that, GPS and its hydrates are short-live compounds as their atmospheric lifetime are of the order of hours. GPS and its hydrates are inefficient ozone producers. The acidification potentials of GPS and its hydrates show negligible impact in the acidification of rain. The UV-visible spectra of GPS and its hydrates were carried out at M06-L/6-311++G(3df,3pd) level of theory. The results show that, the transitions from ground to excited states are dominated by n → σ* and n → π*. The spectra are localized in the window range 160–260 nm, which indicates that, the GPS and its hydrates are colourless. These findings are in excellent accordance with the experiment. The analysis of degradation indices showed that, humid air favours the degradation of GPS.

## Conflicts of interest

There are no conflicts to declare.

## Supplementary Material

RA-011-D1RA01591E-s001
