# Supplementary material for: Insight of UV-vis spectra and atmospheric implication for the reaction of ˙OH radical towards glyphosate herbicide and its hydrates
Source: RSC Adv. 2021 May 4;11(27):16404–18. doi: 10.1039/d1ra01591e (PMC9030808; doi:10.1039/d1ra01591e)
Supplement: RA-011-D1RA01591E-s001 [file RA-011-D1RA01591E-s001.pdf]

This journal is © The Royal Society of Chemistry 2021

Appendix A.

Supplementary data

1. Structures of glyphosate and its hydrates

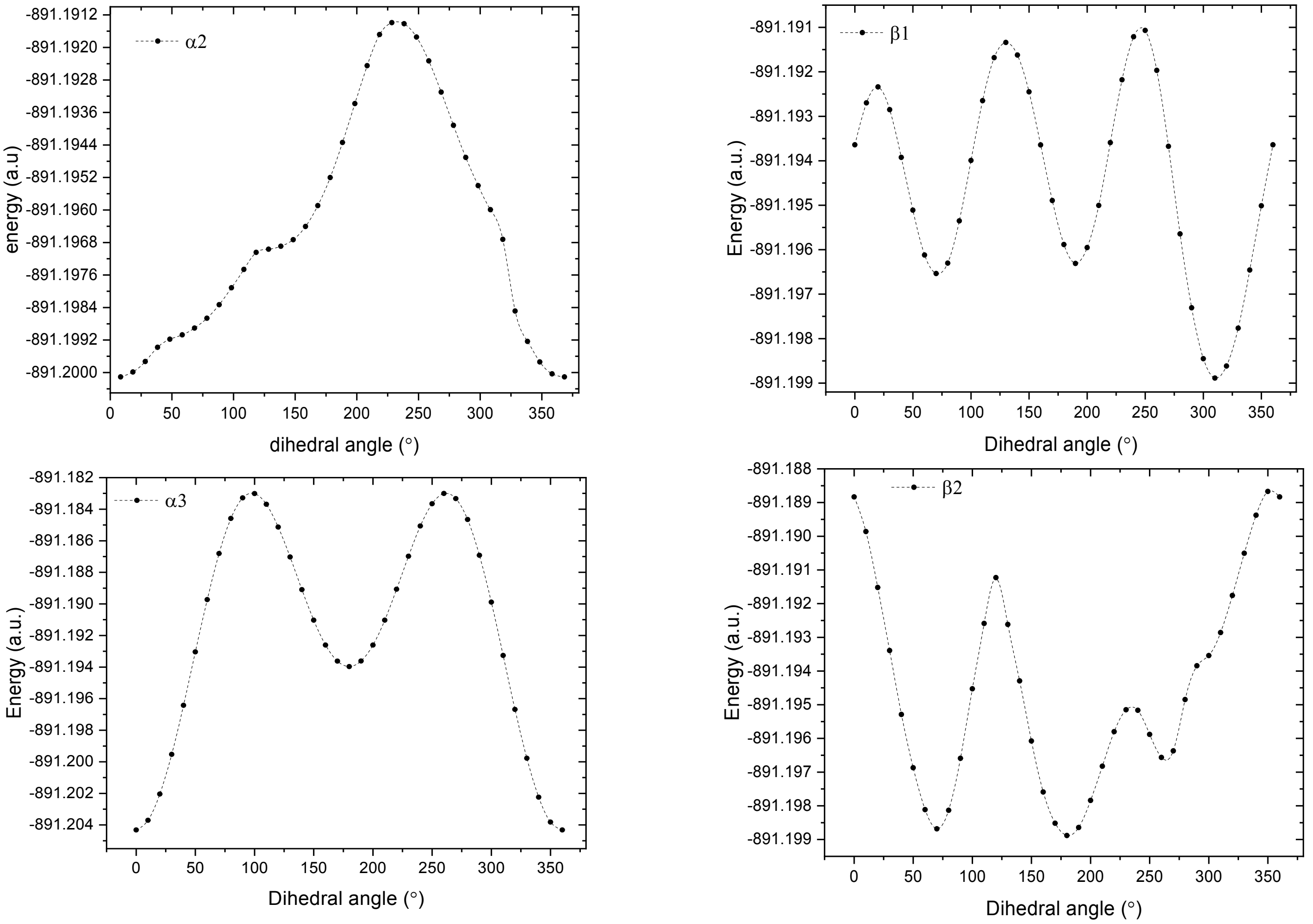

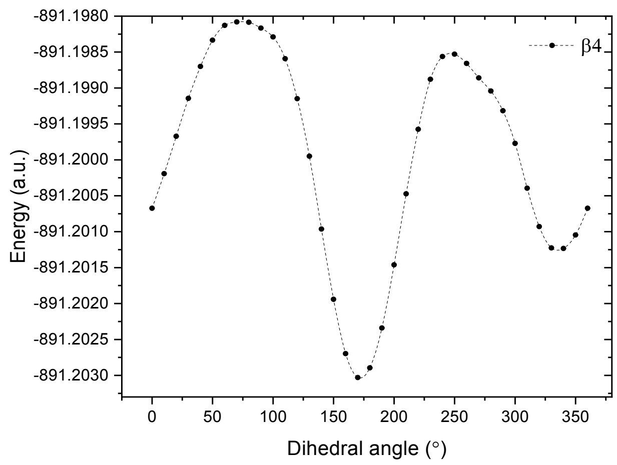

Fig. 1s. Potential energy surfaces of glyphosate (GPS) obtained from rotating the dihedral angles α_2, α_3, β_1, β_2, and β_4.

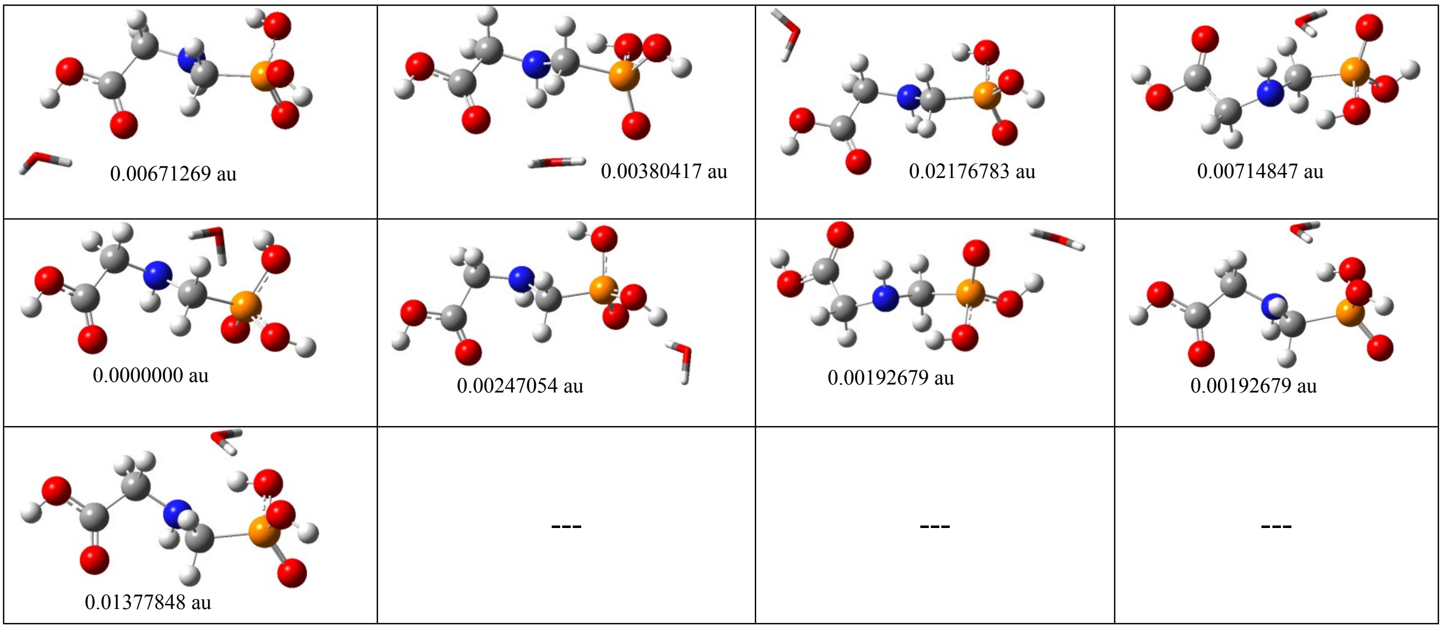

Fig. 2s. Equilibrium geometries of the possible conformers of GPS(H_2O) with their corresponding ground state electronic energy.

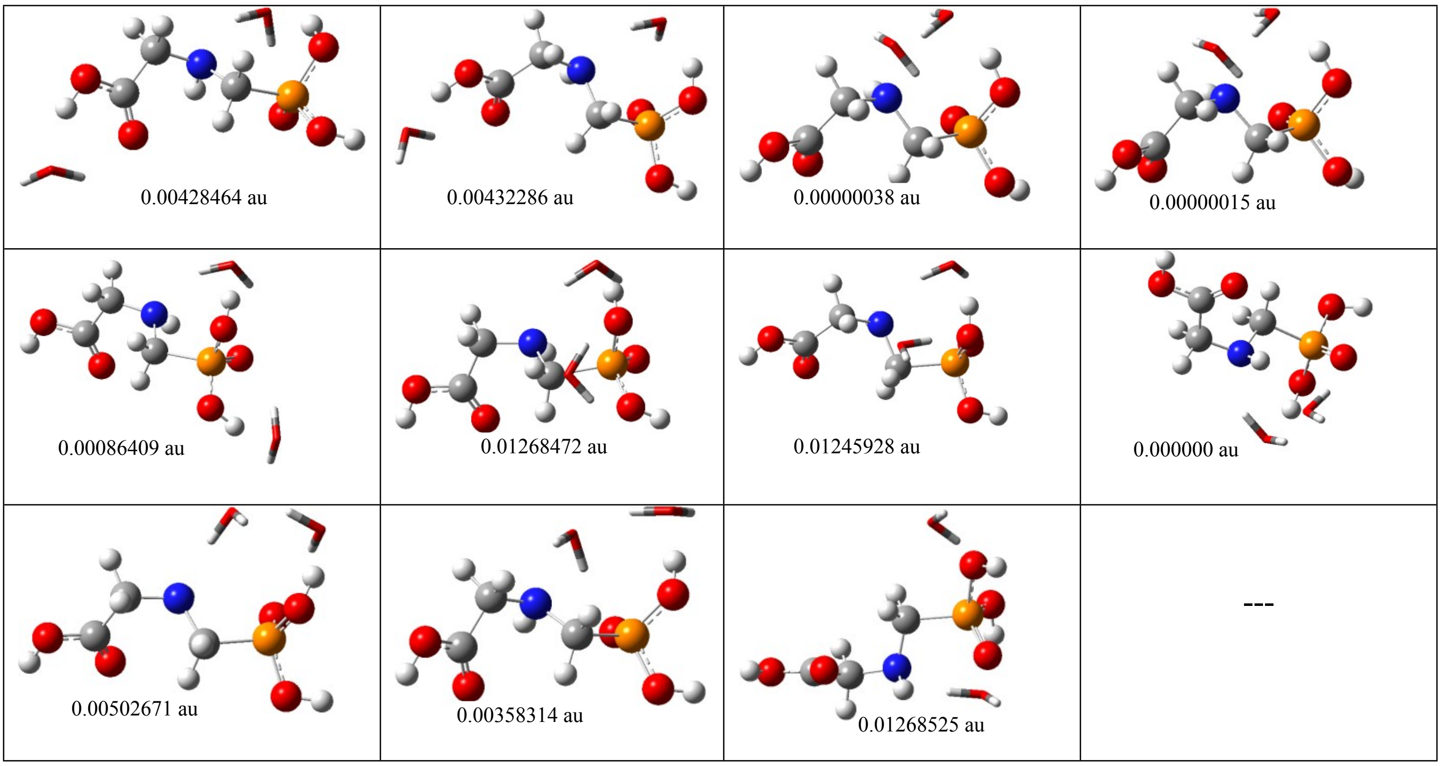

Fig. 3s. Equilibrium geometries of the possible conformers of GPS$(H_2O)_2$ with their corresponding ground state electronic energy.

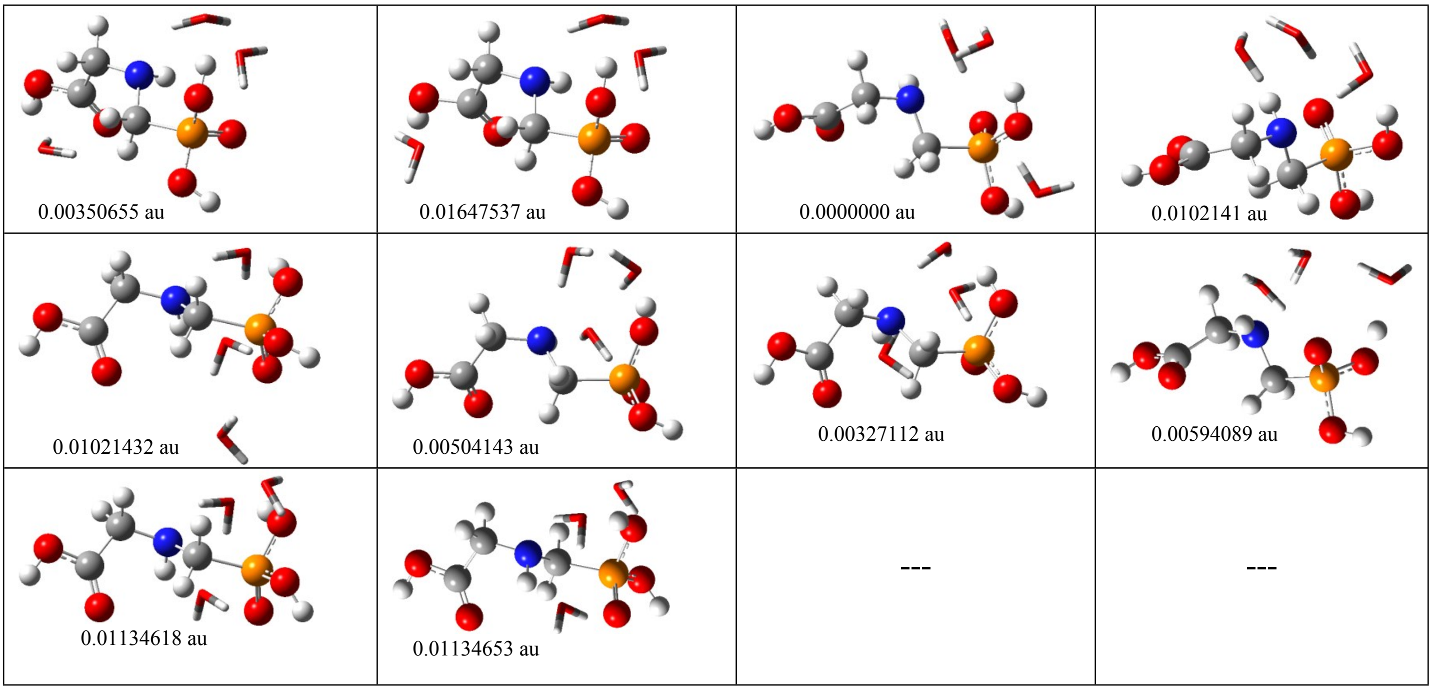

Fig. 4s. Equilibrium geometries of the possible conformers of GPS(H_2O)$_3$ with their corresponding ground state electronic energy.

Table 1s

Natural bond orbital charge (NBO) of glyphosate (GPS) and its hydrates in electron charge unit (e). The charges on water molecules are also reported.

Parameter	GPS	GPS(H_2O)	GPS(H_2O)$_2$	GPS(H_2O)$_3$
C_3	-0.371	-0.430	-0.371	-0.370
C_6	-0.683	-0.667	-0.684	-0.686
C_{15}	+0.816	+0.449	+0.818	+0.818
N_1	-0.745	-0.829	-0.769	-0.769
O_{10}	-1.055	-0.736	-1.080	-1.077
O_{12}	-1.050	-0.711	-1.047	-1.069
O_{14}	-1.127	-0.630	-1.176	-1.217
O_{16}	-0.740	-0.554	-0.738	-0.737
O_{18}	-0.617	-0.418	-0.615	-0.615
P_9	+2.450	+1.230	+2.482	+2.506
H_2	+0.430	+0.485	+0.446	+0.445
H_4	+0.266	+0.265	+0.268	+0.268
H_5	+0.278	+0.264	+0.280	+0.279
H_7	+0.267	+0.277	+0.270	+0.266
H_8	+0.268	+0.259	+0.270	+0.269
H_{11}	+0.546	+0.615	+0.564	+0.563
H_{13}	+0.545	+0.541	+0.546	+0.568
(H_2O)	---	+0.033	---	---
2(H_2O)	---	---	+0.011	---
3(H_2O)	---	---	---	+0.033

2. UV-vis spectroscopy of glyphosate and its hydrates

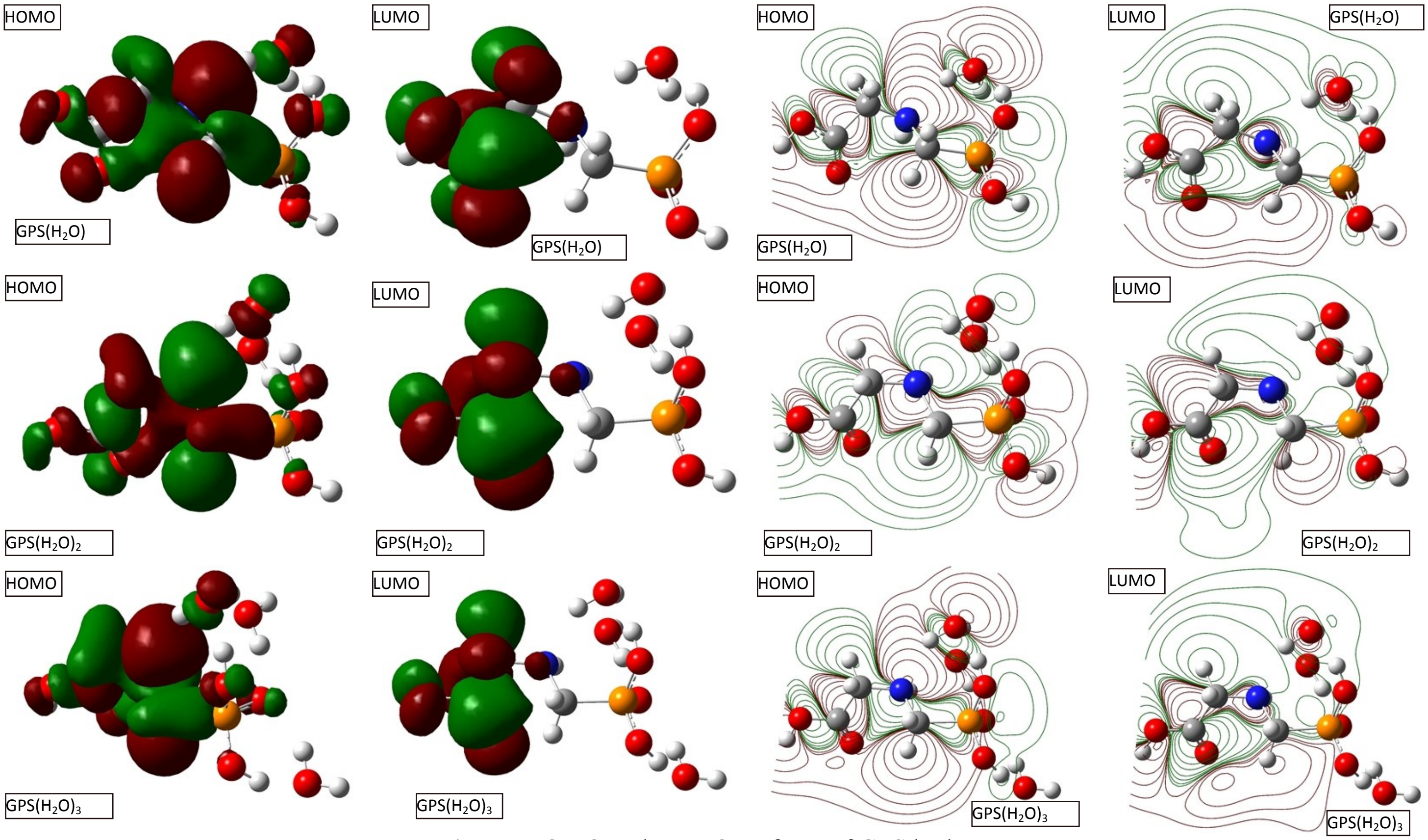

Fig. 5s. HOMO and LUMO surfaces of GPS hydrates.

Table 2s

Wavelength λ, excitation energy E, and oscillation strength *f*, of the singlet-singlet transition states of glyphosate (GPS) and its hydrates at M06-2X/6-31++G(df,p) level of DFT.

GPS				$GPS(H_2O)$				$GPS(H_2O)_2$				$GPS(H_2O)_3$			
Transition	*f*	λ (nm)	E (eV)	Transition	*f*	λ (nm)	E (eV)	Transition	*f*	λ (nm)	E (eV)	Transition	*f*	λ (nm)	E (eV)
$S_0 \rightarrow S_1$	0.0005	221.69	5.59	$S_0 \rightarrow S_1$	0.0003	218.51	5.67	$S_0 \rightarrow S_1$	0.0003	219.21	5.66	$S_0 \rightarrow S_1$	0.0003	219.96	5.64
$S_0 \rightarrow S_2$	0.0008	199.19	6.22	$S_0 \rightarrow S_2$	0.0012	187.29	6.62	$S_0 \rightarrow S_2$	0.0022	188.35	6.58	$S_0 \rightarrow S_2$	0.0017	188.01	6.59
$S_0 \rightarrow S_3$	0.0066	185.67	6.68	$S_0 \rightarrow S_3$	0.0060	177.12	7.00	$S_0 \rightarrow S_3$	0.0043	179.18	6.92	$S_0 \rightarrow S_3$	0.0060	178.46	6.95
$S_0 \rightarrow S_4$	0.0256	179.9	6.89	$S_0 \rightarrow S_4$	0.0581	169.05	7.33	$S_0 \rightarrow S_4$	0.0661	170.94	7.25	$S_0 \rightarrow S_4$	0.0452	171.03	7.25
$S_0 \rightarrow S_5$	0.0016	175.99	7.04	$S_0 \rightarrow S_5$	0.0061	166.76	7.43	$S_0 \rightarrow S_5$	0.0036	168.71	7.35	$S_0 \rightarrow S_5$	0.0250	168.64	7.35
$S_0 \rightarrow S_6$	0.0408	173.2	7.16	$S_0 \rightarrow S_6$	0.0444	164.68	7.53	$S_0 \rightarrow S_6$	0.0170	166.82	7.43	$S_0 \rightarrow S_6$	0.0198	165.71	7.48
$S_0 \rightarrow S_7$	0.0037	169.37	7.32	$S_0 \rightarrow S_7$	0.0135	160.24	7.74	$S_0 \rightarrow S_7$	0.0064	161.45	7.68	$S_0 \rightarrow S_7$	0.0167	160.89	7.71
$S_0 \rightarrow S_8$	0.0268	164.66	7.53	$S_0 \rightarrow S_8$	0.0350	158.82	7.81	$S_0 \rightarrow S_8$	0.0039	159.09	7.79	$S_0 \rightarrow S_8$	0.0097	158.47	7.82
$S_0 \rightarrow S_9$	0.0156	163.75	7.57	$S_0 \rightarrow S_9$	0.0541	158.01	7.85	$S_0 \rightarrow S_9$	0.0146	156.55	7.92	$S_0 \rightarrow S_9$	0.0107	156.47	7.92
$S_0 \rightarrow S_{10}$	0.0541	162.15	7.65	$S_0 \rightarrow S_{10}$	0.0096	155.43	7.98	$S_0 \rightarrow S_{10}$	0.0409	156.08	7.94	$S_0 \rightarrow S_{10}$	0.0296	155.96	7.95
$S_0 \rightarrow S_{11}$	0.0160	157.81	7.86	$S_0 \rightarrow S_{11}$	0.0139	152.57	8.13	$S_0 \rightarrow S_{11}$	0.0405	154.60	8.02	$S_0 \rightarrow S_{11}$	0.0111	154.79	8.01
$S_0 \rightarrow S_{12}$	0.0221	154.37	8.03	$S_0 \rightarrow S_{12}$	0.0253	151.95	8.16	$S_0 \rightarrow S_{12}$	0.0475	153.94	8.05	$S_0 \rightarrow S_{12}$	0.0586	153.68	8.07
$S_0 \rightarrow S_{13}$	0.0033	152.72	8.12	$S_0 \rightarrow S_{13}$	0.0024	149.99	8.27	$S_0 \rightarrow S_{13}$	0.0020	151.64	8.18	$S_0 \rightarrow S_{13}$	0.0046	152.25	8.14
$S_0 \rightarrow S_{14}$	0.0051	151.35	8.19	$S_0 \rightarrow S_{14}$	0.0210	147.66	8.40	$S_0 \rightarrow S_{14}$	0.0424	148.67	8.34	$S_0 \rightarrow S_{14}$	0.0616	152.02	8.16
$S_0 \rightarrow S_{15}$	0.0020	150.86	8.22	$S_0 \rightarrow S_{15}$	0.0054	146.87	8.44	$S_0 \rightarrow S_{15}$	0.0249	148.28	8.36	$S_0 \rightarrow S_{15}$	0.0023	150.62	8.23
$S_0 \rightarrow S_{16}$	0.0033	149.82	8.28	$S_0 \rightarrow S_{16}$	0.0007	146.30	8.47	$S_0 \rightarrow S_{16}$	0.0033	147.49	8.41	$S_0 \rightarrow S_{16}$	0.0484	147.55	8.40
$S_0 \rightarrow S_{17}$	0.0023	148.19	8.37	$S_0 \rightarrow S_{17}$	0.0380	145.68	8.51	$S_0 \rightarrow S_{17}$	0.0013	147.07	8.43	$S_0 \rightarrow S_{17}$	0.0024	147.34	8.41
$S_0 \rightarrow S_{18}$	0.0088	147.32	8.42	$S_0 \rightarrow S_{18}$	0.0244	145.20	8.54	$S_0 \rightarrow S_{18}$	0.0065	147.00	8.43	$S_0 \rightarrow S_{18}$	0.0004	146.57	8.46
$S_0 \rightarrow S_{19}$	0.0310	146.94	8.44	$S_0 \rightarrow S_{19}$	0.0101	144.83	8.56	$S_0 \rightarrow S_{19}$	0.0055	146.27	8.48	$S_0 \rightarrow S_{19}$	0.0088	146.12	8.49
$S_0 \rightarrow S_{20}$	0.0013	145.90	8.50	$S_0 \rightarrow S_{20}$	0.0023	144.34	8.59	$S_0 \rightarrow S_{20}$	0.0129	145.76	8.51	$S_0 \rightarrow S_{20}$	0.0655	145.72	8.51

Table 3s
Wavelength λ, excitation energy E, and oscillation strength *f*, of the singlet-singlet transition states of glyphosate (GPS) and its hydrates at M06-L/6-311++G(3df,3pd) level of DFT.

GPS				GPS(H_2O)				GPS($H_2O)_2$				GPS($H_2O)_3$			
Transition	*f*	λ (nm)	E (eV)	Transition	*f*	λ (nm)	E (eV)	Transition	*f*	λ (nm)	E (eV)	Transition	*f*	λ (nm)	E (eV)
H → L	0.0008	244.21	5.08	S0 → S1	0.0007	233.94	5.30	S0 → S1	0.0008	236.40	5.25	S0 → S1	0.0006	236.02	5.25
H → L+1	0.0113	225.75	5.49	S0 → S2	0.0002	211.83	5.85	S0 → S2	0.0153	215.66	5.75	S0 → S2	0.0088	216.51	5.73
H → L+2	0.0097	217.54	5.70	S0 → S3	0.0172	211.47	5.86	S0 → S3	0.0122	206.81	6.00	S0 → S3	0.0151	209.84	5.91
H → L+3	0.0179	206.74	5.70	S0 → S4	0.0015	206.43	6.01	S0 → S4	0.0003	204.97	6.05	S0 → S4	0.0124	202.85	6.11
H-1 → L	0.0003	203.98	6.08	S0 → S5	0.0076	201.75	6.15	S0 → S5	0.0109	202.44	6.13	S0 → S5	0.0006	201.41	6.16
H-2 → L	0.0008	199.42	6.22	S0 → S6	0.0002	197.43	6.28	S0 → S6	0.0007	198.50	6.25	S0 → S6	0.0005	197.74	6.27
**H → L+4**	0.0026	197.90	6.27	S0 → S7	0.0216	195.70	6.34	S0 → S7	0.0002	196.56	6.31	S0 → S7	0.0103	195.50	6.34
H-3 → L	0.0004	197.38	6.28	S0 → S8	0.0002	193.59	6.41	S0 → S8	0.0098	195.25	6.35	S0 → S8	0.0003	195.13	6.35
**H → L+5**	0.0042	193.81	6.40	S0 → S9	0.0022	192.53	6.44	S0 → S9	0.0001	195.01	6.36	S0 → S9	0.0006	192.89	6.43
H-1→L+1	0.0034	189.91	6.53	S0 → S10	0.0067	188.02	6.59	S0 → S10	0.0010	188.76	6.57	S0 → S10	0.0009	186.60	6.65
**H → L+6**	0.0039	187.53	6.61	S0 → S11	0.0021	187.85	6.60	S0 → S11	0.0007	188.44	6.58	S0 → S11	0.0023	186.13	6.66
H-2→L+1	0.0098	185.87	6.67	S0 → S12	0.0098	183.87	6.74	S0 → S12	0.0046	185.67	6.68	S0 → S12	0.0061	185.80	6.67
H-3→L+1	0.0169	185.05	6.70	S0 → S13	0.0179	181.00	6.85	S0 → S13	0.0220	183.19	6.77	S0 → S13	0.0191	183.57	6.75
H-1→L+2	0.0151	183.68	6.75	S0 → S14	0.0242	179.40	6.91	S0 → S14	0.0072	181.33	6.84	S0 → S14	0.0022	181.03	6.85
H-2→L+2	0.0167	179.81	6.90	S0 → S15	0.0092	179.17	6.92	S0 → S15	0.0051	181.18	6.84	S0 → S15	0.0089	180.68	6.86
H-3→L+2	0.0048	178.98	6.93	S0 → S16	0.0115	179.03	6.93	S0 → S16	0.0035	180.05	6.89	S0 → S16	0.0003	180.59	6.87
H-1→ L+3	0.0022	176.44	7.03	S0 → S17	0.0025	177.36	6.99	S0 → S17	0.0008	177.88	6.97	S0 → S17	0.0101	178.79	6.94
**H →L+7**	0.0056	173.27	7.16	S0 → S18	0.0009	176.98	7.01	S0 → S18	0.0122	177.57	6.98	S0 → S18	0.0109	178.48	6.95
H-2→L+3	0.0029	172.11	7.20	S0 → S19	0.0140	176.74	7.02	S0 → S19	0.0123	176.49	7.03	S0 → S19	0.0145	177.44	6.99
**H →L+8**	0.0089	171.93	7.21	S0 → S20	0.0008	174.51	7.11	S0 → S20	0.0002	175.23	7.08	S0 → S20	0.0052	175.92	7.05

The transition events H→L+4, H→L+5, H→L+6, and H→L+7 occasion the ionization of GPS in gaseous state.

Differently, HF/6-31+G(df) method predicts the following:

$$\text{LUMO+8} = \sigma^* = +0.04 \text{ eV}$$
$$\text{LUMO+7} = \sigma^* = -0.22 \text{ eV}$$
$$\text{LUMO+6} = \sigma^* = -0.53 \text{ eV}$$
$$\text{LUMO+5} = \sigma^* = -0.75 \text{ eV}$$
$$\text{LUMO+4} = \sigma^* = -0.80 \text{ eV}$$
$$\text{LUMO+3} = \sigma^* = -1.28 \text{ eV}$$
$$\text{LUMO+2} = \sigma^* = -1.50 \text{ eV}$$
$$\text{LUMO+1} = \sigma^* = -1.99 \text{ eV}$$
$$\text{LUMO} = \pi^* = -3.45 \text{ eV}$$
$$\text{HOMO} = n = -9.06 \text{ eV}$$
$$\text{HOMO-1} = n = -10.59 \text{ eV}$$
$$\text{HOMO-2} = n = -10.72 \text{ eV}$$
$$\text{HOMO-3} = n = -10.74 \text{ eV}$$

Therefore the transition H→L+8 is the one that occasion the ionization of the GPS.

3. Kinetics of reaction processes between glyphosate and its hydrates with •OH radicals

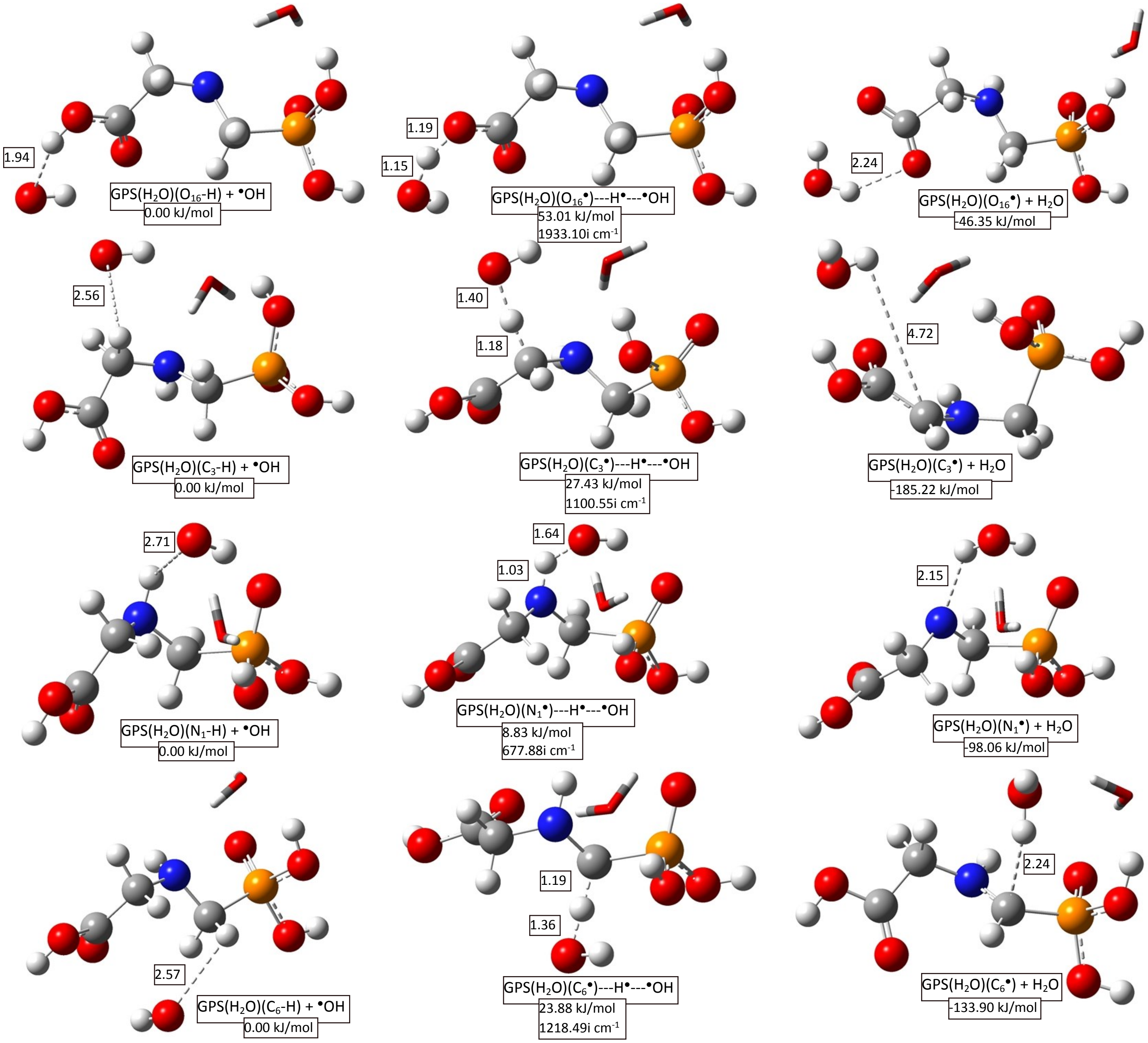

Fig. 6s. Reaction complex, transition state, and product complex for the reactions of •OH radicals with $GPS(H_2O)$ at different channels. The relative electronic energy of each step of the process are presented in kJ/mol. The internuclear bond lengths along the reaction channel and the imaginary frequency at TS are indicated.

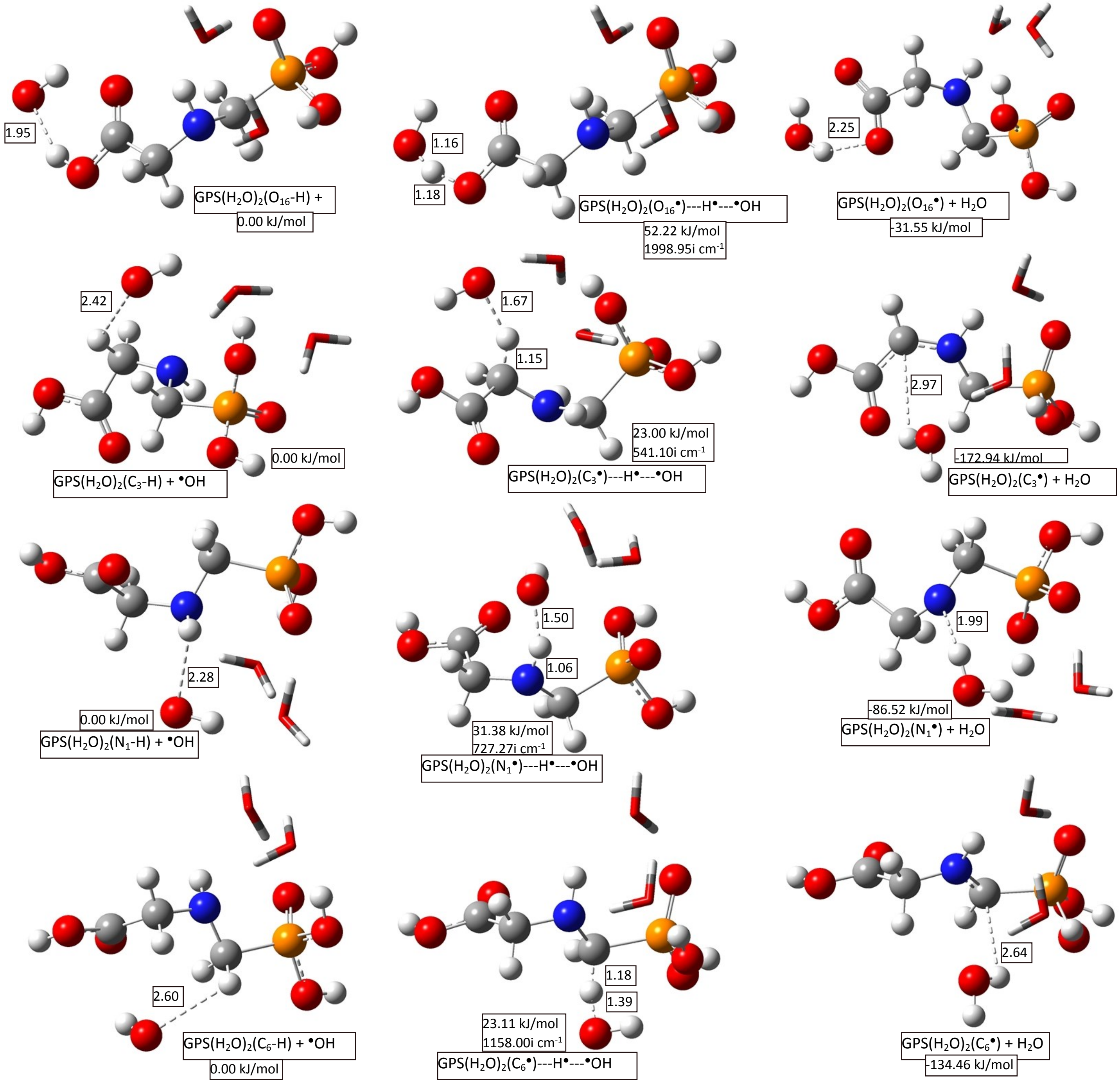

Fig. 7s. Reaction complex (RC), transition state (TS), and product complex (PC) for the reactions of $^{\bullet}OH$ radical with $GPS(H_2O)_2$ at different channels. The relative electronic energy of each step of the process are presented in kJ/mol. The internuclear bond lengths along the reaction channel and the imaginary frequency at TS are indicated.

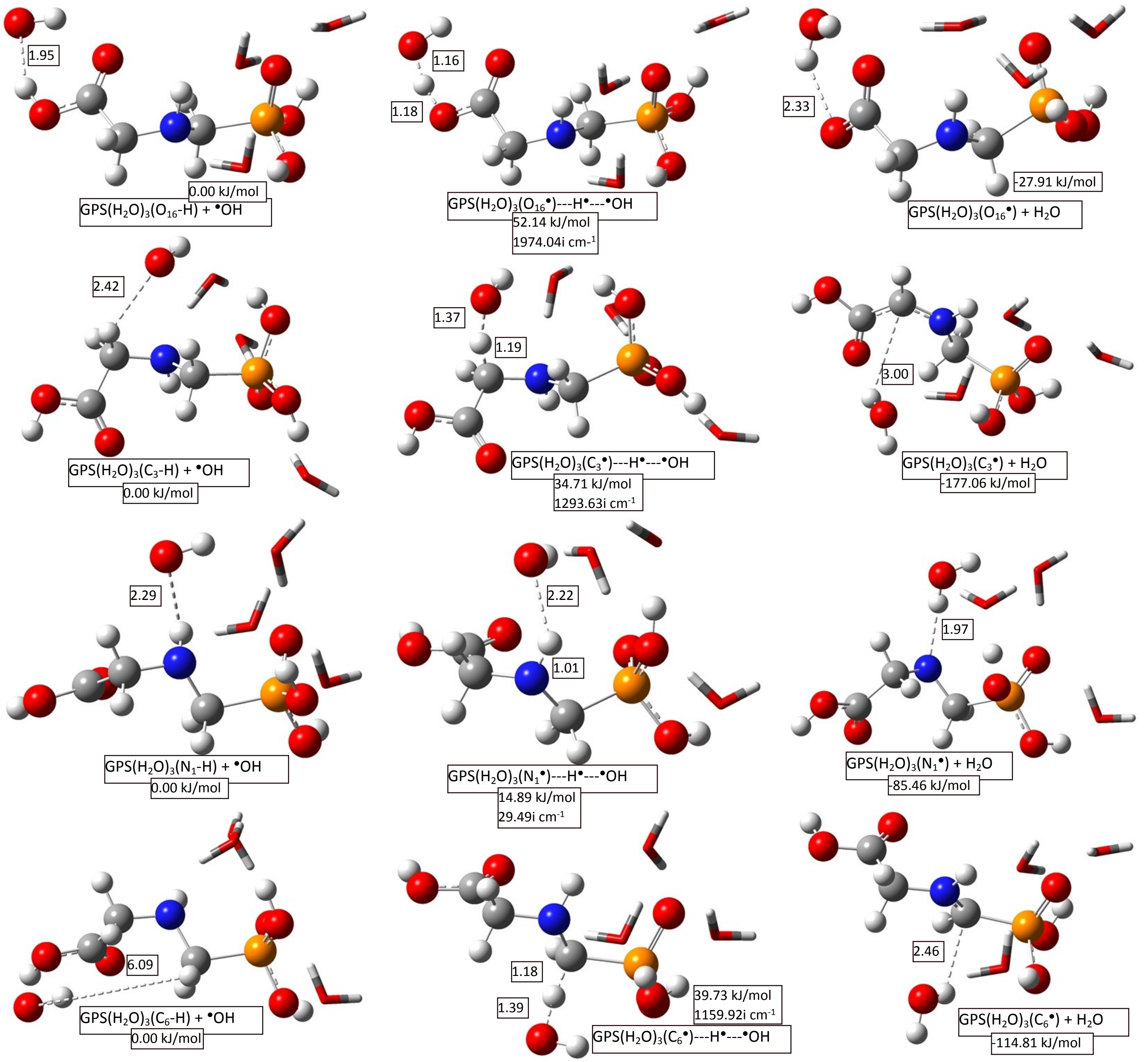

Fig. 8s. Reaction complex (RC), transition state (TS), and product complex (PC) for the reactions of •OH radical with GSP(H_2O)$_3$ at different channels. The relative electronic energy of each step of the process are presented in kJ/mol. The internuclear bond lengths along the reaction channel and the imaginary frequency at TS are indicated.

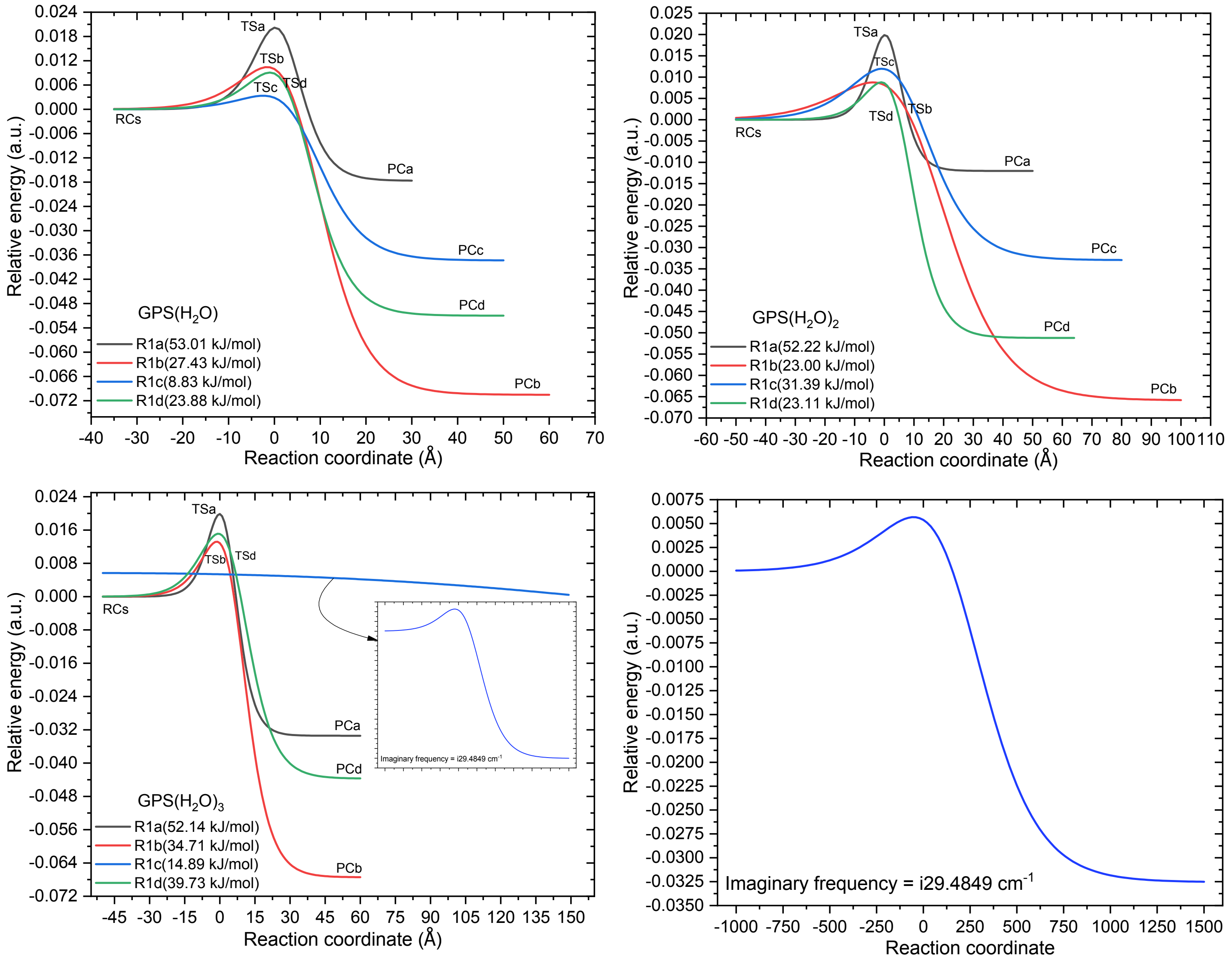

Fig. 9s. Minimum energy potentials (MEPs) of the reaction processes of GPS hydrates initiated by •OH radical. The curve at the right and lower panel is the MEP of the hydrate $GPS(H_2O)_3$ at the site N_1—H—OH. It presents a very large width, which is a result of the range parameter of the Eckart function. This parameter depends on the imaginary frequency which is much lower for this particular pathway.

Table 5s

Basis set superposition error (BSSE) corrected binding energies ($kJmol^{-1}$) of RC and TS complexes with their difference (≠)

	GPS			$GPS(H_2O)$		
	RC	TS	≠	RC	TS	≠
R1a	2.97	5.25	2.28	2.80	5.21	2.41
R1b	2.48	4.70	2.22	2.89	6.00	3.11
R1c	4.31	8.72	4.41	4.75	9.36	4.61
R1d	2.76	6.18	3.42	3.26	6.65	3.39

Table 6s Hindered internal rotor analysis and normal modes analysis for internal rotation

	IRDF	No of Rotor	Periodicity	**Symmetry number**	Multiplicity	**Reduced moment**	Vib. modes Freq. (cm^{-1})	Int. rot. modes Freq. (cm^{-1})	V/RT	Q(hin.)/Q(harm)
GPS	7	1	3	1	1	23.3971	43.472	51.1507	4.907	0.999
		2	3	1	3	20.5324	52.534	60.8738	6.099	1.000
		3	3	1	1	9.1762	67.182	76.3473	4.288	0.999
		4	3	1	3	4.1993	131.250	149.6038	7.534	1.000
		5	3	1	1	0.3062	226.637	282.0277	1.952	0.986
		6	3	1	1	0.3108	419.340	343.7438	2.944	0.995
		7	2	1	1	0.6986	651.531	594.2046	19.773	1.000
GPS(H_2O)	3	1	3	1	1	14.7594	50.792	64.4658	4.917	0.999
		2	3	1	1	0.3004	190.519	233.0903	1.308	0.966
		3	2	1	1	0.7220	646.660	580.1481	19.480	1.000
GPS(H_2O)$_2$	3	1	3	1	1	15.1849	50.775	62.1582	4.703	0.999
		2	3	1	1	0.3093	177.347	230.1238	1.313	0.966
		3	2	1	1	0.7242	654.633	592.0413	20.348	1.000
GPS(H_2O)$_3$	2	1	3	1	1	15.4909	47.512	60.9473	4.613	0.999
		2	2	1	1	0.7248	657.988	602.7965	21.111	1.000

Internal rotation degrees of freedom (IRDF)
Reduced moment in amu.Bohr^2
Vibrational modes (vib. Modes)
Internal rotation modes (Int. rot. Modes)

Table 7s Rate constant ($cm^3 molecule^{-1} s^{-1}$) of the reaction paths (R1a-R1d) over the temperature range 200 – 400 K

Reaction path	R1a	R1b	R1c	R1d	Total
T (K)	GPS + •OH				
200	4.16E-14	4.24E-16	5.51E-16	4.82E-18	4.30E-14
225	5.63E-13	5.99E-15	6.07E-15	1.84E-16	5.81E-13
250	4.73E-12	5.60E-14	4.44E-14	3.55E-15	4.89E-12
272	2.30E-11	3.05E-13	1.98E-13	1.66E-14	2.38E-11
275	2.81E-11	3.78E-13	2.39E-13	2.03E-14	2.91E-11
**298**	**1.14E-10**	**1.74E-12**	**9.07E-13**	**8.83E-14**	**1.19E-10**
325	4.76E-10	8.39E-12	3.54E-12	4.23E-13	4.97E-10
350	1.51E-09	3.01E-11	1.06E-11	1.57E-12	1.58E-09
375	4.19E-09	9.41E-11	2.83E-11	5.13E-12	4.42E-09
400	1.05E-08	2.62E-10	6.78E-11	1.51E-11	1.11E-08
T (K)	GPS(H_2O) + •OH				
200	9.06E-16	6.93E-19	8.88E-14	3.05E-15	9.58E-14
225	1.79E-14	2.57E-17	5.89E-13	4.72E-14	7.01E-13
250	2.03E-13	4.81E-16	2.80E-12	4.39E-13	3.89E-12
272	1.23E-12	2.91E-15	9.01E-12	2.29E-12	1.48E-11
275	1.54E-12	3.65E-15	1.04E-11	2.82E-12	1.76E-11
**298**	**7.56E-12**	**1.85E-14**	**2.96E-11**	**9.22E-12**	**5.56E-11**
325	3.78E-11	9.86E-14	8.57E-11	3.16E-11	1.87E-10
350	1.38E-10	3.85E-13	2.03E-10	8.67E-11	5.15E-10
375	4.34E-10	1.30E-12	4.37E-10	2.15E-10	1.30E-09
400	1.21E-09	3.86E-12	8.69E-10	4.88E-10	3.06E-09
T (K)	GPS(H_2O)$_2$ + •OH				
200	8.91E-14	9.85E-20	1.73E-17	5.18E-14	1.93E-13
225	1.17E-12	3.27E-18	2.54E-16	6.56E-13	2.48E-12
250	9.65E-12	5.73E-17	2.45E-15	5.21E-12	2.01E-11
272	4.63E-11	4.79E-16	1.36E-14	1.82E-11	8.28E-11
275	5.63E-11	6.25E-16	1.68E-14	2.14E-11	9.92E-11
**298**	**2.27E-10**	**4.09E-15**	**7.79E-14**	**6.76E-11**	**3.62E-10**
325	9.33E-10	2.73E-14	3.73E-13	2.21E-10	1.38E-09
350	2.92E-09	1.26E-13	1.32E-12	5.84E-10	4.09E-09
375	8.06E-09	4.82E-13	4.07E-12	1.40E-09	1.09E-08
400	2.00E-08	1.60E-12	1.11E-11	3.07E-09	2.61E-08
T (K)	GPS(H_2O)$_3$ + •OH				
200	1.98E-17	8.45E-20	7.31E-18	7.87E-18	4.31E-17
225	5.76E-16	4.64E-18	1.37E-16	2.90E-16	1.30E-15
250	8.91E-15	1.19E-16	1.48E-15	5.41E-15	2.14E-14
272	6.75E-14	1.30E-15	8.62E-15	2.54E-14	1.29E-13
275	8.70E-14	1.75E-15	1.07E-14	3.10E-14	1.63E-13
**298**	**5.20E-13**	**8.91E-15**	**5.10E-14**	**1.33E-13**	**8.56E-13**
325	3.17E-12	4.90E-14	2.47E-13	6.27E-13	4.77E-12
350	1.35E-11	2.00E-13	8.75E-13	2.28E-12	1.94E-11
375	4.88E-11	7.13E-13	2.68E-12	7.31E-12	6.75E-11
400	1.53E-10	2.25E-12	7.26E-12	2.11E-11	2.07E-10

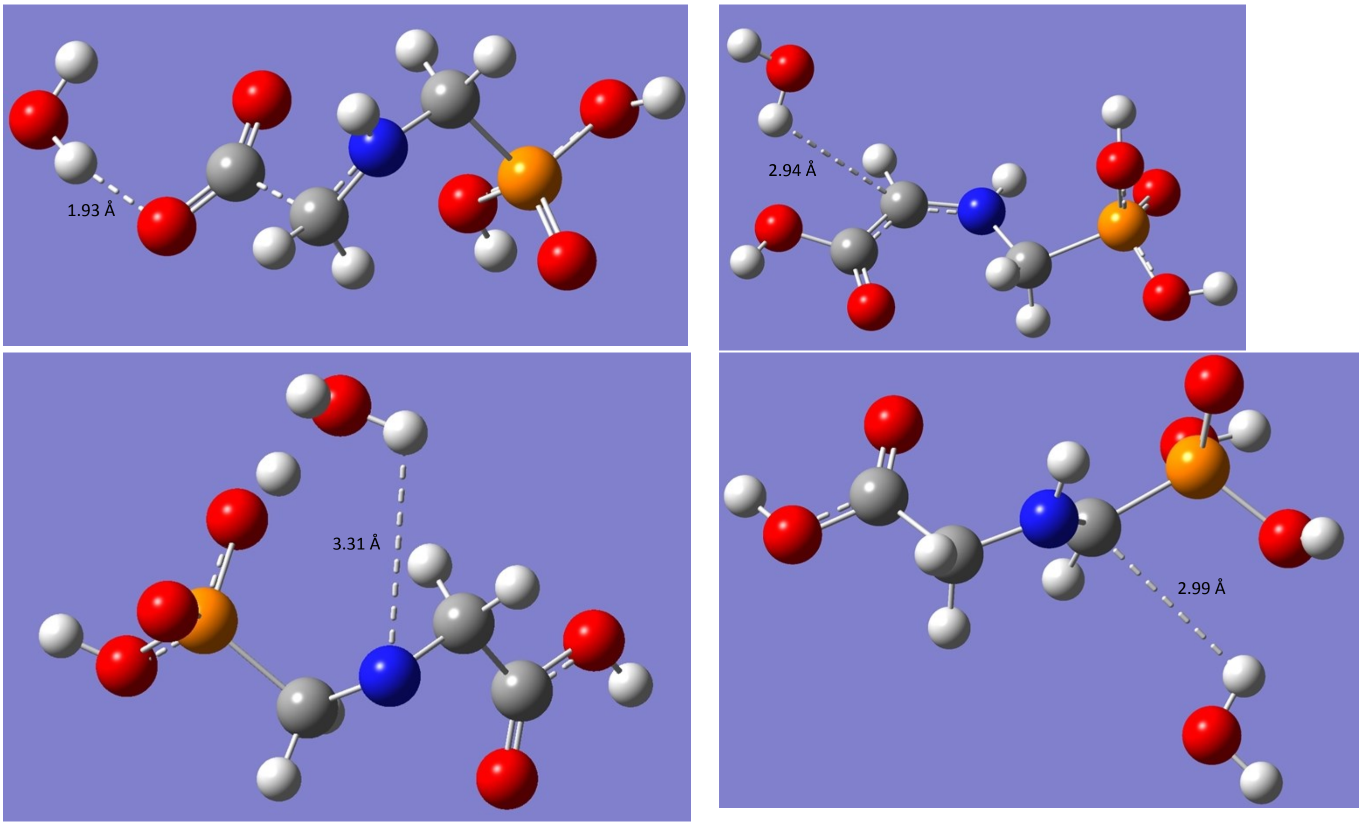

Fig. 10s Product complexes from each pathway in water continuum. The reaction complexes are unstable.

Table 8s Cartesian coordinates of reaction complexes, transition states, and product complexes of the reaction processes of glyphosate and its hydrates with the $^{\bullet}$OH radical

Coordinate of reaction complex: GPS-C_3H+$^{\bullet}$OH

		X	y	Z
N	7.0	0.20428	0.30059	0.59836
H	1.0	0.05016	-0.24833	1.43958
C	6.0	1.60719	0.41726	0.29669
H	1.0	1.75197	1.05392	-0.58490
H	1.0	2.13041	0.91777	1.12084
C	6.0	-0.61652	-0.21748	-0.48993
H	1.0	-0.45959	-1.28106	-0.71620
H	1.0	-0.43654	0.36983	-1.39661
P	15.0	-2.33133	0.04755	0.01772
O	8.0	-2.56635	1.63253	-0.08225
H	1.0	-2.29719	2.08804	0.72538
O	8.0	-3.14844	-0.48143	-1.24916
H	1.0	-4.04705	-0.76244	-1.03633
O	8.0	-2.67884	-0.53967	1.32797
C	6.0	2.30822	-0.90979	0.05210
O	8.0	3.58631	-0.73416	-0.36893
H	1.0	3.98638	-1.60962	-0.48654
O	8.0	1.83013	-1.99920	0.21497
O	8.0	4.06450	2.08852	-0.13437
H	1.0	4.36937	1.18202	-0.33853

Coordinate of product complex: GPS-C_3+H_2O

		X	y	Z
N	7.0	-0.23634	0.52727	-0.35491
H	1.0	0.05692	1.47245	-0.57124
C	6.0	-1.56652	0.32011	-0.19240
H	1.0	-5.15422	2.23696	0.28476
H	1.0	-2.19830	1.19972	-0.18063
C	6.0	0.77926	-0.49165	-0.54127
H	1.0	0.87732	-0.78726	-1.59449
H	1.0	0.53934	-1.38220	0.03961
P	15.0	2.34569	0.23451	0.00150
O	8.0	2.46526	-0.01615	1.58341
H	1.0	2.35017	0.79966	2.08667
O	8.0	3.38209	-0.81574	-0.60069
H	1.0	4.31277	-0.59937	-0.46483
O	8.0	2.49321	1.65978	-0.36599
C	6.0	-2.14296	-0.96815	0.00012
O	8.0	-3.50981	-0.90098	0.15010
H	1.0	-3.80915	-1.81197	0.27540
O	8.0	-1.57618	-2.05481	0.04340
O	8.0	-4.31770	1.83127	0.04536
H	1.0	-4.41937	0.87461	0.14315

Coordinate of transition state: GPS-C_3—H--$^{\bullet}$OH

		X	y	Z
N	7.0	-0.28916	-0.09485	-0.90386
H	1.0	0.00732	-0.79546	-1.57400
C	6.0	-1.67891	0.17162	-0.87948
H	1.0	-1.84868	1.23993	-0.45562
H	1.0	-2.09694	0.19229	-1.89066
C	6.0	0.38568	-0.20393	0.37780
H	1.0	0.18302	-1.13881	0.91686
H	1.0	0.09581	0.64268	1.00981
P	15.0	2.15419	-0.08911	0.02166
O	8.0	2.47130	1.46416	-0.23522
H	1.0	2.47433	1.68284	-1.17530
O	8.0	2.78444	-0.32377	1.46964
H	1.0	3.73526	-0.48903	1.46789

Coordinate of reaction complex: GPS-C_6H+$^{\bullet}$OH

		X	y	Z
N	7.0	0.28312	0.57418	0.50882
H	1.0	0.39718	-0.28372	1.04104
C	6.0	1.55264	1.08532	0.05917
H	1.0	1.41072	2.03300	-0.47185
H	1.0	2.19322	1.30892	0.91983
C	6.0	-0.70044	0.37434	-0.54789
H	1.0	-0.47953	-0.45966	-1.22790
H	1.0	-0.79934	1.29384	-1.13457
P	15.0	-2.27816	0.07549	0.28191
O	8.0	-2.72822	1.49541	0.88440
H	1.0	-2.42983	1.60891	1.79533
O	8.0	-3.27056	-0.08346	-0.96061
H	1.0	-4.11343	-0.49711	-0.73712

O	8.0	2.60669	-0.97262	-1.07379
C	6.0	-2.52758	-0.74432	-0.01600
O	8.0	-3.74420	-0.20420	0.21870
H	1.0	-4.26002	-0.83970	0.73782
O	8.0	-2.19146	-1.81574	0.41213
O	8.0	-2.24074	2.36626	0.39317
H	1.0	-3.14221	2.05282	0.59428

O	8.0	-2.26110	-1.01741	1.27560
C	6.0	2.33671	0.14939	-0.84652
O	8.0	3.41535	0.73635	-1.35927
H	1.0	3.90801	0.09387	-1.91036
O	8.0	2.02473	-1.00477	-1.06026
O	8.0	4.18462	-1.66188	-2.70704
H	1.0	3.34753	-1.90571	-2.24717

Coordinate of product complex: GPS-C_6+H_2O

		X	y	Z
N	7.0	0.61736	0.41608	0.48497
H	1.0	0.26483	0.99228	1.23796
C	6.0	1.86319	0.79061	-0.13082
H	1.0	1.68927	1.23891	-1.12024
H	1.0	2.37090	1.53289	0.48916
C	6.0	-0.28212	-0.41044	-0.12428
H	1.0	0.06592	-1.08942	-0.89040
H	1.0	-1.05301	1.28442	-2.13434
P	15.0	-1.96398	-0.15036	0.24630
O	8.0	-2.66304	0.70335	-0.97279
H	1.0	-3.02353	1.52613	-0.61401
O	8.0	-2.60430	-1.59168	-0.00528
H	1.0	-3.56476	-1.58897	-0.09760
O	8.0	-2.20809	0.55629	1.52536
C	6.0	2.79995	-0.38750	-0.31600
O	8.0	3.99380	0.03739	-0.77317
H	1.0	4.55264	-0.74222	-0.90873
O	8.0	2.54117	-1.54417	-0.12175
O	8.0	-0.20326	1.67530	-2.38201
H	1.0	-0.31300	2.03385	-3.26713

Coordinate of transition state: GPS-C_6—H--$^\bullet$OH

		X	y	Z
N	7.0	-0.58093	-0.07707	-0.89842
H	1.0	-0.51482	-1.00992	-1.29544
C	6.0	-1.92923	0.42863	-0.84689
H	1.0	-1.90941	1.47515	-0.52121
H	1.0	-2.37273	0.41611	-1.84783
C	6.0	0.15458	0.03243	0.32760
H	1.0	-0.26169	-0.47842	1.20761
H	1.0	0.18443	1.17138	0.62457
P	15.0	1.86775	-0.42382	-0.00219
O	8.0	2.48287	0.87353	-0.74579
H	1.0	2.65473	0.69300	-1.67900
O	8.0	2.50443	-0.33293	1.45620
H	1.0	3.35475	-0.78056	1.54806
O	8.0	2.08294	-1.67023	-0.76431
C	6.0	-2.85518	-0.34548	0.07813
O	8.0	-4.07076	0.22398	0.13936
H	1.0	-4.62218	-0.30456	0.73592
O	8.0	-2.56179	-1.34596	0.68106
O	8.0	0.33387	2.56159	0.51216
H	1.0	1.14365	2.54128	-0.03340

Coordinate of reaction complex: GPS-N_1H+$^\bullet$OH

		X	y	Z
N	7.0	-0.62757	0.24577	-1.12068
H	1.0	-0.14689	0.99145	-1.60678
C	6.0	-1.56187	0.71500	-0.12806
H	1.0	-1.14596	0.85558	0.88252
H	1.0	-1.95174	1.68863	-0.43503
C	6.0	0.20910	-0.89352	-0.80157
H	1.0	0.52096	-1.40341	-1.71888
H	1.0	-0.35696	-1.61398	-0.20580
P	15.0	1.71401	-0.38654	0.07524
O	8.0	1.24829	0.18949	1.49827
H	1.0	1.12168	1.15327	1.45727
O	8.0	2.41953	-1.76686	0.44178
H	1.0	3.35174	-1.68943	0.68202
O	8.0	2.54712	0.58937	-0.68345
C	6.0	-2.73353	-0.23506	0.02811
O	8.0	-3.67929	0.30855	0.81832
H	1.0	-4.39074	-0.34066	0.92231
O	8.0	-2.82394	-1.33887	-0.44062
O	8.0	0.84941	2.59592	-0.06055
H	1.0	1.70955	2.29679	-0.44246

Coordinate of product complex: GPS-N_1+H_2O

		X	y	Z
N	7.0	-0.59323	0.55642	-0.73318
H	1.0	0.73042	2.33610	-0.70525
C	6.0	-1.63296	0.73415	0.24507
H	1.0	-1.27729	0.45885	1.25258
H	1.0	-1.95152	1.77930	0.27914
C	6.0	0.01731	-0.74374	-0.67872
H	1.0	0.17413	-1.12385	-1.69368
H	1.0	-0.52344	-1.49173	-0.08611
P	15.0	1.68835	-0.51742	0.04312
O	8.0	1.43564	0.32032	1.37505
H	1.0	1.47248	1.28043	1.15585
O	8.0	1.99256	-1.99161	0.57488
H	1.0	2.93655	-2.19229	0.61459
O	8.0	2.70264	0.05846	-0.86868
C	6.0	-2.85923	-0.12237	-0.04372
O	8.0	-3.87267	0.22871	0.76376
H	1.0	-4.63061	-0.34119	0.56243
O	8.0	-2.93228	-0.99500	-0.86648
O	8.0	1.48550	2.59224	-0.15329
H	1.0	2.25475	2.24759	-0.63184

Coordinate of transition state: GPS-N_1—H--$^\bullet$OH

		X	y	Z
N	7.0	-0.60787	0.50179	-0.85306
H	1.0	-0.17804	1.42423	-1.11709
C	6.0	-1.57712	0.65338	0.20460
H	1.0	-1.16315	0.41763	1.19698
H	1.0	-1.91694	1.69034	0.23184
C	6.0	0.23653	-0.66780	-0.83235
H	1.0	0.50179	-0.96078	-1.85220
H	1.0	-0.29516	-1.49513	-0.35551
P	15.0	1.80258	-0.34975	0.07164
O	8.0	1.34964	0.31272	1.44567
H	1.0	1.10559	1.24897	1.25119
O	8.0	2.25402	-1.82034	0.49760
H	1.0	3.21289	-1.93548	0.49093
O	8.0	2.79025	0.42088	-0.71692
C	6.0	-2.76535	-0.26611	-0.01449
O	8.0	-3.75190	0.03697	0.84364
H	1.0	-4.48124	-0.58450	0.69766
O	8.0	-2.82498	-1.16268	-0.81269
O	8.0	0.62900	2.37231	-0.15661
H	1.0	1.49806	2.33282	-0.59905

Coordinate of reaction complex: GPS-O_{16}H+$^\bullet$OH

		X	y	Z
N	7.0	-0.24164	1.74209	0.74297
H	1.0	-0.88695	2.49405	0.53260
C	6.0	0.65863	1.56337	-0.37875
H	1.0	0.17594	1.24362	-1.31556
H	1.0	1.14921	2.51950	-0.58970
C	6.0	-0.97152	0.56803	1.19501
H	1.0	-1.72002	0.88165	1.92781
H	1.0	-0.29363	-0.13272	1.69034
P	15.0	-1.85936	-0.32545	-0.11597
O	8.0	-0.66133	-1.17834	-0.76722
H	1.0	-0.88042	-1.57127	-1.62221
O	8.0	-2.73613	-1.43310	0.65526
H	1.0	-3.67841	-1.33905	0.46673
O	8.0	-2.64690	0.48804	-1.06901
C	6.0	1.75662	0.56721	-0.08795
O	8.0	2.50389	0.35072	-1.17032
H	1.0	3.20898	-0.29209	-0.95463
O	8.0	1.95053	0.03227	0.98352
O	8.0	4.13880	-1.52195	0.25975
H	1.0	3.45401	-1.20946	0.89767

Coordinate of product complex: GPS-O_{16}+H_2O

		X	y	Z
N	7.0	0.03410	-0.82857	-0.82029

Coordinate of transition state: GPS-O_{16}—H--$^\bullet$OH

		X	y	Z
N	7.0	0.01672	-0.80594	-1.01111

H	1.0	0.01211	-1.32768	-1.69844
C	6.0	0.93436	-1.25151	0.15554
H	1.0	0.54316	-1.07577	1.16579
H	1.0	1.18497	-2.29872	0.01177
C	6.0	-0.68495	0.41394	-0.73207
H	1.0	-0.81775	0.85752	-1.72164
H	1.0	-0.12422	1.11547	-0.10356
P	15.0	-2.33839	0.14080	-0.01480
O	8.0	-1.92519	-0.39101	1.43895
H	1.0	-2.59461	-0.95245	1.85171
O	8.0	-2.85019	1.64001	0.20736
H	1.0	-3.74857	1.78445	-0.11688
O	8.0	-3.27502	-0.72313	-0.75813
C	6.0	2.20986	-0.42401	0.15908
O	8.0	3.19578	-1.16034	0.16251
H	1.0	4.88931	0.29903	-0.01726
O	8.0	2.02792	0.79166	0.19106
O	8.0	4.91871	1.25858	-0.10752
H	1.0	4.00100	1.52948	0.02339

H	1.0	-0.46868	-1.61181	-1.38009
C	6.0	0.93547	-1.10787	0.04060
H	1.0	0.55135	-0.90755	1.05382
H	1.0	1.23209	-2.15537	-0.00787
C	6.0	-0.80494	0.37891	-0.89881
H	1.0	-1.12900	0.71022	-1.89062
H	1.0	-0.22422	1.19380	-0.45864
P	15.0	-2.30854	0.08794	0.07606
O	8.0	-1.70257	-0.06498	1.55730
H	1.0	-2.29397	-0.52542	2.16707
O	8.0	-3.05750	1.50935	0.09062
H	1.0	-3.96975	1.44442	-0.21921
O	8.0	-3.17229	-1.03226	-0.35679
C	6.0	2.20808	-0.22309	-0.06283
O	8.0	3.24584	-0.84804	0.41223
H	1.0	4.17960	-0.15365	0.35577
O	8.0	2.18003	0.91043	-0.49111
O	8.0	4.79998	0.84552	0.29396
H	1.0	4.25405	1.47992	-0.20682

Coordinate of reaction complex: GPS(H_2O)-C_3H+$^{\bullet}$OH

		X	y	Z
N	7.0	0.55066	0.17398	0.19043
H	1.0	0.62623	0.13886	1.20596
C	6.0	1.87722	0.30377	-0.38771
H	1.0	1.81276	0.27658	-1.47972
H	1.0	2.29715	1.28280	-0.12853
C	6.0	-0.18971	-1.01805	-0.25045
H	1.0	0.11405	-1.93237	0.27164
H	1.0	-0.04606	-1.16128	-1.32547
P	15.0	-1.94786	-0.66899	0.04551
O	8.0	-2.43571	0.22325	-1.18627
H	1.0	-2.23883	1.16805	-1.02941
O	8.0	-2.61809	-2.08333	-0.25919
H	1.0	-3.53762	-2.16037	0.02387
O	8.0	-2.20646	-0.06267	1.37243
C	6.0	2.83472	-0.77474	0.08271
O	8.0	3.98544	-0.75499	-0.60520
H	1.0	4.56824	-1.43965	-0.24288
O	8.0	2.60629	-1.54860	0.97787
O	8.0	-1.14094	2.39507	0.03150
H	1.0	-1.65881	2.32416	0.84523
H	1.0	-0.47184	1.66522	0.09825
O	8.0	1.34668	3.65517	-0.25751
H	1.0	0.37040	3.53888	-0.20444

Coordinate of product complex: GPS(H_2O)-C_3+H_2O

		X	y	Z
N	7.0	0.32882	1.67352	0.55485
H	1.0	0.09590	1.30292	1.47298
C	6.0	-0.70585	1.65956	-0.32456
H	1.0	-0.55083	2.01080	-1.33567
H	1.0	-3.85669	-2.18069	-1.14055
C	6.0	1.69904	1.50116	0.11672
H	1.0	2.39362	1.92585	0.84486
H	1.0	1.84785	1.99618	-0.84651
P	15.0	2.02743	-0.28797	-0.05386
O	8.0	1.03652	-0.79021	-1.19002
H	1.0	0.22803	-1.19121	-0.78895
O	8.0	3.46316	-0.27299	-0.75163
H	1.0	3.93448	-1.11490	-0.71292
O	8.0	1.91335	-1.04598	1.21675
C	6.0	-1.93660	1.07490	0.10748
O	8.0	-2.92156	1.09702	-0.79529
H	1.0	-3.52314	0.35797	-0.57235
O	8.0	-2.05604	0.53451	1.21669
O	8.0	-0.82169	-1.78558	0.56228
H	1.0	-0.08264	-2.09246	1.10922
H	1.0	-1.20919	-1.02548	1.04438
O	8.0	-3.56171	-1.59026	-0.44220
H	1.0	-2.74622	-1.96975	-0.08107

Coordinate of transition state: GPS(H_2O)-C_3—H--$^{\bullet}$OH

		X	y	Z
N	7.0	0.52838	-0.14404	0.72023
H	1.0	1.07266	-0.34805	1.55361
C	6.0	1.42280	0.24635	-0.33367
H	1.0	0.98435	0.10805	-1.32428
H	1.0	1.66268	1.40203	-0.27624
C	6.0	-0.35276	-1.27591	0.39861
H	1.0	-0.58159	-1.83048	1.31166
H	1.0	0.08827	-1.97536	-0.32299
P	15.0	-1.93030	-0.62282	-0.22948
O	8.0	-1.53146	0.33264	-1.44841
H	1.0	-1.38071	1.24316	-1.12233
O	8.0	-2.53992	-1.90890	-0.95316
H	1.0	-3.49763	-1.87205	-1.06976
O	8.0	-2.78321	0.02566	0.79178
C	6.0	2.78571	-0.39488	-0.22706
O	8.0	3.54903	-0.12650	-1.29115
H	1.0	4.42615	-0.51110	-1.14272
O	8.0	3.15056	-1.04760	0.71976
O	8.0	-0.92538	2.31558	0.43643
H	1.0	-1.79443	2.45423	0.83308
H	1.0	-0.54583	1.54747	0.90930
O	8.0	1.80574	2.78679	-0.14802
H	1.0	0.86729	3.02608	0.00359

Coordinate of reaction complex: GPS(H_2O)-C_6H+$^{\bullet}$OH

		X	y	Z
N	7.0	0.35827	0.85783	0.07280
H	1.0	0.30240	1.04788	1.07145
C	6.0	1.68740	1.13567	-0.42644
H	1.0	1.75253	0.86336	-1.48556
H	1.0	1.89691	2.20906	-0.36299
C	6.0	-0.11788	-0.50644	-0.16978
H	1.0	0.36510	-1.26233	0.46400
H	1.0	0.04222	-0.77486	-1.21813
P	15.0	-1.90140	-0.51051	0.17098
O	8.0	-2.63948	0.01163	-1.14025
H	1.0	-2.67935	0.99481	-1.12998
O	8.0	-2.21692	-2.07442	0.15501
H	1.0	-3.13133	-2.30161	0.36377
O	8.0	-2.24436	0.24159	1.40326
C	6.0	2.80503	0.41044	0.30020
O	8.0	4.00324	0.73603	-0.19941
H	1.0	4.68433	0.25297	0.29249
O	8.0	2.65728	-0.35911	1.22012
O	8.0	-2.08503	2.57268	-0.37577
H	1.0	-2.40590	2.37297	0.51595
H	1.0	-1.14990	2.30576	-0.35052
O	8.0	2.21771	-2.13082	-0.98566
H	1.0	2.54923	-2.27388	-0.07697

Coordinate of product complex: GPS(H_2O)-C_6+H_2O

		X	y	Z
N	7.0	0.86211	0.00594	0.74592
H	1.0	0.32133	0.17227	1.58825
C	6.0	2.12166	0.68418	0.61335
H	1.0	2.03367	1.59849	0.00498
H	1.0	2.47467	0.99324	1.59977

Coordinate of transition state: GPS(H_2O)-C_6—H--$^{\bullet}$OH

		X	y	Z
N	7.0	-0.64910	0.62072	-0.40427
H	1.0	-0.65007	0.52727	-1.41974
C	6.0	-1.98392	0.88809	0.09634
H	1.0	-1.95142	0.96265	1.18830
H	1.0	-2.33847	1.85323	-0.28062

C	6.0	0.13748	-0.44314	-0.32529
H	1.0	0.67097	-0.76907	-1.21030
H	1.0	-0.26096	1.67519	-0.93159
P	15.0	-1.53226	-0.88764	0.04131
O	8.0	-2.51290	-0.30808	-1.07415
H	1.0	-2.85193	0.57849	-0.78206
O	8.0	-1.62000	-2.45326	-0.26675
H	1.0	-2.52041	-2.76737	-0.41969
O	8.0	-1.86410	-0.43076	1.42131
C	6.0	3.19584	-0.18282	-0.01727
O	8.0	4.37032	0.47367	-0.02185
H	1.0	5.03072	-0.09501	-0.44527
O	8.0	3.04773	-1.28310	-0.47432
O	8.0	-2.98047	1.95611	0.26461
H	1.0	-2.83727	1.45556	1.08360
H	1.0	-2.16949	2.47013	0.12126
O	8.0	-0.36098	2.60611	-0.67305
H	1.0	-0.30891	3.12617	-1.48129

C	6.0	-0.03780	-0.56012	0.17340
H	1.0	-0.54728	-1.51197	-0.02593
H	1.0	-0.07702	-0.42756	1.35415
P	15.0	1.72085	-0.56738	-0.26388
O	8.0	2.42963	0.37644	0.81545
H	1.0	2.38446	1.31362	0.50031
O	8.0	2.15864	-2.02877	0.19364
H	1.0	3.03279	-2.30142	-0.11154
O	8.0	1.98214	-0.18041	-1.66979
C	6.0	-3.00693	-0.16126	-0.30286
O	8.0	-4.19168	0.05949	0.28564
H	1.0	-4.81114	-0.62485	-0.01026
O	8.0	-2.80067	-1.06988	-1.06640
O	8.0	1.64827	2.56339	-0.52565
H	1.0	1.93434	2.23790	-1.39082
H	1.0	0.73248	2.24503	-0.43650
O	8.0	0.04840	0.10022	2.60269
H	1.0	0.99641	0.32762	2.53479

Coordinate of reaction complex: GPS(H_2O)-N_1H+$^{\bullet}$OH

		X	y	Z
N	7.0	0.70964	-0.59468	1.39842
H	1.0	0.21656	-0.11313	2.14077
C	6.0	1.51628	0.32432	0.62652
H	1.0	0.97906	0.90801	-0.14143
H	1.0	1.96607	1.05806	1.30122
C	6.0	-0.16460	-1.48329	0.65886
H	1.0	-0.59753	-2.21800	1.34495
H	1.0	0.40790	-2.03399	-0.09215
P	15.0	-1.56875	-0.63352	-0.12912
O	8.0	-1.14630	-0.05870	-1.54695
H	1.0	-0.95919	0.91581	-1.54276
O	8.0	-2.52101	-1.85900	-0.50056
H	1.0	-3.26490	-1.63790	-1.07475
O	8.0	-2.16730	0.40515	0.76593
C	6.0	2.63703	-0.38926	-0.10174
O	8.0	3.47355	0.50056	-0.67016
H	1.0	4.15028	0.00293	-1.15241
O	8.0	2.77778	-1.57924	-0.20563
O	8.0	-0.49243	2.49650	-1.24809
H	1.0	-0.55546	2.77230	-0.31409
H	1.0	-0.63032	3.26350	-1.80987
O	8.0	-0.64009	2.38750	1.53064
H	1.0	-1.35440	1.69512	1.38514

Coordinate of product complex: GPS(H_2O)-N_1+H_2O

		X	y	Z
N	7.0	-0.64847	0.26557	-0.81818
H	1.0	0.57547	1.81710	-1.67620
C	6.0	-1.64396	0.58580	0.17089
H	1.0	-1.29744	0.33828	1.18803
H	1.0	-1.86848	1.65492	0.15305
C	6.0	-0.09551	-1.05014	-0.66710
H	1.0	0.08230	-1.49518	-1.65145
H	1.0	-0.68758	-1.73796	-0.05050
P	15.0	1.55638	-0.84720	0.10862
O	8.0	1.27168	-0.16849	1.49951
H	1.0	1.19599	0.84268	1.45632
O	8.0	1.92746	-2.35700	0.47852
H	1.0	2.86194	-2.55218	0.33322
O	8.0	2.55209	-0.17253	-0.76402
C	6.0	-2.93593	-0.18560	-0.06044
O	8.0	-3.91406	0.28868	0.72807
H	1.0	-4.71271	-0.23541	0.56366
O	8.0	-3.08292	-1.09923	-0.82763
O	8.0	0.92777	2.34226	1.15227
H	1.0	1.13800	2.49570	0.18796
H	1.0	1.34484	3.03350	1.67424
O	8.0	1.38669	2.29096	-1.43001
H	1.0	2.06401	1.59010	-1.43417

Coordinate of transition state: GPS(H_2O)-N_1—H--$^{\bullet}$OH

		X	y	Z
N	7.0	0.66680	-0.00003	1.04661
H	1.0	0.39148	0.64585	1.80549
C	6.0	1.59995	0.51864	0.08170
H	1.0	1.15893	0.62006	-0.91917
H	1.0	1.92108	1.51544	0.39125
C	6.0	-0.16703	-1.12469	0.72309
H	1.0	-0.45673	-1.64897	1.63762
H	1.0	0.38878	-1.81599	0.08204
P	15.0	-1.70967	-0.62431	-0.14402
O	8.0	-1.24271	0.08762	-1.46240
H	1.0	-1.05929	1.09431	-1.37277
O	8.0	-2.25547	-2.04830	-0.62874
H	1.0	-3.21643	-2.11810	-0.56698
O	8.0	-2.64636	0.12582	0.73171
C	6.0	2.81215	-0.38967	-0.04674
O	8.0	3.74701	0.18889	-0.81569
H	1.0	4.49040	-0.42662	-0.90393
O	8.0	2.92682	-1.48402	0.43772
O	8.0	-0.58420	2.47440	-1.00943
H	1.0	-0.57109	2.42784	0.00761
H	1.0	-1.07516	3.25102	-1.29150
O	8.0	-0.57024	1.93614	1.47775
H	1.0	-1.46378	1.54997	1.56887

Coordinate of reaction complex: GPS(H_2O)-O_{16}H+$^{\bullet}$OH

		X	y	Z
N	7.0	0.06506	0.83854	0.06935
H	1.0	0.13337	0.66825	1.07160
C	6.0	1.32700	1.32978	-0.44057
H	1.0	1.26873	1.46554	-1.52510
H	1.0	1.54469	2.31632	-0.01533
C	6.0	-0.39033	-0.39941	-0.57201
H	1.0	0.23282	-1.27130	-0.33460
H	1.0	-0.41294	-0.26296	-1.65746
P	15.0	-2.08493	-0.70079	0.00610
O	8.0	-3.06535	0.14451	-0.92165
H	1.0	-3.14149	1.06241	-0.57402
O	8.0	-2.32259	-2.19403	-0.50478
H	1.0	-3.17971	-2.56810	-0.26662
O	8.0	-2.23960	-0.43937	1.45775
C	6.0	2.51848	0.44115	-0.12597
O	8.0	3.61652	0.83189	-0.76458
H	1.0	4.36932	0.26051	-0.50573
O	8.0	2.47126	-0.49830	0.64167
O	8.0	-2.48732	2.36295	0.54604
H	1.0	-2.63888	1.87891	1.37062
H	1.0	-1.54787	2.20474	0.34860
O	8.0	5.17517	-1.20522	0.48203
H	1.0	4.27502	-1.36078	0.85064

Coordinate of product complex: GPS(H_2O)-O_{16}+H_2O

		X	y	Z
N	7.0	-0.28572	-0.74911	-0.01794
H	1.0	-0.00167	-1.03242	0.91529
C	6.0	-1.41359	-1.32892	-0.56092
H	1.0	-1.44340	-1.24608	-1.65240
H	1.0	-1.54706	-2.35240	-0.22173
C	6.0	0.18909	0.54905	-0.43165

Coordinate of transition state: GPS(H_2O)-O_{16}—H--$^{\bullet}$OH

		X	y	Z
N	7.0	0.09740	0.81072	0.08403
H	1.0	0.10742	0.72667	1.09910
C	6.0	1.36883	1.26424	-0.40805
H	1.0	1.35175	1.34547	-1.49761
H	1.0	1.60491	2.25307	-0.00290
C	6.0	-0.35963	-0.45717	-0.48774

H	1.0	-0.36707	1.35355	0.06816
H	1.0	0.06346	0.66969	-1.51053
P	15.0	1.94103	0.65249	0.03051
O	8.0	2.78430	0.01105	-1.14767
H	1.0	3.36569	-0.71989	-0.82725
O	8.0	2.16313	2.22341	-0.09773
H	1.0	3.06379	2.53622	0.05285
O	8.0	2.19877	0.02933	1.35724
C	6.0	-2.78396	-0.54146	-0.18927
O	8.0	-3.67924	-1.36157	-0.05422
H	1.0	-5.40498	-0.03529	0.47580
O	8.0	-2.64563	0.67175	-0.16722
O	8.0	4.16039	-1.64043	0.41852
H	1.0	3.64253	-1.32594	1.17886
H	1.0	4.48977	-2.52452	0.59643
O	8.0	-5.51593	0.91403	0.60403
H	1.0	-4.65198	1.28086	0.37984

H	1.0	0.22845	-1.32034	-0.14871
H	1.0	-0.31746	-0.40437	-1.57973
P	15.0	-2.08984	-0.67765	0.01922
O	8.0	-2.99818	0.10345	-1.02952
H	1.0	-3.08938	1.04445	-0.75659
O	8.0	-2.32975	-2.20390	-0.37508
H	1.0	-3.20409	-2.54693	-0.15316
O	8.0	-2.31396	-0.28808	1.43274
C	6.0	2.54894	0.33410	-0.00432
O	8.0	3.62562	0.62963	-0.67458
H	1.0	4.54536	-0.00933	-0.28457
O	8.0	2.43392	-0.52279	0.84528
O	8.0	-2.50102	2.41328	0.33243
H	1.0	-2.68126	1.95160	1.16504
H	1.0	-1.54992	2.28101	0.19164
O	8.0	5.06514	-0.93208	0.17224
H	1.0	4.46706	-1.27471	0.86351

Coordinate of reaction complex: GPS$(H_2O)_2$-C_3H+•OH

		X	y	Z
N	7.0	0.72476	0.34603	0.25505
H	1.0	0.61507	0.10451	1.23965
C	6.0	2.09176	0.75693	-0.00830
H	1.0	2.21832	0.96562	-1.07505
H	1.0	2.30663	1.69361	0.51817
C	6.0	0.29396	-0.80107	-0.55465
H	1.0	0.83262	-1.72650	-0.31460
H	1.0	0.42732	-0.56093	-1.61415
P	15.0	-1.47543	-1.05554	-0.23674
O	8.0	-2.29646	-0.10244	-1.19895
H	1.0	-2.17992	0.84503	-0.92990
O	8.0	-1.72003	-2.50225	-0.85976
H	1.0	-2.58190	-2.88371	-0.65283
O	8.0	-1.80787	-0.91300	1.20861
C	6.0	3.12684	-0.26174	0.43387
O	8.0	4.36136	0.08588	0.03795
H	1.0	4.98314	-0.58296	0.36281
O	8.0	2.88996	-1.25195	1.07792
O	8.0	-1.38113	2.19725	-0.18384
H	1.0	-1.85377	2.38102	0.64832
H	1.0	-0.56317	1.72248	0.09470
H	1.0	-2.20724	0.61241	2.07211
O	8.0	-2.26806	1.56058	2.30551
H	1.0	-2.83090	1.64240	3.07855
O	8.0	0.37367	1.99466	-2.26117
H	1.0	-0.39498	2.48355	-1.89776

Coordinate of product complex: GPS$(H_2O)_2$-C_3+H_2O

		X	y	Z
N	7.0	-0.56196	0.21186	-0.89918
H	1.0	0.05583	0.87005	-1.36507
C	6.0	-1.88421	0.44103	-1.05316
H	1.0	-1.85200	-0.27783	1.82560
H	1.0	-2.16526	1.29182	-1.65940
C	6.0	0.04200	-1.04210	-0.47770
H	1.0	-0.07132	-1.81339	-1.25187
H	1.0	-0.41640	-1.41143	0.44035
P	15.0	1.82129	-0.75976	-0.20946
O	8.0	2.08327	-0.38708	1.29170
H	1.0	1.69922	0.54303	1.51109
O	8.0	2.39050	-2.24707	-0.31950
H	1.0	3.20142	-2.39312	0.18303
O	8.0	2.33798	0.21634	-1.21192
C	6.0	-2.90253	-0.30922	-0.40080
O	8.0	-4.14367	-0.00123	-0.84752
H	1.0	-4.76774	-0.51621	-0.31751
O	8.0	-2.74422	-1.14557	0.49916
O	8.0	0.89759	1.81294	1.60895
H	1.0	0.90042	2.32126	0.77763
H	1.0	-0.00988	1.49983	1.79004
H	1.0	1.90609	1.93721	-1.22355
O	8.0	1.25620	2.65015	-1.04552
H	1.0	1.57476	3.46366	-1.44488
O	8.0	-1.28656	0.32937	2.33808
H	1.0	-1.30107	0.03742	3.25329

Coordinate of transition state: GPS$(H_2O)_2$-C_3—H--•OH

		X	y	Z
N	7.0	0.44015	-0.49335	1.23241
H	1.0	-0.13482	0.11187	1.80938
C	6.0	1.51462	0.19146	0.61683
H	1.0	1.27835	0.70303	-0.38103
H	1.0	1.85508	1.00292	1.26678
C	6.0	-0.29944	-1.50182	0.49260
H	1.0	-0.65454	-2.28170	1.17382
H	1.0	0.34674	-1.97812	-0.24767
P	15.0	-1.74907	-0.75573	-0.31148
O	8.0	-1.24488	0.13750	-1.50126
H	1.0	-1.10417	1.11633	-1.25204
O	8.0	-2.42645	-2.02572	-1.00369
H	1.0	-3.26609	-1.84207	-1.44304
O	8.0	-2.61538	-0.03674	0.67252
C	6.0	2.69947	-0.69981	0.30390
O	8.0	3.70381	0.03232	-0.23750
H	1.0	4.43815	-0.56812	-0.43465
O	8.0	2.77631	-1.88408	0.47530
O	8.0	-0.77128	2.51448	-0.70973
H	1.0	-0.90890	2.56678	0.25528
H	1.0	0.16231	2.68869	-0.90284
H	1.0	-2.02635	1.28106	1.59401
O	8.0	-1.36859	1.93704	1.92824
H	1.0	-1.78294	2.45339	2.62475
O	8.0	1.78723	1.81676	-1.51545
H	1.0	2.73808	1.66388	-1.36484

Coordinate of reaction complex: GPS$(H_2O)_2$-C_6H+•OH

		X	y	Z
N	7.0	-0.41248	-0.61992	-0.33654
H	1.0	-0.22303	-1.13168	0.52539
C	6.0	-1.75970	-0.86845	-0.79693
H	1.0	-1.95178	-0.30771	-1.71899
H	1.0	-1.88120	-1.92955	-1.04057
C	6.0	-0.05627	0.79062	-0.18288
H	1.0	-0.57161	1.29105	0.64826
H	1.0	-0.27672	1.33190	-1.10843
P	15.0	1.72926	0.88479	0.13228
O	8.0	2.48377	0.53673	-1.20794
H	1.0	2.46827	-0.45076	-1.39048
O	8.0	1.94572	2.45615	0.30181
H	1.0	2.77541	2.69106	0.73504
O	8.0	2.13791	0.06980	1.31295
C	6.0	-2.85588	-0.50161	0.18626
O	8.0	-4.06352	-0.83559	-0.28719
H	1.0	-4.73147	-0.58205	0.36753
O	8.0	-2.69107	0.02287	1.26267
O	8.0	1.92458	-1.99741	-1.45656
H	1.0	2.10521	-2.49500	-0.64118
H	1.0	0.98584	-1.75470	-1.35859
O	8.0	1.52204	-2.54450	1.24616
H	1.0	1.69698	-3.12315	1.99249
H	1.0	1.85610	-1.64933	1.47196
O	8.0	-2.56259	2.35241	-0.40471
H	1.0	-2.84717	2.17060	0.51292

Coordinate of product complex: GPS$(H_2O)_2$-C_6+H_2O

		X	y	Z
N	7.0	0.81128	0.16792	0.48531

Coordinate of transition state: GPS$(H_2O)_2$-C_6—H--•OH

		X	y	Z
N	7.0	-0.70478	0.40553	0.37382

H	1.0	0.33108	0.63691	1.24691
C	6.0	2.06934	0.70448	0.05422
H	1.0	2.01467	1.03398	-0.99368
H	1.0	2.32029	1.58186	0.65513
C	6.0	0.17374	-0.82494	-0.20221
H	1.0	0.73363	-1.38627	-0.93965
H	1.0	-0.56132	0.68061	-2.23886
P	15.0	-1.55112	-1.02628	0.01418
O	8.0	-2.35194	-0.21721	-1.12158
H	1.0	-2.43817	0.73577	-0.84073
O	8.0	-1.80048	-2.52847	-0.46097
H	1.0	-2.70942	-2.69231	-0.74199
O	8.0	-2.01845	-0.64887	1.38013
C	6.0	3.21456	-0.28778	0.16851
O	8.0	4.37822	0.30936	-0.15764
H	1.0	5.08024	-0.35566	-0.09827
O	8.0	3.12865	-1.44473	0.47634
O	8.0	-1.92705	2.26970	-0.32436
H	1.0	-1.70115	2.32080	0.62145
H	1.0	-1.10849	2.38851	-0.82995
O	8.0	-1.12997	1.65475	2.28400
H	1.0	-1.27227	1.97384	3.17869
H	1.0	-1.59305	0.78867	2.17876
O	8.0	-0.04289	1.49752	-2.29320
H	1.0	-0.14276	1.82501	-3.19247

H	1.0	-0.58215	1.23250	-0.21278
C	6.0	-2.07567	0.24859	0.80898
H	1.0	-2.14630	-0.61540	1.47923
H	1.0	-2.38828	1.12583	1.38525
C	6.0	-0.15201	-0.77354	-0.24926
H	1.0	-0.70735	-1.16833	-1.11127
H	1.0	-0.17768	-1.63370	0.56205
P	15.0	1.60708	-0.52821	-0.60991
O	8.0	2.33989	-0.60213	0.78743
H	1.0	2.18674	0.24527	1.32691
O	8.0	2.01813	-1.89254	-1.32316
H	1.0	2.62140	-1.76187	-2.06528
O	8.0	1.88107	0.69268	-1.41907
C	6.0	-3.06949	0.07318	-0.32759
O	8.0	-4.30522	-0.17117	0.13746
H	1.0	-4.90096	-0.26131	-0.62174
O	8.0	-2.80549	0.15543	-1.49957
O	8.0	1.50371	1.50777	1.97778
H	1.0	1.56966	2.28761	1.39753
H	1.0	0.58274	1.21709	1.85285
O	8.0	0.99384	2.98449	-0.31163
H	1.0	1.05272	3.84033	-0.74382
H	1.0	1.41375	2.31757	-0.89458
O	8.0	0.01536	-2.29621	1.76361
H	1.0	0.95571	-2.05711	1.88213

Coordinate of reaction complex: GPS$(H_2O)_2$-N_1H+$^•$OH

		X	y	Z
N	7.0	-0.72038	0.18600	0.23759
H	1.0	-0.65836	0.58392	-0.69793
C	6.0	-2.06551	0.37135	0.74152
H	1.0	-2.15143	-0.03671	1.75411
H	1.0	-2.27976	1.44389	0.80495
C	6.0	-0.27229	-1.20815	0.19327
H	1.0	-0.75079	-1.80063	-0.59648
H	1.0	-0.45972	-1.68663	1.15990
P	15.0	1.52083	-1.16565	-0.06605
O	8.0	2.20438	-0.83166	1.31333
H	1.0	2.08208	0.14247	1.55329
O	8.0	1.86207	-2.70419	-0.30989
H	1.0	2.75990	-2.86949	-0.62241
O	8.0	1.90898	-0.25716	-1.18769
C	6.0	-3.14044	-0.25681	-0.12765
O	8.0	-4.34874	-0.18602	0.45580
H	1.0	-4.99950	-0.57310	-0.14921
O	8.0	-2.95642	-0.74717	-1.21238
O	8.0	1.54071	1.63689	1.64335
H	1.0	1.94805	2.19060	0.95716
H	1.0	0.61796	1.51604	1.35555
H	1.0	2.10916	1.39039	-1.18510
O	8.0	2.09749	2.36829	-1.01595
H	1.0	2.72784	2.78252	-1.61141
O	8.0	-0.56067	2.83464	-0.40533
H	1.0	0.35213	2.87312	-0.78248

Coordinate of product complex: GPS$(H_2O)_2$-N_1+H_2O

		X	y	Z
N	7.0	0.87557	-0.29807	-0.81094
H	1.0	-0.33881	-1.82513	-1.19257
C	6.0	1.78123	-0.75121	0.21171
H	1.0	1.49942	-0.35046	1.20014
H	1.0	1.76202	-1.84301	0.27399
C	6.0	0.63017	1.11317	-0.78766
H	1.0	0.50162	1.48826	-1.80761
H	1.0	1.38628	1.71059	-0.26335
P	15.0	-0.97893	1.35694	0.05680
O	8.0	-0.80568	0.73398	1.48145
H	1.0	-1.19839	-0.22809	1.58258
O	8.0	-0.95733	2.94255	0.26129
H	1.0	-1.83872	3.33606	0.27829
O	8.0	-2.15780	0.87570	-0.71904
C	6.0	3.21400	-0.31104	-0.05550
O	8.0	4.05868	-0.93614	0.77935
H	1.0	4.95586	-0.62263	0.58889
O	8.0	3.56287	0.48718	-0.88377
O	8.0	-1.70748	-1.59203	1.57053
H	1.0	-2.63221	-1.59964	1.27292
H	1.0	-1.25055	-2.08887	0.85158
H	1.0	-3.11905	-0.55896	-0.72447
O	8.0	-3.48157	-1.46331	-0.59203
H	1.0	-4.37049	-1.48071	-0.95563
O	8.0	-0.85990	-2.55666	-0.81733
H	1.0	-1.76874	-2.44068	-1.13526

Coordinate of transition state: GPS$(H_2O)_2$-N_1—H--$^•$OH

		X	y	Z
N	7.0	-0.83326	-0.80629	1.27502
H	1.0	-0.41298	0.11002	1.58438
C	6.0	-2.24880	-0.71694	1.06423
H	1.0	-2.70379	-1.71363	1.12782
H	1.0	-2.69423	-0.09960	1.84920
C	6.0	-0.08649	-1.65739	0.37500
H	1.0	-0.67593	-1.94542	-0.50292
H	1.0	0.22037	-2.57246	0.90038
P	15.0	1.45004	-0.92702	-0.27811
O	8.0	2.21325	-0.34000	0.95551
H	1.0	2.29889	0.68532	0.95856
O	8.0	2.20410	-2.28435	-0.67647
H	1.0	2.82403	-2.16176	-1.40622
O	8.0	1.30213	-0.00154	-1.43580
C	6.0	-2.65171	-0.10863	-0.27393
O	8.0	-3.98354	-0.16354	-0.43930
H	1.0	-4.20072	0.25117	-1.28781
O	8.0	-1.89965	0.36423	-1.08553
O	8.0	2.17672	2.18952	0.99219
H	1.0	1.99633	2.54348	0.10423
H	1.0	1.30569	2.24221	1.42588

Coordinate of reaction complex: GPS$(H_2O)_2$-O_{16}H+$^•$OH

		X	y	Z
N	7.0	0.14564	0.61846	-0.51333
H	1.0	0.12685	0.91776	0.46260
C	6.0	1.43514	0.91038	-1.09918
H	1.0	1.45867	0.58832	-2.14543
H	1.0	1.60835	1.99269	-1.10171
C	6.0	-0.23701	-0.79173	-0.61074
H	1.0	0.42602	-1.46724	-0.05332
H	1.0	-0.25023	-1.09362	-1.66322
P	15.0	-1.91852	-0.97640	0.04820
O	8.0	-2.92030	-0.34542	-0.99206
H	1.0	-2.89274	0.66054	-0.96539
O	8.0	-2.14606	-2.54910	-0.10060
H	1.0	-2.86829	-2.89170	0.44009
O	8.0	-2.04395	-0.45816	1.44039
C	6.0	2.61278	0.27941	-0.37485
O	8.0	3.75027	0.42701	-1.04767
H	1.0	4.48983	0.04155	-0.53373
O	8.0	2.52405	-0.27112	0.70359
O	8.0	-2.32120	2.17650	-0.82863
H	1.0	-2.31244	2.48453	0.09403
H	1.0	-1.38910	1.94347	-0.99051

H	1.0	0.70192	1.59516	-1.52677
O	8.0	0.49667	2.51054	-1.23002
H	1.0	0.09135	2.97899	-1.96386
O	8.0	-0.53677	1.57192	1.25679
H	1.0	-0.52994	1.75946	0.29297

O	8.0	-1.41177	2.13308	1.79830
H	1.0	-1.41643	2.54574	2.66549
H	1.0	-1.70830	1.20354	1.90251
O	8.0	5.23317	-0.87693	1.01605
H	1.0	4.30686	-0.88400	1.35259

Coordinate of product complex: GPS$(H_2O)_2$-O_{16}+H_2O

		X	y	Z
N	7.0	0.27683	0.36100	0.45143
H	1.0	-0.00982	1.02198	1.16824
C	6.0	1.13841	0.79275	-0.54678
H	1.0	0.89021	0.36231	-1.52607
H	1.0	1.16148	1.87744	-0.59313
C	6.0	-0.21186	-0.99246	0.53178
H	1.0	-0.15869	-1.37412	1.55551
H	1.0	0.38600	-1.63534	-0.11855
P	15.0	-1.95700	-0.96483	-0.00414
O	8.0	-1.93664	-0.37990	-1.46849
H	1.0	-1.89109	0.61635	-1.48082
O	8.0	-2.29818	-2.50617	-0.18490
H	1.0	-3.22851	-2.72720	-0.04942
O	8.0	-2.83834	-0.23600	0.95354
C	6.0	2.56487	0.24685	-0.32141
O	8.0	3.38430	1.16162	-0.36879
H	1.0	5.26770	0.12573	0.27897
O	8.0	2.61244	-0.97118	-0.16830
O	8.0	-1.62993	2.22639	-1.19092
H	1.0	-1.74321	2.45028	-0.24511
H	1.0	-2.07638	2.89795	-1.71499
O	8.0	-1.78776	2.13861	1.54987
H	1.0	-2.08183	2.71008	2.26482
H	1.0	-2.35332	1.33232	1.53320
O	8.0	5.45387	-0.77722	0.56165
H	1.0	4.62813	-1.24434	0.37863

Coordinate of transition state: GPS$(H_2O)_2$-O_{16}—H--•OH

		X	y	Z
N	7.0	0.17944	0.55782	-0.49750
H	1.0	0.09772	0.96053	0.43724
C	6.0	1.47596	0.80524	-1.05754
H	1.0	1.54258	0.40559	-2.07305
H	1.0	1.67299	1.88042	-1.10570
C	6.0	-0.22767	-0.84540	-0.49834
H	1.0	0.39223	-1.47571	0.15398
H	1.0	-0.18419	-1.23814	-1.51958
P	15.0	-1.94670	-0.95275	0.07799
O	8.0	-2.87731	-0.37947	-1.05798
H	1.0	-2.85332	0.62388	-1.07970
O	8.0	-2.19424	-2.52703	0.02286
H	1.0	-2.94829	-2.82604	0.54604
O	8.0	-2.13628	-0.33374	1.42042
C	6.0	2.64094	0.18835	-0.22471
O	8.0	3.76679	0.27714	-0.87223
H	1.0	4.65454	-0.08927	-0.19141
O	8.0	2.47433	-0.27887	0.88085
O	8.0	-2.28594	2.16056	-0.99340
H	1.0	-2.26808	2.49768	-0.08076
H	1.0	-1.35472	1.94786	-1.16829
O	8.0	-1.37642	2.23104	1.63406
H	1.0	-1.34527	2.70684	2.46789
H	1.0	-1.72720	1.33115	1.80786
O	8.0	5.17920	-0.70229	0.64497
H	1.0	4.52909	-0.80609	1.36526

Coordinate of reaction complex: GPS$(H_2O)_3$-C_3H+•OH

		X	y	Z
N	7.0	1.01486	0.27817	0.28704
H	1.0	0.73362	-0.17255	1.15792
C	6.0	2.46072	0.24124	0.15520
H	1.0	2.75753	0.65238	-0.81465
H	1.0	2.92087	0.88099	0.91690
C	6.0	0.31147	-0.37759	-0.82266
H	1.0	0.51437	-1.45537	-0.88079
H	1.0	0.60464	0.09914	-1.76370
P	15.0	-1.47944	-0.15192	-0.58560
O	8.0	-1.85689	1.27872	-1.12508
H	1.0	-1.38276	1.99597	-0.62350
O	8.0	-2.19324	-1.11877	-1.60704
H	1.0	-2.62614	-1.87607	-1.14755
O	8.0	-1.82944	-0.41467	0.85442
C	6.0	3.04310	-1.15081	0.31935
O	8.0	4.35246	-1.17754	0.02507
H	1.0	4.67716	-2.07821	0.17598
O	8.0	2.42615	-2.11658	0.69088
O	8.0	-0.26180	2.75179	0.40507
H	1.0	-0.69246	2.84352	1.27272
H	1.0	0.31992	1.95598	0.50939
H	1.0	-1.65007	0.85236	2.12678
O	8.0	-1.35788	1.63341	2.63831
H	1.0	-1.87365	1.66705	3.44730
O	8.0	-3.08332	-2.80374	0.27942
H	1.0	-3.94507	-3.13628	0.54275
H	1.0	-2.80573	-2.12030	0.91455
O	8.0	1.51976	2.56423	-1.63767
H	1.0	0.91715	3.17113	-1.15796

Coordinate of product complex: GPS$(H_2O)_3$-C_3+H_2O

		X	y	Z
N	7.0	-0.75579	-0.15538	-0.94794
H	1.0	-0.19073	0.40528	-1.57870
C	6.0	-2.07091	-0.21765	-1.25569
H	1.0	-2.41235	0.08260	1.71443
H	1.0	-2.39084	0.33113	-2.13165
C	6.0	-0.03168	-1.06987	-0.07999
H	1.0	0.21508	-1.99989	-0.61113
H	1.0	-0.63066	-1.32579	0.79427
P	15.0	1.53458	-0.28833	0.42929
O	8.0	1.23287	0.69558	1.60153
H	1.0	0.67927	1.51577	1.31107
O	8.0	2.37329	-1.45630	1.07875
H	1.0	3.21187	-1.62603	0.58744
O	8.0	2.19314	0.32845	-0.78059
C	6.0	-3.05158	-0.88225	-0.46565
O	8.0	-4.26117	-0.92764	-1.07516
H	1.0	-4.86995	-1.34996	-0.45322
O	8.0	-2.89561	-1.36453	0.66350
O	8.0	-0.24428	2.56554	0.78221
H	1.0	-0.13574	2.67373	-0.17864
H	1.0	-1.10952	2.15533	0.97301
H	1.0	1.46937	1.74943	-1.57677
O	8.0	0.69694	2.28651	-1.85621
H	1.0	0.98668	2.92579	-2.51229
O	8.0	4.36370	-1.36764	-0.71632
H	1.0	5.29010	-1.12898	-0.62769
H	1.0	3.88216	-0.60097	-1.07534
O	8.0	-2.08935	0.95901	1.98784
H	1.0	-1.75413	0.87198	2.88499

Coordinate of transition state: GPS$(H_2O)_3$-C_3—H--•OH

		X	y	Z
N	7.0	-0.85855	0.06631	-0.63767
H	1.0	-0.52990	-0.14653	-1.57479
C	6.0	-2.27806	0.21520	-0.54730
H	1.0	-2.51866	0.78294	0.47312
H	1.0	-2.65999	0.87487	-1.33139
C	6.0	-0.22554	-0.79495	0.36647
H	1.0	-0.25287	-1.85698	0.10125
H	1.0	-0.74318	-0.66462	1.32256
P	15.0	1.50507	-0.26565	0.57330
O	8.0	1.50880	0.94357	1.58563

Coordinate of reaction complex: GPS$(H_2O)_3$-C_6H+•OH

		X	y	Z
N	7.0	-0.54573	-1.01119	-0.23544
H	1.0	-0.45285	-0.84476	0.76724
C	6.0	-1.92270	-1.30846	-0.56494
H	1.0	-2.02574	-1.48317	-1.64082
H	1.0	-2.23569	-2.23307	-0.06609
C	6.0	0.00317	0.12519	-0.97824
H	1.0	-0.50771	1.07458	-0.76539
H	1.0	-0.06222	-0.07624	-2.05290
P	15.0	1.75859	0.33592	-0.55482
O	8.0	2.55299	-0.84630	-1.21486

H	1.0	0.98349	1.71466	1.25212
O	8.0	2.22701	-1.40812	1.38693
H	1.0	2.87943	-1.89555	0.83222
O	8.0	2.12149	0.01231	-0.77098
C	6.0	-3.09687	-1.05537	-0.49454
O	8.0	-4.40007	-0.78497	-0.33942
H	1.0	-4.88156	-1.62506	-0.30011
O	8.0	-2.64900	-2.16989	-0.58702
O	8.0	-0.09185	2.60861	0.21580
H	1.0	0.47250	3.10510	-0.40068
H	1.0	-0.35595	1.81468	-0.30485
H	1.0	1.96673	1.63036	-1.57616
O	8.0	1.66899	2.51530	-1.86692
H	1.0	2.35803	2.88671	-2.42262
O	8.0	3.73977	-2.21448	-0.68161
H	1.0	4.67853	-2.32582	-0.85139
H	1.0	3.43715	-1.39999	-1.12012
O	8.0	-2.38055	1.52534	1.62034
H	1.0	-1.75372	2.21520	1.31524

H	1.0	2.33647	-1.74071	-0.79655
O	8.0	2.21750	1.61600	-1.35622
H	1.0	2.57547	2.31660	-0.76122
O	8.0	1.92103	0.46819	0.93695
C	6.0	-2.91354	-0.23157	-0.15550
O	8.0	-4.12494	-0.45114	-0.65804
H	1.0	-4.74087	0.24550	-0.34981
O	8.0	-2.63284	0.70398	0.56559
O	8.0	1.60350	-2.94627	-0.04769
H	1.0	1.70316	-2.85934	0.91629
H	1.0	0.69847	-2.61612	-0.20519
H	1.0	1.53671	-0.84386	2.04715
O	8.0	1.17268	-1.69030	2.38336
H	1.0	1.29320	-1.70983	3.33593
O	8.0	2.96202	3.01524	0.81060
H	1.0	3.82375	3.30034	1.12539
H	1.0	2.70924	2.20936	1.29605
O	8.0	-5.17054	1.87181	0.63166
H	1.0	-4.22287	1.85952	0.90157

Coordinate of product complex: GPS$(H_2O)_3$-C_6+H_2O

		X	y	Z
N	7.0	1.04014	0.14486	0.33612
H	1.0	0.65166	0.28526	1.26345
C	6.0	2.45669	0.27001	0.17150
H	1.0	2.68832	0.84667	-0.73378
H	1.0	2.88007	0.82576	1.01256
C	6.0	0.23133	-0.30620	-0.66916
H	1.0	0.69105	-0.68987	-1.57258
H	1.0	0.36560	1.89975	-1.75005
P	15.0	-1.51797	-0.17878	-0.49483
O	8.0	-1.98799	1.24290	-1.01998
H	1.0	-1.70421	1.96681	-0.38756
O	8.0	-2.15183	-1.14898	-1.56733
H	1.0	-2.50424	-1.96540	-1.14523
O	8.0	-1.94167	-0.44859	0.92873
C	6.0	3.17954	-1.06639	0.08091
O	8.0	4.50754	-0.86835	-0.03872
H	1.0	4.93387	-1.73615	-0.10067
O	8.0	2.67112	-2.15320	0.10011
O	8.0	-0.85224	2.92212	0.64285
H	1.0	-0.71568	2.49063	1.50503
H	1.0	0.00983	3.00197	0.20420
H	1.0	-1.26792	0.43808	2.22847
O	8.0	-0.62885	1.03723	2.68425
H	1.0	-0.78552	0.99511	3.63089
O	8.0	-2.99438	-2.92531	0.28254
H	1.0	-3.87378	-3.25231	0.48801
H	1.0	-2.77943	-2.21355	0.91156
O	8.0	0.97422	2.60650	-1.48822
H	1.0	0.92631	3.27576	-2.17824

Coordinate of transition state: GPS$(H_2O)_3$-C_6—H--•OH

		X	y	Z
N	7.0	-1.09634	0.52380	-0.19715
H	1.0	-0.86070	0.37772	-1.17936
C	6.0	-2.52434	0.47103	0.02875
H	1.0	-2.73235	0.67712	1.08445
H	1.0	-3.02571	1.25235	-0.55247
C	6.0	-0.33755	-0.35296	0.66253
H	1.0	-0.63781	-1.41005	0.65455
H	1.0	-0.52043	0.01395	1.77090
P	15.0	1.44630	-0.15041	0.39614
O	8.0	1.82028	1.22884	1.06057
H	1.0	1.43257	2.01795	0.54622
O	8.0	2.12943	-1.22333	1.32531
H	1.0	2.67755	-1.86353	0.81073
O	8.0	1.80247	-0.28151	-1.05905
C	6.0	-3.16148	-0.85583	-0.35062
O	8.0	-4.45991	-0.88873	-0.00975
H	1.0	-4.82316	-1.74450	-0.28396
O	8.0	-2.59635	-1.76442	-0.90308
O	8.0	0.51651	2.95847	-0.29689
H	1.0	0.69158	2.83215	-1.24714
H	1.0	-0.31381	2.46729	-0.15733
H	1.0	1.18823	0.83908	-2.28357
O	8.0	0.63940	1.55211	-2.67238
H	1.0	0.82340	1.59509	-3.61409
O	8.0	3.33856	-2.54395	-0.65940
H	1.0	4.25855	-2.69681	-0.88988
H	1.0	2.98674	-1.84642	-1.23963
O	8.0	-0.60494	0.86928	2.86294
H	1.0	0.29184	1.25066	2.78841

Coordinate of reaction complex: GPS$(H_2O)_3$-N_1H+•OH

		X	y	Z
N	7.0	1.03768	0.26043	-0.23749
H	1.0	0.89680	0.34633	0.76780
C	6.0	2.46043	0.20598	-0.50993
H	1.0	2.63656	0.11145	-1.58646
H	1.0	2.92345	1.14440	-0.18507
C	6.0	0.28265	-0.89793	-0.72271
H	1.0	0.49042	-1.81868	-0.16166
H	1.0	0.51329	-1.07219	-1.77895
P	15.0	-1.49039	-0.51657	-0.59443
O	8.0	-1.86321	0.41595	-1.79882
H	1.0	-1.40167	1.31529	-1.73786
O	8.0	-2.25747	-1.86028	-0.90444
H	1.0	-2.68678	-2.23398	-0.09983
O	8.0	-1.79222	0.04490	0.77462
C	6.0	3.17800	-0.92502	0.20489
O	8.0	4.45167	-1.04011	-0.20700
H	1.0	4.86970	-1.75063	0.30258
O	8.0	2.68958	-1.62889	1.05134
O	8.0	-0.47089	2.53000	-1.34140
H	1.0	-0.85099	2.98808	-0.57491
H	1.0	0.29385	2.03610	-0.98590
H	1.0	-1.60515	1.65895	1.20646
O	8.0	-1.35267	2.60719	1.34307
H	1.0	-2.00435	3.00892	1.92393

Coordinate of product complex: GPS$(H_2O)_3$-N_1+H_2O

		X	y	Z
N	7.0	-1.14642	0.10703	-0.80020
H	1.0	-0.52578	1.89357	-1.34190
C	6.0	-2.23256	0.35020	0.11285
H	1.0	-1.93616	0.14165	1.15455
H	1.0	-2.54695	1.39637	0.05497
C	6.0	-0.48243	-1.14267	-0.59106
H	1.0	-0.15446	-1.55966	-1.54860
H	1.0	-1.06429	-1.89207	-0.04004
P	15.0	1.04933	-0.80886	0.36989
O	8.0	0.57280	-0.10681	1.67965
H	1.0	0.58101	0.93376	1.64717
O	8.0	1.53665	-2.24716	0.80746
H	1.0	2.38764	-2.49745	0.37712
O	8.0	2.07046	-0.05807	-0.44089
C	6.0	-3.43770	-0.53043	-0.18818
O	8.0	-4.50607	-0.11429	0.50972
H	1.0	-5.24660	-0.70643	0.30851
O	8.0	-3.45198	-1.47524	-0.93098
O	8.0	0.58403	2.38433	1.46538
H	1.0	1.47781	2.68370	1.23419
H	1.0	0.06362	2.62734	0.66283
H	1.0	2.42951	1.63428	-0.60539
O	8.0	2.47795	2.61600	-0.59172
H	1.0	3.34343	2.87354	-0.91921

O	8.0	-3.10120	-2.23480	1.61389
H	1.0	-3.95304	-2.34160	2.04459
H	1.0	-2.78195	-1.32964	1.77407
O	8.0	1.41490	2.52396	1.25913
H	1.0	0.47752	2.70115	1.51337

O	8.0	3.74002	-2.24461	-0.71989
H	1.0	4.68255	-2.32260	-0.55173
H	1.0	3.52711	-1.30882	-0.87946
O	8.0	-0.32491	2.80182	-1.05184
H	1.0	0.59114	2.97623	-1.31664

Coordinate of transition state: GPS$(H_2O)_3$-N_1—H--•OH

		X	y	Z
N	7.0	1.13171	-0.77016	-1.36464
H	1.0	0.94195	0.22008	-1.45577
C	6.0	2.45364	-1.04543	-0.88285
H	1.0	2.71136	-2.09807	-1.04890
H	1.0	3.18403	-0.45003	-1.44132
C	6.0	0.05968	-1.56070	-0.78444
H	1.0	0.34874	-2.01991	0.17067
H	1.0	-0.25565	-2.36370	-1.46291
P	15.0	-1.41571	-0.58264	-0.40239
O	8.0	-1.68792	0.31983	-1.66557
H	1.0	-1.42420	1.27965	-1.53358
O	8.0	-2.54472	-1.68902	-0.37182
H	1.0	-3.24887	-1.48102	0.28327
O	8.0	-1.34090	0.21445	0.87287
C	6.0	2.67440	-0.73667	0.59221
O	8.0	3.86270	-1.19360	1.01955
H	1.0	3.96784	-0.94126	1.94914
O	8.0	1.90063	-0.14777	1.30643
O	8.0	-0.75706	2.70341	-1.18540
H	1.0	-0.76014	2.86818	-0.22804
H	1.0	0.18199	2.70470	-1.42260
H	1.0	-0.27882	1.43248	1.37561
O	8.0	0.19387	2.29903	1.43773
H	1.0	0.42549	2.43743	2.36013
O	8.0	-3.83891	-0.62457	1.74153
H	1.0	-4.60552	-0.05656	1.85320
H	1.0	-3.03848	-0.07562	1.81547
O	8.0	2.02084	1.95717	-0.58904
H	1.0	1.56932	2.01960	0.29418

Coordinate of reaction complex: GPS$(H_2O)_3$-O_{16}H+•OH

		X	y	Z
N	7.0	-0.54712	-1.01032	-0.23642
H	1.0	-0.45411	-0.84497	0.76642
C	6.0	-1.92422	-1.30663	-0.56614
H	1.0	-2.02743	-1.48020	-1.64220
H	1.0	-2.23761	-2.23161	-0.06821
C	6.0	0.00229	0.12644	-0.97821
H	1.0	-0.50808	1.07591	-0.76461
H	1.0	-0.06311	-0.07404	-2.05304
P	15.0	1.75782	0.33587	-0.55475
O	8.0	2.55159	-0.84624	-1.21601
H	1.0	2.33563	-1.74058	-0.79773
O	8.0	2.21655	1.61655	-1.35531
H	1.0	2.58076	2.31420	-0.76072
O	8.0	1.92111	0.46660	0.93703
C	6.0	-2.91460	-0.22968	-0.15560
O	8.0	-4.12618	-0.44839	-0.65806
H	1.0	-4.74177	0.24828	-0.34924
O	8.0	-2.63338	0.70516	0.56618
O	8.0	1.60215	-2.94664	-0.04806
H	1.0	1.70041	-2.85913	0.91601
H	1.0	0.69726	-2.61700	-0.20693
H	1.0	1.53441	-0.84530	2.04729
O	8.0	1.16850	-1.69119	2.38272
H	1.0	1.28809	-1.71152	3.33538
O	8.0	2.97561	3.00835	0.81140
H	1.0	3.83837	3.29000	1.12643
H	1.0	2.71932	2.20363	1.29688
O	8.0	-5.17063	1.87386	0.63351
H	1.0	-4.22285	1.86131	0.90305

Coordinate of product complex: GPS$(H_2O)_3$-O_{16}+H_2O

		X	y	Z
N	7.0	-1.25008	-0.15487	-1.47631
H	1.0	-1.83074	-0.87047	-1.02235
C	6.0	-1.78154	1.13202	-1.59159
H	1.0	-1.40481	1.64031	-2.48448
H	1.0	-2.86840	1.08911	-1.57453
C	6.0	0.15703	-0.42883	-1.61092
H	1.0	0.69051	0.50281	-1.81740
H	1.0	0.33043	-1.14302	-2.42648
P	15.0	0.84313	-1.20194	-0.07837
O	8.0	0.25695	-2.67135	-0.08439
H	1.0	-0.68625	-2.69083	0.19701
O	8.0	2.35094	-1.40561	-0.38327
H	1.0	2.95758	-0.57709	-0.21360
O	8.0	0.47007	-0.39229	1.12222
C	6.0	-1.28640	1.97433	-0.42773
O	8.0	-0.14496	2.41346	-0.59768
H	1.0	1.26798	2.50241	0.68044
O	8.0	-2.10982	2.05776	0.48704
O	8.0	-2.36046	-2.07343	0.34288
H	1.0	-2.39081	-1.39700	1.10016
H	1.0	-3.10370	-2.67697	0.43296
H	1.0	-1.13183	-0.25195	2.06945
O	8.0	-2.10686	-0.25409	2.10858
H	1.0	-2.35954	0.62141	1.77018
O	8.0	3.65862	0.67882	-0.00039
H	1.0	3.05729	1.31585	0.50220
H	1.0	4.53107	0.68100	0.40293
O	8.0	1.88005	2.02703	1.26974
H	1.0	1.35413	1.26006	1.55686

Coordinate of transition state: GPS$(H_2O)_3$-O_{16}—H--•OH

		X	y	Z
N	7.0	0.59421	0.92244	-0.28109
H	1.0	0.47720	0.86820	0.73172
C	6.0	1.96868	1.14528	-0.62696
H	1.0	2.08861	1.21410	-1.71110
H	1.0	2.32858	2.08205	-0.18952
C	6.0	-0.01410	-0.23542	-0.93296
H	1.0	0.43555	-1.18949	-0.62249
H	1.0	0.08545	-0.13670	-2.01914
P	15.0	-1.78812	-0.31237	-0.53839
O	8.0	-2.48976	0.88400	-1.27575
H	1.0	-2.25874	1.77752	-0.87326
O	8.0	-2.30286	-1.59850	-1.29250
H	1.0	-2.73283	-2.24022	-0.67811
O	8.0	-1.99474	-0.35952	0.95188
C	6.0	2.92613	0.03655	-0.09618
O	8.0	4.11078	0.15889	-0.62326
H	1.0	4.81863	-0.69689	-0.23295
O	8.0	2.57478	-0.78946	0.71732
O	8.0	-1.49707	2.99349	-0.13095
H	1.0	-1.56039	2.90318	0.83600
H	1.0	-0.59512	2.68514	-0.32198
H	1.0	-1.44886	0.92674	2.01833
O	8.0	-0.98869	1.74911	2.29200
H	1.0	-1.00535	1.80006	3.25103
O	8.0	-3.21343	-2.83424	0.90013
H	1.0	-4.08922	-3.07771	1.21065
H	1.0	-2.93101	-2.03037	1.37106
O	8.0	5.21679	-1.51091	0.49311
H	1.0	4.43915	-1.87847	0.95401

Table 9s Harmonic frequencies of reaction complexes, transition states, and product complexes of the reaction processes of glyphosate and its hydrates with the •OH radical

GPS-C_3H+•OH

3891.0181	3888.1210	3825.8409	3724.7582	3552.8390	3130.2483
3124.0032	3078.3087	3054.6036	1890.9118	1513.1165	1471.9992
1456.0644	1406.9726	1341.7306	1310.9996	1304.8451	1287.8290
1235.9831	1187.2873	1166.7412	1090.1629	1024.0489	989.8430

GPS-C_3+H_2O

3989.0510	3890.6456	3885.7903	3848.1563	3819.2190	3603.5308
3267.4654	3184.7019	3060.8887	1774.7114	1624.0767	1601.6921
1539.4126	1467.3819	1349.7575	1325.5025	1308.9714	1284.0544
1212.2670	1161.3556	1087.3971	1021.9390	991.4790	956.8187

975.2834 948.2852 909.0246 891.6951 863.6051 792.0326<br>767.8643 663.1187 631.1104 541.3163 492.8587 465.9026<br>464.5855 429.9416 407.0513 388.4069 356.4632 301.4418<br>296.0722 292.2128 258.7660 190.9347 160.4153 130.7917<br>121.8634 85.5994 65.1164 54.3127 33.1571 31.7841	944.1048 889.9368 872.1004 807.6234 784.0474 701.7245<br>695.4098 585.9739 554.7965 476.4126 473.4775 442.1410<br>432.9073 425.2630 370.5306 365.9563 321.9142 286.2463<br>275.5093 254.6054 199.7208 187.5008 185.0296 168.6480<br>135.9674 99.4187 65.4267 48.5749 26.8762 22.0723
GPS-C_3—H--$^{\bullet}$OH<br>3897.7003 3895.3090 3819.7762 3754.6016 3586.6731 3131.2647<br>3122.2421 3065.3340 1885.8471 1592.7943 1490.2925 1469.6738<br>1399.8360 1345.8232 1333.0602 1309.6537 1303.8520 1274.3931<br>1212.7521 1179.7814 1138.7276 1068.7636 1015.3197 992.9126<br>959.8956 949.8766 901.5027 884.0428 868.5604 793.1756<br>707.9770 676.3560 656.9496 583.8971 528.7442 484.7492<br>455.5427 434.7498 421.5008 353.9117 311.1096 298.1291<br>296.9815 287.8469 231.8107 216.8665 142.4330 129.2691<br>100.2445 68.2494 58.1208 36.0912 30.5658 -885.1974	GPS-C_6H+$^{\bullet}$OH<br>3893.4642 3887.3744 3625.5225 3572.7408 3553.6159 3126.8862<br>3124.8137 3081.9384 3056.1625 1829.1159 1511.9020 1468.9818<br>1447.9343 1441.5487 1343.6836 1339.0333 1307.7696 1285.7169<br>1252.2293 1225.5068 1182.2453 1089.6162 1022.5624 996.4292<br>970.7371 948.7876 912.4115 898.3470 874.3964 791.6397<br>760.1032 752.3035 675.6328 608.8329 549.5803 543.9688<br>467.3059 431.3144 424.0292 393.3864 361.6162 319.6000<br>301.5546 289.1660 259.9340 203.1731 184.1065 159.4907<br>122.8714 100.4824 83.5734 51.4022 44.2551 28.1543
GPS-C_6+H_2O<br>3967.8334 3895.3839 3867.4514 3814.6421 3806.6758 3617.7622<br>3263.6038 3144.9990 3069.3744 1891.9205 1606.5928 1582.3950<br>1462.0842 1439.3660 1375.2806 1324.2670 1307.5754 1291.1635<br>1248.8566 1179.7754 1115.8789 1030.2054 1009.3512 994.7967<br>928.1614 890.8966 858.4522 825.9699 692.0466 664.8332<br>589.7868 568.6816 555.4898 518.8655 465.6282 451.9266<br>423.5994 394.9342 364.3027 314.4322 310.3509 292.7945<br>269.9781 265.0753 194.2102 174.0443 149.4358 132.9041<br>119.4505 105.8057 80.0815 50.5655 45.0227 39.8585	GPS-C_6—H--$^{\bullet}$OH<br>3891.8461 3887.8123 3826.4719 3759.0755 3563.3194 3136.7681<br>3087.7175 3070.6525 1875.6406 1498.3359 1481.2480 1449.3663<br>1423.7713 1342.2654 1316.4855 1309.2498 1294.0094 1255.9730<br>1221.7297 1172.4965 1145.2226 1109.2868 1019.5517 1006.5640<br>984.2529 929.2471 925.7862 880.0361 872.3068 791.6472<br>736.2109 675.6715 656.9349 595.7162 539.9276 506.1257<br>459.2635 425.6427 409.5620 382.8051 348.6397 295.6974<br>290.2262 263.9354 225.1922 185.5535 164.8514 120.6483<br>108.4347 97.8546 58.5924 53.6921 45.5727 -1145.1937
GPS-N_1H+$^{\bullet}$OH<br>3876.8001 3834.3490 3750.2658 3638.4132 3564.3829 3158.5006<br>3144.9977 3091.2215 3038.5558 1885.4583 1502.3857 1463.0564<br>1441.1528 1438.7395 1323.8614 1312.0695 1296.7816 1260.7829<br>1237.0622 1206.8952 1163.2642 1064.0818 1052.3151 1000.6971<br>982.6190 959.7743 905.7436 882.9145 855.0252 749.2218<br>688.0274 683.0241 643.7286 571.3653 522.4246 513.1958<br>477.3321 468.3493 446.9491 399.9217 383.2487 350.5976<br>320.9530 265.8501 243.5587 213.6161 180.0163 159.1269<br>150.4797 125.9513 74.6716 63.0027 42.4271 39.7664	GPS-N_1+H_2O<br>3882.3918 3857.6484 3822.9337 3776.5981 3491.5002 3143.7740<br>3131.5910 3060.4218 3024.6592 1877.7125 1650.8795 1467.8462<br>1451.5342 1400.6526 1309.1702 1308.1602 1259.1049 1244.8730<br>1206.3065 1176.1470 1155.4931 1141.4827 1010.2093 1001.5959<br>970.8047 967.2750 908.0873 867.7426 853.2516 822.8501<br>732.7839 672.3161 654.0508 629.1000 529.9458 518.1969<br>479.1387 462.3315 446.5660 401.6197 374.2169 328.7057<br>312.6976 263.0154 249.8283 213.4293 204.7314 163.8507<br>147.2857 110.0072 87.5301 69.2111 43.1521 38.5347
GPS-N_1—H--$^{\bullet}$OH<br>3886.5795 3819.6318 3725.8280 3451.7943 3174.3171 3168.6883<br>3104.9037 3057.1079 2866.4020 1883.4567 1506.6225 1468.9609<br>1452.5705 1421.6627 1321.1318 1300.1224 1290.3739 1273.5649<br>1242.3560 1200.2865 1172.1465 1148.2666 1035.2623 1006.5412<br>977.5401 959.2109 911.6588 886.0035 864.7377 812.5865<br>751.9995 713.9060 681.0529 658.8081 589.8307 545.7593<br>527.0487 481.1760 439.0283 401.3210 393.8135 373.5421<br>296.0083 251.2027 242.4521 218.8784 193.8550 171.9020<br>120.4592 106.0883 90.1895 60.9948 46.6875 -692.5332	GPS-O_{16}H+$^{\bullet}$OH<br>3888.6547 3871.4759 3636.3767 3596.7597 3563.1548 3161.3541<br>3114.3260 3102.9774 3043.5787 1841.8151 1512.6768 1482.2841<br>1457.9625 1443.7334 1361.0521 1329.3993 1306.4773 1281.5561<br>1252.8704 1231.9256 1209.8994 1052.8665 1014.4454 1008.8904<br>985.7265 932.8216 906.8089 897.9919 867.0931 773.4626<br>760.0983 740.3812 633.2031 600.8038 582.4238 530.9201<br>493.7080 434.2435 426.4262 398.6527 383.4272 348.8095<br>323.6892 303.1919 259.1430 211.3420 201.1209 174.1533<br>154.1656 118.6408 85.9633 69.4673 47.0348 38.5911
GPS-O_{16}+H_2O<br>3930.9955 3881.7198 3872.9337 3833.6451 3645.7767 3207.7983<br>3166.0764 3091.7465 3079.6413 1748.2674 1634.2706 1522.6068<br>1480.2582 1449.4462 1388.8111 1344.5843 1316.1622 1296.2206<br>1280.1263 1226.6141 1187.9173 1050.4483 1010.1333 994.2910<br>969.5786 930.8189 927.0941 902.0453 845.7081 767.2704<br>734.4991 646.5338 516.4866 469.8926 459.1402 443.0198<br>421.9277 385.5431 372.6374 365.4863 341.7193 286.0717<br>279.6024 253.2805 242.3854 196.3769 168.4540 142.2015<br>102.0335 76.7503 47.8601 36.5184 19.4153 -117.2844	GPS-O_{16}—H--$^{\bullet}$OH<br>3889.9740 3868.9707 3739.4071 3629.3914 3175.7741 3165.1033<br>3092.1420 3040.9450 1785.2030 1551.8966 1505.2609 1474.9175<br>1447.0281 1360.4220 1323.6023 1309.6545 1295.5644 1266.4157<br>1230.3708 1199.8110 1059.9540 1013.8499 989.2225 975.5887<br>929.7720 921.4789 906.6464 854.9451 832.2034 757.6137<br>739.5116 683.9193 596.1759 570.0449 513.7922 462.5989<br>443.9306 412.0505 387.3957 379.3362 373.1684 354.4473<br>307.6368 294.3427 232.0346 196.9714 190.2003 147.4554<br>109.2631 102.3275 56.7104 40.2721 31.3637 -2035.3960
GPS(H_2O)-C_3H+$^{\bullet}$OH<br>3889.3763 3868.0941 3813.0735 3658.8705 3596.2861 3529.1429<br>3269.3964 3148.0455 3142.7897 3094.5412 3083.9962 1873.1666<br>1661.2644 1498.5983 1470.2223 1452.7118 1426.5529 1332.4061<br>1312.6288 1300.1924 1288.5258 1219.5706 1180.9765 1176.4523<br>1149.9945 1081.3696 1031.5249 993.7023 978.8105 963.0115<br>938.2683 898.7847 874.1281 826.3061 787.0773 731.4016<br>676.3978 657.1692 647.6090 543.3666 513.4581 492.2824<br>476.9841 457.4283 439.6569 418.9827 389.2765 350.4499<br>303.2710 289.6926 285.7224 242.0044 201.6233 177.6584<br>156.3754 143.0158 120.2190 88.2389 73.7598 58.7730<br>55.0386 41.3728 29.5798 0.0000 0.0000 0.0000	GPS(H_2O)-C_3+H_2O<br>3971.8861 3886.4542 3831.3159 3797.3610 3648.9854 3558.5110<br>3549.7658 3422.5444 3261.5960 3174.5217 3109.3903 1723.6601<br>1656.8841 1632.0367 1604.2345 1472.5294 1458.9643 1378.0874<br>1323.6177 1307.6974 1290.8265 1250.9427 1191.0472 1177.5453<br>1094.8083 1033.5116 1001.3739 984.1830 920.6570 893.6119<br>868.4325 789.0774 759.9944 755.2803 744.1176 681.8481<br>636.8993 582.9668 565.1653 554.9660 518.7430 488.1498<br>449.8524 423.4780 416.4365 394.0729 327.1220 307.8817<br>274.2140 263.0763 247.9617 231.2579 203.3274 193.2963<br>188.6601 175.6316 169.3635 161.3697 119.4024 80.6247<br>65.6204 60.8484 49.3731 0.0000 0.0000 0.0000
GPS(H_2O)-C_3—H--$^{\bullet}$OH<br>3910.0027 3887.3094 3831.9438 3681.8420 3635.6673 3577.4199<br>3566.4452 3165.4987 3147.6053 3067.9764 1858.6050 1621.6814<br>1521.8940 1458.9584 1431.3281 1365.5977 1342.0166 1332.4077<br>1303.1698 1289.7940 1261.2787 1191.9019 1187.5568 1133.3106<br>1078.1152 1008.3574 994.6709 979.9336 962.8087 920.9563<br>890.5640 863.5906 844.9813 811.8435 789.2838 734.4831<br>697.9551 659.2913 622.1016 538.5957 509.3244 479.5304<br>473.4421 441.3852 438.3282 409.2538 386.8090 356.5306<br>324.0526 279.9306 251.4519 221.1543 197.4319 191.0887<br>161.2642 141.8766 128.5557 103.6479 81.7526 71.7386<br>60.0739 27.8671 -1100.5519 0.0000 0.0000 0.0000	GPS(H_2O)-C_6H+$^{\bullet}$OH<br>3896.3157 3850.6632 3823.9131 3730.6429 3728.6753 3531.5916<br>3504.6094 3140.3121 3136.8945 3087.2405 3060.5109 1855.5389<br>1657.4915 1501.9882 1472.1729 1448.1863 1430.7656 1336.6880<br>1314.5236 1300.4239 1289.5710 1225.8223 1185.1272 1183.2789<br>1162.4943 1093.0520 993.7327 986.2104 966.2052 930.3886<br>896.6910 876.1114 857.1927 820.4959 793.2043 671.1800<br>660.0073 651.5593 544.5651 509.3386 491.6464 463.5140<br>442.6685 421.1659 394.5133 361.6386 344.0485 329.0411<br>298.8321 282.8643 278.2937 213.2317 173.3863 162.1656<br>144.5011 137.5921 128.4738 97.2194 75.2666 66.2273<br>58.9998 52.3618 28.7530 0.0000 0.0000 0.0000
GPS(H_2O)-C_6+H_2O<br>3945.8460 3879.0003 3828.6896 3821.4819 3743.8520 3705.2083<br>3575.6338 3293.2002 3245.4089 3142.4346 3040.2390 1893.4215<br>1657.3450 1588.4554 1574.6999 1462.2978 1438.8656 1377.0623<br>1321.0872 1289.5392 1286.1173 1235.1108 1203.5273 1175.0617<br>1112.6693 1019.6263 996.1147 971.1194 961.6891 906.3724<br>882.2214 837.1770 702.1552 691.5806 685.8885 661.3010	GPS(H_2O)-C_6—H--$^{\bullet}$OH<br>3894.3048 3868.9557 3818.1627 3730.7319 3704.6060 3510.8146<br>3375.9169 3145.9844 3096.4247 3089.6857 1875.6790 1648.9517<br>1477.2600 1459.9801 1427.7307 1419.8636 1322.0779 1316.8405<br>1299.8810 1278.0153 1212.1116 1203.0023 1186.9991 1166.2779<br>1114.7588 1103.9406 1008.6769 990.5002 958.2452 938.0066<br>925.5005 890.2931 879.8160 818.4425 760.4938 700.0195

597.0362 563.4561 540.5307 517.8147 502.4599 492.2971
481.0139 447.3956 436.8404 371.3961 309.0243 306.8867
293.3691 275.1828 253.1555 243.3624 208.8910 205.4486
178.6300 156.9956 149.7450 111.2964 82.4263 56.6191
46.0528 40.8874 39.3393 0.0000 0.0000 0.0000

678.0942 661.7746 658.0985 543.3339 526.7364 503.3704
462.0322 441.3798 429.5994 387.6159 339.3215 333.5122
294.3711 289.1104 268.0453 211.4693 204.3491 172.1529
144.8868 132.5179 117.9295 95.6379 69.3030 57.8672
50.6829 37.2530 -1218.4897 0.0000 0.0000 0.0000

GPS(H_2O)-N_1H+•OH
3965.2707 3887.3138 3829.1006 3651.3567 3620.0754 3281.9694
3161.4547 3143.7818 3139.8565 3100.8459 3026.9355 1881.1984
1607.6178 1511.4029 1474.7087 1448.6385 1442.6682 1331.4675
1321.9625 1301.6110 1262.0220 1235.0890 1223.4360 1203.3245
1168.9805 1058.0992 1006.5527 996.6865 983.4446 903.0241
891.5940 865.6783 862.5803 795.3485 748.6964 720.8479
658.1785 635.6058 596.0672 564.8707 530.1880 519.4268
482.9793 472.2212 442.8231 420.6707 387.0598 328.7650
291.2671 257.7983 241.5100 231.6716 220.6446 195.7039
180.0915 164.3418 141.5355 101.6785 83.9041 76.8522
57.8364 44.1958 33.4579 0.0000 0.0000 0.0000

GPS(H_2O)-N_1+H_2O
3953.7096 3888.2699 3819.1992 3792.7647 3701.5573 3177.1601
3145.9431 3140.6854 3067.9150 3035.9100 2861.6433 1876.6849
1680.4924 1621.6382 1472.2194 1455.8908 1398.5563 1353.2086
1303.5932 1275.9504 1269.7560 1244.2770 1216.5330 1180.1884
1148.6514 1041.2047 1025.2497 1009.4087 979.6813 968.1308
939.4124 911.4245 879.2398 859.8302 771.0345 732.3672
672.5997 648.2445 627.2625 535.5957 516.6535 486.7256
475.5120 451.8570 416.9482 401.1140 385.5111 334.4654
322.9114 291.9146 277.4721 247.0887 226.3822 205.5358
174.7985 158.0214 126.0554 110.8214 93.1260 82.6242
62.4956 56.7808 47.6290 0.0000 0.0000 0.0000

GPS(H_2O)-N_1—H--•OH
3952.6477 3887.4615 3820.7988 3692.2087 3177.7369 3160.9061
3154.0500 3091.5474 3083.5784 2885.3379 2565.7263 1880.2875
1620.0391 1530.9501 1458.5362 1444.2956 1418.9605 1375.1699
1323.8951 1313.3801 1273.0633 1261.7758 1245.7871 1206.7623
1173.9301 1112.3019 1074.7740 1033.4422 1023.5323 980.8900
966.9195 913.7101 881.6844 861.4437 815.0123 735.2019
683.9227 661.4319 642.9602 589.6991 540.8586 532.0692
488.4935 450.0447 415.1447 398.7032 381.2694 365.8949
325.8775 283.2666 243.5520 232.1809 227.3006 176.6834
172.4201 153.5708 119.1029 102.3554 90.7569 82.2625
51.3184 44.1303 -677.8781 0.0000 0.0000 0.0000

GPS(H_2O)-O_{16}H+•OH
3888.3990 3855.4204 3721.3687 3636.2020 3583.2244 3521.6878
3496.6038 3138.7531 3134.1075 3088.6077 3067.5241 1832.6284
1649.7555 1502.7752 1473.1323 1453.8470 1445.0928 1341.7400
1337.0271 1297.7356 1289.1940 1253.0884 1214.9612 1186.1540
1163.0031 1096.8424 1004.4155 986.9579 969.0959 937.4087
904.8821 884.5752 868.7946 834.1716 789.7782 774.9128
681.6859 661.0836 571.4830 556.1340 547.9690 509.8519
467.0205 439.5102 424.3388 387.8718 350.1302 344.2162
306.6559 293.0418 269.7534 224.3532 206.7352 190.9848
163.3881 145.5332 140.3267 108.3514 83.1795 68.5906
47.4755 39.2835 26.6961 0.0000 0.0000 0.0000

GPS(H_2O)-O_{16}+H_2O
3982.2548 3933.6185 3891.6597 3837.8729 3743.1300 3570.4226
3425.6779 3217.4906 3157.8399 3096.1427 3076.3005 1846.4431
1637.7877 1585.8736 1536.3136 1472.0658 1452.0031 1363.1568
1321.7855 1302.9263 1283.5213 1263.5935 1198.7634 1189.1989
1169.9015 1071.9892 1004.8733 996.3480 929.8640 902.1337
859.1189 793.9638 790.3713 722.2525 707.0568 643.8026
576.0382 566.3927 518.2914 457.9463 450.3972 437.0162
422.5351 418.1719 359.7403 343.2417 309.2199 289.1676
288.4731 270.6848 221.7680 217.1442 184.1502 170.7725
162.7824 135.8674 112.3559 68.5070 49.1656 39.0084
22.0827 17.6884 -84.4326 0.0000 0.0000 0.0000

GPS(H_2O)-O_{16}—H--•OH
3890.7502 3856.9317 3761.9707 3731.5907 3530.3140 3500.6114
3158.8922 3138.0491 3100.1889 3065.5395 1789.6933 1648.4858
1569.8734 1508.3404 1469.9835 1455.6299 1336.1296 1321.9742
1294.0137 1287.3565 1239.2562 1222.3453 1178.0033 1159.9441
1092.7580 1004.4017 988.2987 962.3926 922.3772 905.7005
896.4084 853.9285 851.3153 827.4645 784.9035 718.8193
655.6257 643.6536 591.5813 534.1674 503.9498 480.1238
439.6926 425.0772 406.2437 378.7240 350.8619 328.3676
299.6329 282.3237 278.3105 218.7826 205.8604 183.6933
155.9661 148.0419 120.1845 102.3239 79.1818 52.0568
42.0543 32.7910 -1933.1036 0.0000 0.0000 0.0000

GPS(H_2O)$_2$-C_3H+•OH
3975.2919 3888.9860 3826.3190 3726.0044 3669.9404 3589.4679
3508.0419 3433.7482 3301.6156 3141.3742 3137.8002 3088.3983
3068.3028 1873.5577 1681.9475 1620.7820 1500.3435 1468.8102
1455.0004 1423.8869 1341.1629 1312.7288 1291.9282 1269.5554
1217.1377 1214.4445 1183.3013 1179.0748 1090.3394 1006.1793
999.8405 967.7848 952.0783 935.7006 907.7060 899.2506
863.6852 842.6972 791.1735 712.8923 666.0283 657.8645
656.0330 609.4117 540.5522 508.4131 479.8453 463.8798
460.0424 437.9413 422.7140 404.0098 339.1647 320.3879
300.0159 287.2409 262.1533 213.7095 201.6974 191.6326
167.8279 159.6312 145.9246 139.3223 125.6580 101.2311
79.1855 68.0283 61.1519 54.2379 49.0015 27.7472

GPS(H_2O)$_2$-C_3+H_2O
3963.1798 3961.7486 3889.0333 3852.8620 3724.9296 3647.3970
3612.5360 3559.1937 3535.4482 3274.9492 3176.4704 3059.2614
2665.3927 1752.3693 1633.0760 1624.2738 1610.2670 1591.8615
1535.3268 1469.0968 1362.3872 1353.2452 1323.7239 1287.6777
1256.5591 1212.4830 1176.0252 1162.5940 1080.2120 1042.4331
984.8946 956.0964 908.0111 869.8393 824.8127 805.3155
783.4052 735.6504 713.7617 696.4080 693.1140 612.3162
593.5130 564.7087 551.3415 508.8382 487.4028 462.0413
424.0630 416.3134 409.4880 359.3998 329.3891 327.0583
304.6349 274.0105 260.1991 255.9743 217.3646 214.4146
187.4127 178.8474 173.7440 161.0299 137.1591 114.8761
93.8721 82.1781 71.2906 54.4036 43.4651 30.3316

GPS(H_2O)$_2$-C_3—H--•OH
3959.0536 3889.1179 3827.6800 3820.2632 3770.8652 3647.8384
3596.2984 3395.2489 3164.9527 3128.0228 3093.5244 2807.6808
2171.2234 1896.6529 1630.7644 1601.6058 1538.4919 1448.0586
1422.7672 1373.9724 1348.9900 1318.0633 1300.3345 1272.2454
1239.6004 1215.7212 1162.9012 1119.3816 1065.7349 1026.4099
1007.8992 984.8100 977.8040 902.0615 887.4679 863.6218
839.0165 778.2820 756.3672 706.5123 667.3836 651.0034
627.7950 612.6444 538.5902 523.6299 498.7846 475.8930
466.4703 437.9831 435.6592 372.3708 364.8081 323.7271
308.8649 288.4602 261.4884 248.8608 240.7892 213.9115
207.1997 161.2442 150.7559 133.9079 114.7574 111.7465
89.4072 73.5574 58.1457 50.6250 37.2343 -541.1013

GPS(H_2O)$_2$-C_6H+•OH
3970.2804 3892.4305 3809.1919 3780.1120 3728.2089 3685.4026
3512.7886 3496.9807 3132.0917 3129.2317 3083.2926 3082.6713
3058.9811 1856.2171 1679.3965 1618.2732 1529.5562 1471.9570
1448.6514 1431.2606 1340.5962 1314.2652 1293.3018 1284.5123
1238.0528 1233.4641 1192.2629 1183.5992 1090.1076 1046.9178
1018.3155 1001.4356 969.4775 934.6200 910.4274 876.7992
844.5676 822.5740 790.4860 727.2254 681.4240 665.4974
583.4987 538.4505 516.7406 481.2610 470.5606 454.2285
429.5904 425.0575 414.8652 355.7366 336.5772 307.8474
301.9535 289.7014 284.8020 241.1213 226.0137 214.2915
185.8816 171.2352 148.1413 137.4333 114.7735 107.5799
91.6650 73.5455 61.9224 55.9158 45.0616 29.3574

GPS(H_2O)$_2$-C_6+H_2O

3967.5775 3959.6608 3884.9406 3829.7162 3822.7272 3799.5774
3696.8282 3585.3793 3385.1151 3240.5581 3224.9712 3152.9643
3060.8672 1891.8763 1635.0773 1615.7086 1607.1323 1587.2295
1465.5805 1431.9911 1404.7320 1326.4961 1305.6734 1291.5243
1245.6739 1223.4295 1174.4847 1128.2223 1029.5752 1006.7558
1001.6425 955.7542 901.7002 878.6571 855.6372 840.5696
735.0742 685.9378 651.1096 640.8366 586.1394 557.4368
525.4786 510.6553 487.3732 474.8746 459.5431 450.7545
436.8901 391.7847 369.3401 322.3479 305.4472 293.8770
281.8091 279.3783 277.2507 244.1242 235.8117 208.0951
196.2804 189.0979 141.2114 131.3406 124.5003 102.9528
88.3780 79.2372 66.8020 43.3201 39.3253 25.3284

GPS(H_2O)$_2$-C_6—H--•OH
3963.8299 3888.5155 3813.9316 3752.0020 3721.9066 3699.2091
3547.3098 3475.8726 3141.0101 3092.4712 3079.7370 2893.3873
1876.4352 1680.8740 1604.0570 1501.3713 1465.4317 1452.2058
1427.1534 1331.9621 1321.0526 1316.3211 1280.4778 1252.3839
1228.4780 1206.0242 1170.6189 1153.2397 1132.5720 1107.3749
1019.4952 1005.6361 968.7846 937.4245 906.5866 880.5538
838.0326 801.7147 763.4595 715.2233 690.1341 661.8610
653.4842 595.8972 540.4402 512.9236 475.0648 469.3074
435.7646 411.1469 406.7744 358.8881 346.4609 309.9302
297.1351 287.3723 282.0818 245.4462 221.1749 197.1714
168.7013 156.7955 124.6131 121.4377 105.6617 90.8975
77.1116 63.0130 51.4379 37.0255 36.3497 -1158.0029

GPS(H_2O)$_2$-N_1H+•OH
3956.8496 3894.0311 3829.6829 3794.1139 3703.3791 3530.7928
3518.1746 3271.8182 3139.3386 3137.9555 3089.4226 3074.5811
2982.9102 1874.2351 1714.0415 1632.2584 1512.9766 1471.6537
1451.9800 1426.7669 1338.4439 1313.2741 1297.8894 1293.6874

GPS(H_2O)$_2$-N_1+H_2O
3968.0880 3881.2064 3820.3600 3812.3357 3790.8685 3682.3145
3488.8707 3456.1002 3138.7322 3132.8611 3065.8840 3029.8448
2446.8956 1873.5022 1717.4526 1655.6694 1616.5276 1472.2427
1457.6387 1400.0212 1353.5944 1307.5231 1268.3194 1261.5599

1235.6704 1224.7408 1184.9687 1173.3074 1104.7463 1084.1870
1022.7784 1002.3615 970.2572 954.1046 913.0142 879.9006
875.0221 855.3187 791.9316 736.3414 668.3010 657.3255
647.7024 608.3196 541.9275 540.9900 513.2304 497.7438
468.6328 458.4954 451.2359 430.6400 353.2216 320.4979
303.4480 287.9271 272.2319 257.8733 253.0048 203.0031
196.2324 177.6770 161.2690 147.2637 138.5586 120.9968
98.8109 83.1531 66.5933 59.2152 55.8870 34.8615

1251.8174 1236.9007 1209.3757 1176.0566 1145.0403 1040.5724
1011.7015 994.9202 975.2195 914.2540 910.0299 878.0311
857.8738 804.7536 734.0667 709.3619 677.5422 673.7173
648.5547 599.5204 540.9726 530.3505 511.5013 479.7143
470.7036 460.8669 430.7220 406.3049 379.3205 357.1916
336.5919 305.5014 275.8166 261.4140 239.5395 212.8471
204.8126 188.3266 163.1022 151.0854 145.2407 103.9055
101.5549 77.6232 62.2381 44.3766 41.3454 35.3368

GPS$(H_2O)_2$-N_1—H--•OH
3965.1537 3886.3611 3816.7233 3776.6706 3711.2641 3622.3240
3459.5856 3148.2272 3118.1039 3077.4518 3051.2612 2693.4501
2572.0595 1871.9946 1683.0563 1607.1544 1463.0407 1434.7685
1428.8560 1407.3863 1346.8899 1338.2249 1311.5185 1269.5407
1255.8681 1244.2658 1209.9993 1185.1784 1142.8192 1085.5452
1043.7932 985.6398 974.7492 912.9487 873.2500 863.4151
849.3500 817.4379 767.0182 724.4137 673.7619 665.1210
658.0871 581.5941 533.6167 516.3881 482.6260 478.3123
445.9526 426.3500 415.6874 379.9143 346.3352 305.7831
300.1107 282.4027 259.9612 248.3088 237.2884 221.5320
201.4398 184.4900 175.1764 159.1904 127.1295 105.4119
91.9955 84.0264 65.9945 50.7811 37.9391 -727.2707

GPS$(H_2O)_2$-O_{16}H+•OH
3963.7370 3889.3661 3771.2360 3698.7571 3630.2113 3577.4205
3520.6854 3504.2438 3136.3667 3134.2236 3082.1935 3064.5421
3043.1832 1831.5680 1677.5148 1610.0588 1531.0453 1469.6251
1451.4939 1446.8611 1346.0352 1341.1643 1289.8588 1281.8891
1254.8225 1235.9728 1218.3621 1187.2048 1093.6619 1062.8765
1018.0111 1001.6449 971.2761 936.7462 910.8433 885.9608
843.3004 811.3849 788.9423 775.0552 724.8344 681.9670
599.1040 588.4277 556.3870 546.0082 474.2337 472.2658
441.5723 427.7646 408.5506 345.5411 334.6099 323.1013
305.6288 298.2172 285.1559 242.5172 223.0033 208.5249
200.0578 165.5123 150.7020 137.9829 124.8999 104.0867
98.3633 84.4940 56.9015 49.0295 43.5112 30.0498

GPS$(H_2O)_2$-O_{16}+H_2O
3956.7708 3951.0579 3925.3436 3880.7039 3833.3747 3600.0059
3545.2206 3457.2929 3215.8213 3189.4572 3174.2679 3100.9173
3070.3789 1775.7371 1635.4117 1626.7463 1592.5066 1552.4740
1478.5788 1451.6668 1381.8059 1324.1093 1293.9307 1277.6033
1275.8217 1231.6965 1214.8806 1169.7973 1039.2292 1021.1378
980.3905 939.7288 923.7466 911.4755 865.7048 849.0797
822.6216 768.0898 733.4906 671.3942 646.3603 579.7854
519.5650 504.5687 497.5777 469.7412 450.3185 420.8931
406.9368 390.4885 373.3334 360.3046 335.8390 281.6739
270.5030 248.3371 245.8462 236.8395 215.9814 200.5075
185.5348 171.8768 140.8721 129.0696 96.3706 83.9540
79.7971 47.5140 38.3896 34.1944 19.4443 -105.7801

GPS$(H_2O)_2$-O_{16}—H--•OH
3967.2599 3892.9799 3798.7205 3742.6174 3733.4956 3531.4304
3500.7862 3154.1975 3127.2226 3095.6672 3095.0101 3061.3430
1790.8086 1672.1611 1606.2720 1553.8386 1532.2889 1468.5600
1456.9811 1345.4104 1323.9067 1291.2633 1284.2796 1240.9343
1236.6710 1227.1197 1180.8531 1091.6528 1044.9018 1020.2177
999.2549 965.6480 919.8291 913.8143 901.6973 847.4912
839.6472 808.5767 786.4275 728.8263 708.8538 655.7871
588.9862 554.7785 518.2885 481.1998 467.9392 435.7793
419.6676 402.7635 379.8125 374.3006 334.0133 315.7016
295.1689 280.7792 277.6193 242.4130 217.7639 204.5825
186.8532 142.5521 121.2245 116.3633 110.8665 96.8296
70.7366 58.3735 49.3250 43.5474 36.3941 -1998.9485

GPS$(H_2O)_3$-C_3H+•OH
3975.2057 3973.6751 3818.3066 3769.5536 3712.5308 3677.4834
3596.4977 3503.2814 3452.5367 3339.6915 3191.7681 3138.4474
3137.4129 3087.6192 3059.5578 1872.6692 1683.0720 1625.4127
1589.9625 1499.1080 1470.6896 1455.7179 1424.7117 1342.5555
1312.9925 1291.5816 1257.7346 1227.9572 1219.5537 1184.4778
1181.8246 1167.8786 1090.8393 1031.3978 975.5653 961.6114
956.0294 949.1019 930.1506 869.5044 848.2582 798.7440
728.1583 706.8470 665.7021 659.3950 646.3287 611.9260
596.3557 549.5386 512.0529 480.8433 473.2102 468.9206
436.4108 425.3862 403.6644 400.0781 335.5348 318.6398
310.6468 286.9364 269.7253 258.4302 225.5972 206.1289
198.6165 186.8512 177.9406 157.9485 147.0813 136.1544
125.4329 97.4584 71.7127 67.4519 63.8580 51.4780
43.2294 28.1295 22.1894 0.0000 0.0000 0.0000

GPS$(H_2O)_3$-C_3+H_2O
3966.3393 3961.3788 3953.6289 3846.9279 3759.9489 3699.5279
3688.9655 3647.4669 3579.3502 3534.7250 3428.6866 3263.3428
3189.3692 3049.8766 2643.7459 1754.8323 1646.1910 1620.5152
1614.2023 1598.8603 1587.5007 1546.5736 1469.3455 1367.6256
1360.2602 1322.4106 1287.7980 1238.8311 1213.1132 1170.1043
1160.0990 1156.5227 1075.8603 1063.4275 964.0179 944.9691
873.1498 813.1874 803.2384 774.8739 742.2258 729.9659
699.8456 696.1832 685.2917 653.4337 643.0549 621.9266
562.9501 553.7612 516.1264 506.5975 473.1824 440.3711
427.8685 417.8937 378.0742 349.9336 323.1676 303.7025
286.3893 281.8191 277.9610 272.7676 255.5308 251.4693
236.5522 214.0373 209.3060 184.0713 167.0212 158.7859
145.6518 121.1130 99.1921 84.1101 69.5210 60.1930
50.5808 43.9532 28.7085 0.0000 0.0000 0.0000

GPS$(H_2O)_3$-C_3—H--•OH
3977.0689 3972.4736 3820.4371 3775.1278 3714.9564 3669.8530
3597.4849 3565.4223 3452.6278 3434.2142 3311.1732 3147.5741
3132.3093 3088.3854 1868.0248 1662.3175 1627.0211 1592.3683
1498.6377 1476.8331 1452.8149 1413.8174 1332.5826 1317.3980
1295.5727 1252.6351 1234.8896 1222.9999 1201.6574 1178.7115
1165.1084 1069.2460 1023.4396 971.3891 963.3494 951.2223
934.0205 914.1693 872.5545 859.7280 791.7211 778.0284
724.5657 705.5799 670.3955 665.7664 615.2480 606.6929
577.1478 551.2885 536.5409 518.1681 479.3724 458.6981
446.9944 415.4307 401.8952 398.7642 338.6309 314.0623
300.8120 285.7828 274.6582 269.9523 252.8766 220.7494
204.1848 189.7895 172.8704 162.9410 147.4125 135.1256
114.8548 98.5567 78.3368 59.8937 53.7748 42.0080
27.8847 24.6026 -1293.6255 0.0000 0.0000 0.0000

GPS$(H_2O)_3$-C_6H+•OH
3970.7804 3969.4598 3767.8319 3714.1878 3655.2363 3633.3115
3580.2380 3541.2323 3493.0129 3435.9032 3134.9040 3120.0292
3084.1689 3053.9421 2954.9079 1832.7829 1685.1608 1614.4164
1591.0180 1524.6547 1468.4010 1448.8606 1447.1676 1344.8137
1338.8553 1288.5458 1285.8828 1256.0394 1239.8519 1218.7304
1188.2018 1171.3133 1094.2334 1089.9945 1041.9294 975.2302
951.7327 937.9752 886.4687 844.9384 818.3886 800.5521
789.6952 735.9105 720.5725 682.5025 648.9948 603.4015
570.4960 554.1371 549.5626 494.8861 477.9758 450.6946
426.0281 400.7186 397.7248 360.7819 337.4025 312.9655
305.2098 295.6457 290.9731 279.8535 246.3051 233.8252
223.5210 203.0894 196.3576 160.5482 155.2980 136.6373
120.7769 102.0476 90.3172 77.6077 56.9987 49.4054
43.8140 32.1558 19.8989 0.0000 0.0000 0.0000

GPS$(H_2O)_3$-C_6+H_2O
3974.7654 3966.0780 3952.5149 3827.5551 3817.0568 3790.5689
3702.7622 3700.2327 3577.0686 3482.2425 3383.7612 3233.5114
3141.2297 3105.7215 3072.7983 1893.5024 1631.3601 1613.9865
1600.6433 1595.8138 1587.0206 1459.6830 1419.2800 1403.0769
1325.2455 1304.4790 1279.7305 1239.1394 1218.2430 1176.8934
1161.8654 1130.8964 1038.3396 1009.5695 1000.6518 939.9002
873.3381 843.2011 838.8214 744.0389 715.3058 678.5843
670.0232 656.1866 604.8307 575.8698 553.3395 532.3048
513.9990 488.0847 484.6922 468.4504 460.0216 419.2200
397.5087 383.8403 344.0147 322.6307 313.7215 299.8615
292.9687 281.4555 273.0502 243.8850 237.8155 217.1809
204.7628 202.6645 195.5590 160.5607 140.5033 135.5993
120.6173 107.6416 78.6505 69.6398 63.2169 46.7810
41.3482 24.1394 18.0085 0.0000 0.0000 0.0000

GPS$(H_2O)_3$-C_6—H--•OH
3967.7788 3966.6141 3820.4291 3739.4826 3734.4809 3721.0866
3681.6658 3533.5503 3492.1075 3412.8399 3136.8738 3088.7085
3084.4511 2831.8989 1878.3463 1674.3884 1613.8955 1591.4939
1498.2748 1468.3113 1453.0877 1427.2168 1341.4435 1318.4508
1312.2558 1285.5927 1246.3447 1233.3547 1215.4639 1182.0618
1179.0261 1161.4877 1155.6688 1106.4639 1033.9888 1006.2147
951.8674 920.4500 879.8707 837.7793 803.5303 761.6775
730.7668 711.7153 683.0503 666.7880 647.2238 630.8477
608.0170 551.0566 517.2664 486.9863 480.9858 438.4136
420.3095 399.9298 367.0394 341.3326 330.9978 307.0233
297.2175 291.1859 267.9609 242.2134 230.8778 221.7991
219.0254 195.2839 167.2444 149.5472 131.7610 121.1736
108.3867 85.9329 74.5569 63.0034 56.9776 47.3447
28.3819 23.2204 -1159.9241 0.0000 0.0000 0.0000

GPS$(H_2O)_3$-N_1H+•OH
3971.9482 3955.9137 3820.9326 3802.5529 3726.8945 3640.2519
3552.2879 3526.8959 3448.5646 3310.0493 3143.6392 3127.1430
3092.6349 3062.6455 2914.6702 1874.9589 1674.1744 1635.9102
1589.5239 1493.1986 1467.8125 1449.0188 1422.7455 1339.3657
1314.6340 1296.1537 1290.6077 1235.6272 1227.1493 1185.2959
1177.7745 1164.8535 1097.8890 1092.1897 1046.5520 974.3657

GPS$(H_2O)_3$-N_1+H_2O
3973.0727 3965.5958 3827.1363 3818.0414 3801.1988 3729.0226
3667.7681 3487.1338 3434.8066 3430.8732 3140.4606 3136.0262
3062.8869 3027.1056 2476.8166 1877.4034 1713.3427 1659.2992
1623.2749 1587.8317 1474.3581 1457.6569 1401.7690 1328.6942
1308.9945 1270.3557 1254.2823 1247.2045 1228.4323 1209.7482
1178.2185 1173.5644 1148.3571 1058.4194 1012.2313 976.4129

953.9877 944.2082 879.2373 874.3167 839.4931 797.3025<br>739.2970 713.6482 670.0497 654.2861 652.5189 622.8418<br>598.6037 558.2569 547.3663 522.6978 510.9014 483.2732<br>462.7418 448.0841 412.1754 373.9367 340.8557 313.9214<br>309.2271 290.8122 286.3610 255.9651 250.5410 235.4832<br>209.1011 199.8190 189.5625 165.5927 156.2012 135.8931<br>126.1218 118.2235 77.2704 71.9472 58.8354 58.2324<br>48.8845 35.3969 21.5874 0.0000 0.0000 0.0000	952.1910 918.7386 878.2069 855.7030 798.1195 738.9025<br>714.8522 698.2641 676.2587 666.8773 644.4339 595.7494<br>585.9780 544.7622 530.9116 513.8422 493.9825 467.7494<br>458.4692 431.1882 404.1152 380.3932 378.2649 340.5652<br>313.0881 302.4268 289.0752 270.9490 249.0296 238.5913<br>214.7984 201.8335 193.6711 172.3760 158.4720 145.1681<br>137.4530 104.0071 92.0798 76.2407 59.0771 48.1968<br>41.3287 34.7533 22.1558 0.0000 0.0000 0.0000
GPS$(H_2O)_3$-N_1—H--$^{\bullet}$OH<br>3968.0598 3956.0501 3854.9527 3813.7883 3767.9100 3719.0973<br>3607.0340 3498.2363 3440.7565 3348.2995 3130.1401 3097.4910<br>3077.6878 3063.6877 3052.0964 1860.4135 1667.2206 1634.9768<br>1600.5493 1509.3807 1470.5544 1441.0628 1418.7951 1346.8971<br>1314.8404 1286.7273 1265.1009 1257.3732 1219.5906 1199.7787<br>1184.3391 1151.3073 1100.6987 1035.9674 1002.0678 979.4553<br>943.0175 873.0175 866.6162 843.3806 796.7910 784.9027<br>743.8454 700.6067 670.8469 658.8481 650.3924 577.7051<br>552.8730 526.1526 522.1053 492.8082 485.3176 476.1121<br>435.3627 407.2328 381.7292 352.9444 339.5794 313.2311<br>302.6133 295.5719 269.9388 261.6517 255.3747 228.0782<br>215.1862 193.1576 187.5913 176.2918 162.3217 140.2808<br>135.3514 121.4456 90.6182 79.4040 71.7519 53.3673<br>51.9702 33.7685 -29.4842 0.0000 0.0000 0.0000	GPS$(H_2O)_3$-O_{16}H+$^{\bullet}$OH<br>3970.8462 3969.6191 3767.7489 3715.2293 3656.6557 3633.2824<br>3580.1779 3542.0475 3493.1751 3438.0679 3134.9517 3120.0186<br>3084.1973 3054.0709 2957.9027 1832.7866 1685.3002 1614.3676<br>1591.3921 1524.7389 1468.4057 1448.8340 1447.1554 1344.7799<br>1338.8473 1288.5135 1285.2148 1256.0269 1239.8051 1218.7495<br>1188.2972 1171.4461 1093.7933 1089.2218 1041.6790 975.2296<br>951.6837 937.8789 886.3909 844.7908 818.3083 800.3921<br>789.6259 735.1142 719.8326 682.4435 648.2466 602.7891<br>570.3508 554.0888 549.4432 494.8226 477.7411 450.5066<br>425.8451 400.9864 397.5704 362.2513 337.3177 313.1646<br>305.0816 298.9335 290.7330 279.0779 246.2320 233.9477<br>223.3755 202.9778 196.2266 160.5406 155.2847 136.5923<br>120.7183 101.9701 90.3376 77.6799 57.0965 49.2906<br>43.7646 32.0738 19.9684 0.0000 0.0000 0.0000
GPS$(H_2O)_3$-O_{16}+H_2O<br>3948.9736 3947.3132 3770.5064 3759.5087 3716.6513 3680.2817<br>3499.4566 3365.3276 3194.7559 3153.5223 3087.1869 3061.5695<br>3001.4389 2878.4596 2458.3392 1778.0577 1688.2925 1677.6692<br>1645.9447 1634.0729 1572.3148 1483.9264 1434.7712 1430.3526<br>1393.1968 1320.2633 1301.3045 1271.9785 1246.4023 1199.9467<br>1189.6242 1175.1792 1147.1187 1131.1548 1076.0135 1041.4924<br>1019.8571 980.2032 957.4504 943.1091 843.5224 830.0941<br>799.2116 778.9674 757.2016 741.4034 704.7677 656.3356<br>622.2573 615.3524 570.7333 554.4443 512.1512 470.2376<br>458.4501 441.2972 421.6441 392.5747 379.3554 362.5263<br>353.5732 347.2999 332.4857 313.8314 286.5566 282.4491<br>275.6454 217.0297 203.1840 186.7341 173.3811 168.2867<br>155.7281 131.1713 104.8446 95.7795 72.7837 62.5924<br>49.5757 6.6005 -101.4285 0.0000 0.0000 0.0000	GPS$(H_2O)_3$-O_{16}—H--$^{\bullet}$OH<br>3974.2606 3965.2865 3777.1272 3732.9589 3725.5045 3722.6350<br>3525.3244 3498.5013 3427.7434 3160.2852 3130.6523 3099.8187<br>3058.2621 3027.3564 1787.8280 1676.9768 1619.1917 1595.1774<br>1567.9756 1531.1405 1467.5540 1455.1793 1341.0300 1327.6998<br>1289.4461 1270.2654 1237.1929 1231.7768 1230.0902 1181.4124<br>1166.1814 1091.8729 1072.9562 1038.7938 972.1765 949.2814<br>913.3283 904.3763 848.3598 842.7265 807.8043 791.1773<br>737.7892 727.3662 702.5899 641.6219 635.2911 583.6217<br>560.7233 528.0255 482.6599 467.1761 441.0274 416.5834<br>397.5463 391.1936 381.7293 374.2566 325.8346 311.6352<br>306.3113 293.5913 283.8493 271.2173 243.2965 235.8825<br>219.4309 197.3467 190.2767 156.7294 139.2104 122.5813<br>109.2922 99.2251 81.3239 66.5341 49.4744 45.0344<br>34.4702 24.5237 -1974.0420 0.0000 0.0000 0.0000
